# Defense Compounds Rather Than Nutrient Availability Shape Aggressiveness Trait Variation Along a Leaf Maturity Gradient in a Biotrophic Plant Pathogen

**DOI:** 10.3389/fpls.2018.01396

**Published:** 2018-09-28

**Authors:** Agathe Maupetit, Romain Larbat, Michaël Pernaci, Axelle Andrieux, Cécile Guinet, Anne-Laure Boutigny, Bénédicte Fabre, Pascal Frey, Fabien Halkett

**Affiliations:** ^1^Université de Lorraine, INRA, IAM, Nancy, France; ^2^Université de Lorraine, INRA, LAE, Nancy, France; ^3^ANSES, Laboratoire de la Santé des Végétaux, Unité de Mycologie, Malzéville, France

**Keywords:** constitutive defense, aggressiveness traits, plasticity, nutrient availability, rust fungus

## Abstract

Foliar pathogens face heterogeneous environments depending on the maturity of leaves they interact with. In particular, nutrient availability as well as defense levels may vary significantly, with opposing effects on the success of infection. The present study tested which of these factors have a dominant effect on the pathogen’s development. Poplar leaf disks of eight maturity levels were inoculated with the poplar rust fungus *Melampsora larici-populina* using an innovative single-spore inoculation procedure. A set of quantitative fungal traits (infection efficiency, latent period, uredinia size, mycelium quantity, sporulation rate, sporulation capacity, and spore volume) was measured on each infected leaf disk. Uninfected parts of the leaves were analyzed for their nutrient (sugars, total C and N) and defense compounds (phenolics) content. We found that *M. larici-populina* is more aggressive on more mature leaves as indicated by wider uredinia and a higher sporulation rate. Other traits varied independently from each other without a consistent pattern. None of the pathogen traits correlated with leaf sugar, total C, or total N content. In contrast, phenolic contents (flavonols, hydroxycinnamic acid esters, and salicinoids) were negatively correlated with uredinia size and sporulation rate. The pathogen’s fitness appeared to be more constrained by the constitutive plant defense level than limited by nutrient availability, as evident in the decrease in sporulation.

## Introduction

Plant diseases and pests are responsible for crop damage that may account for up to 40% of yield losses worldwide (Boonekamp, 2012). Pathogen aggressiveness, the quantitative component of pathogenicity (Pariaud et al., 2009a), has been widely studied in order to forecast the evolutionary potential of plant pathogens and to better design disease control strategies (Lannou, 2012). Aggressiveness can be evaluated at different scales: by measuring the epidemic rate at the field scale or through elementary phenotypic traits at the individual scale (Pariaud et al., 2009a). The environment plays a key role in developmental processes of the pathogen and influences the individual’s phenotype and the scale of fitness (Scheiner, 1993; Jarosz and Davelos, 1995). Biotrophic fungal pathogens, such as rust and powdery mildew fungi, spend most of their life cycle within living plant tissues, so their fitness is directly affected by leaf physiology (Agrios, 2005; Gamm et al., 2011; Duplessis et al., 2013). On the one hand the leaf tissue provides the pathogen with the necessary nutrient resources for its development. On the other hand, it impedes pathogen development through a battery of plant defense compounds (Ullah et al., 2017).

Studies on interactions between plants and their foliar pathogens have shown that plant age, and especially leaf age, leads to different responses of the pathogen. Coleman (1986) postulated from a series of experiments that leaves pass through a peak of susceptibility to disease and pest attack. He proposed that for many stresses, including infection by biotrophic fungi, this peak coincides with changes in leaf properties during the transition from metabolite sink to source. This stage corresponds to a maximal nutrient availability. After this sink-source transition, free sugar content and thus nutrient resources decrease with increasing leaf maturity (physiological stage at a relative leaf age). Many studies support this view, demonstrating a maximum disease level before leaves are fully expanded and a decrease of disease severity on older leaves, which is also referred to as ontogenetic resistance (Ficke et al., 2002; Xavier et al., 2015; Farber and Mundt, 2017). In agreement, Merry et al. (2013) demonstrated that the peak in the severity of powdery mildew along a primary shoot of *Vitis vinifera* corresponds to the position of the first leaf that does not import carbohydrates. However, some studies have reported a contrasting pattern, e.g., Knott and Mundt (1991) found that infection efficiency of the wheat leaf rust fungus *Puccinia recondita* f. sp. *tritici* is 25% higher on older than on younger leaves in five wheat cultivars. The opposite trend may involve the dark side of plant-pathogen interaction: defense compounds. Their actions on a pathogen’s development have been studied in several pathosystems, including rust fungi infecting poplar (Johnson and Kim, 2005), black currant (Vagiri et al., 2017), and willow (Hakulinen et al., 1999). Phenolic compounds present in leaves appear to highly influence the general plant resistance to fungi (Adandonon et al., 2017; Ullah et al., 2017). Most studies have focused on plant susceptibility to the disease (i.e., infection efficiency of the pathogen) or focused on overall disease severity. To dissect which fungal function is most influenced by leaf maturity and how, we propose to measure a set of elementary traits underlying components of aggressiveness.

Foliar pathogens of woody plants are good biological models to study the influence of leaf age on disease development, because they can infect leaves at various levels of maturity on the same host plant (Cellerino et al., 1978; Sharma et al., 1980; Turechek and Stevenson, 1998). Poplar is particularly well suited for studying the effect of leaf age on pathogens, since poplar plants grown from cuttings in a glasshouse produce new leaves at a constant rate, giving access to a wide range of leaves of increasing maturity on the same plant (Larson and Isebrands, 1971). These authors introduced the concept of leaf plastochron index (LPI) to refer to a position of a leaf along a poplar shoot. The youngest fully expanded leaf corresponds to LPI 1. Older leaves are numbered with increasing LPIs, according to their position along the shoot. In most experimental conditions the sink to source transition in poplar is established around LPI 6 (Coleman, 1986), which thus defines the first mature leaf. The effect of leaf maturity on the development of poplar pathogens (*Marssonina brunnea, Melampsora medusae*, and *M. larici-populina*) has been widely studied (Cellerino et al., 1978; Sharma et al., 1980; Coleman, 1986; Giorcelli et al., 1996). In most of these experiments, leaves were spray-inoculated with spore suspensions. In these conditions, hundreds of spores compete for infection, and as a result, inoculum density has been shown to influence aggressiveness traits (Giorcelli et al., 1996; Pei et al., 2003; Pariaud et al., 2009a). A way to eliminate any possible interaction among spores and to remove the effect of lesion density on trait variation is to use a single-spore inoculation protocol. Moreover, the single-spore inoculation enables the examination of phenotypic variation at the individual lesion level and not by means of average trait values measured on a cohort of lesions.

The aim of this study was to assess the effect of leaf age, and the associated content in nutrients and defense compounds, on the variation in aggressiveness traits in the poplar rust fungus *M. larici-populina.* We quantified trait variation along the leaf maturity gradient using the same fungal genotype to establish a so-called reaction norm (Gavrilets and Scheiner, 1993). We measured the effect of LPI using a single-spore inoculation method for a suite of aggressiveness and morphological traits: infection efficiency, latent period, uredinia size, sporulation rate, sporulation capacity, *in planta* mycelium quantity, and urediniospore volume. In a first step, we examined the differences in pathogen development along a leaf maturity gradient in order to study the variation in fungal traits and to assess the correlations between them. In a second step, we estimated the influence of the most important leaf compounds (sugars, total carbon and nitrogen, and phenolics) on the variation in aggressiveness traits.

## Materials and Methods

### Plant and Fungal Material, and Experimental Design

Characterization of quantitative traits was performed on excised leaf disks of *Populus deltoides* ×*P. nigra* ‘Robusta’. Poplar plants were grown from dormant cuttings in 10-L pots containing a sand-peat (1:1, v/v) mixture, with an initial fertilization of 3.5 g L^-1^ CaCO3 and 6 g L^-1^ of slow release 13-13-13 N-P-K fertilizer (Nutricote^®^ T100, Fertil). The plants were grown in a growth chamber regulated at 20/22°C (night/day temperatures) and 16 h photoperiod in order to ensure optimal growth, and were watered daily with deionized water. After four months, plants were about 1.2 m high and exhibited 25 to 30 fully expanded leaves.

On the same day, we harvested eight leaves of different maturity (LPI 6, 8, 10, 12, 14, 16, 18, and 20) from each of three plants (total of 24 leaves). From each poplar leaf, 24 disks (12-mm-diameter) were excised, and placed in flotation, each in a different cell culture plate. Each plate consists in 24 wells and thus contained one sample (leaf disk) of all leaves. A plate thus corresponded to a complete statistical block: Twenty-four replicate plates were prepared, each containing the 24 combinations of LPI and plants. Within plates, the positions of the samples were randomized (**Supplementary Figure S1**). After disk excision, the remaining part of each leaf (about half a leaf) was stored individually in an aluminum wrapper at -80°C for sugar, nitrogen, carbon, and phenolic compound quantification. We hereafter refer to this plant material as “uninfected leaves.”

Because the single-spore inoculation protocol is highly time-consuming, we were not able to inoculate more than six plates per day. Inoculations of the 24 plates were thus distributed over four consecutive days (each inoculation day formed a series of six plates). In other words, all leaf disks were excised the same day (day 1), but inoculated after 0 to 3 days depending on the series they belonged to.

Our experimental design (randomized complete block design) thus consisted in three experimental factors: LPI, plant and day of inoculation. Given an infection efficiency of approximately 25%, we expected six replicates per combination.

### Single-Spore Inoculation Protocol

Single-spore inoculation was performed with pure *M. larici-populina* isolate 98AG31. This isolate was collected in 1998 in Moÿ-de-l’Aisne (France) on *Populus trichocarpa x Populus deltoides* ‘Beaupré’ leaves and serves since as a reference isolate (Duplessis et al., 2011). Urediniospores were cryopreserved at -80°C at Institut National de la Recherche Agronomique Nancy (France) and multiplied on fresh poplar leaf disks before inoculation to maximize their germination and infection rates (Pei et al., 2002). To this aim, 1 mg of urediniospores was dispersed in 200 μL of water-agar (0.1 g L^-1^). The resulting urediniospore suspension was applied as 2 μL droplets on the abaxial surface of two 30-mm-diameter leaf disks of ‘Robusta’. Leaf disks were incubated by floating on deionized water in six-well polystyrene cell culture plates, with the abaxial surface upside, at 19 ± 1°C, and under continuous fluorescent light (25 μmol m^-2^ s^-1^). After a 10 to 13 days incubation period, the sporulating leaf disks were gently tapped over a microscope slide to release and collect urediniospores.

A germination test was performed just before inoculation to ensure the quality of urediniospores used. To this aim, a few hundred urediniospores were dispersed on the surface of a Petri dish containing agar (20 g L^-1^). After an overnight incubation at 19 ± 1°C, the ratio of germinated/total urediniospores was evaluated under a light microscope (100× magnification) to determine the germination rate. This test ensured that nearly all spores used for the experiment were physiologically able to germinate (mean germination rate of 93%).

For single-spore inoculation leaf disks were inoculated one by one. One single urediniospore was picked with a human eyelash under a stereomicroscope (63× magnification) and deposited into a 5 μL water-agar droplet placed at the center of the poplar leaf disk (abaxial surface). After inoculation the plates were incubated for 13 days in the same conditions as described above.

### Quantitative Trait Measurements

Several aggressiveness traits classically studied in plant-pathogen interactions were measured: infection efficiency, latent period, uredinia size, sporulation rate, and sporulation capacity (Lannou, 2012). In addition, we measured *in planta* mycelium quantity, which is less commonly studied.

• *Infection efficiency* is commonly defined as the probability that a urediniospore deposited on a receptive host surface produces a lesion (Lannou, 2012).
• *Latent period* is defined as the time interval between infection and the onset of sporulation from that infection (Lannou, 2012).
• *Uredinia size* is defined as the surface area that produces the urediniospores (Kolmer and Leonard, 1986; Robert et al., 2004) and has been suggested to be an indication of the pathogen capacity for host tissue colonization (Pariaud et al., 2009b; Lannou, 2012). However, the uredinia size is not a proxy of tissue colonization by *M. larici-populina*, as no necrosis is observed. Unlike necrotrophic pathogens, the only visual symptom is the opening in the leaf epidermis, through which urediniospores are extruded.
• *Sporulation rate* is defined as the number of urediniospores produced by the uredinia per unit of time (Leonard, 1969; Clifford and Clothier, 1974; Kardin and Groth, 1989): sporulation rate = urediniospore number/(13 – latent period).
• *Sporulation capacity* is defined as the amount of spores produced per unit area of sporulating tissue per unit of time (Robert et al., 2002; Dowkiw et al., 2003). It characterizes the flux of spores extruded by the uredinia and is calculated as the ratio between sporulation rate and uredinia size.
• *Mycelium quantity* which gives a proxy of the fungal colonization *in planta* is assessed using qPCR analysis on leaf disks (see below).

Beyond these six quantitative aggressiveness traits, we also measured a morphological trait:

• *Volume of urediniospores* was computed from length and width of urediniospores obtained from image analysis (see below).

We monitored the infections for 13 days, allowing measurements of latent period and infection efficiency. Emergence of uredinia was scored twice a day, at 9 a.m. and at 4 p.m. Infection efficiency was assessed for each leaf disk and was recorded as a binary variable: 1 if the deposited urediniospore produced one uredinia, 0 if not.

At day 13 (end of the monitoring period), leaf disks were harvested. We harvested all infected leaf disks (total: 158 samples out of 576 leaf disks inoculated, mean infection efficiency of 27.4%), giving from 4 to 12 biological replicates per LPI and per plant (mean: 6.58 replicates). The first step of the harvest consisted in separating the urediniospores produced from the leaf disk. Infected leaf disks were placed in 2 mL Eppendorf tubes with 2 mL of Isoton^®^ II isotonic buffered diluent (Beckman Coulter). The tubes were vigorously shaken for 20 s at 4 m s^-1^ on a MP FastPrep^®^-24 homogenizer, in order to release all urediniospores from the uredinia. The second step consisted in measuring the size of the uredinia (or lesion size): photographs of the spore-free uredinia were taken under a stereomicroscope (25× magnification) coupled to a digital camera. Pictures were analyzed using ImageJ version 1.5i with a dedicated script (**Supplementary Note S1**). To measure the number of yellow pixels corresponding to the sporulating area, the Hue Saturation and Brightness was used: pixels with a hue under 37° were counted. Last, infected leaf disks were stored at -80°C and lyophilized before DNA extraction.

Measurements of both sporulation rate and urediniospore morphological parameters were performed on the urediniospore suspensions using an Occhio^®^ Flowcell FC200+ optical morphogranulometer, which allows simultaneous particle counting and image analysis of the particles. Before counting, urediniospore suspensions were vortexed to disperse clusters of urediniospores. For each sample, counting was performed using the following settings: 0.15 mL of priming, 0.85 mL of volume analysis, and 7% of volume sampling (i.e., 7% within 0.85 mL were really analyzed, evenly distributed along the analyzed volume). Two filters parameter values were applied:

1. For urediniospore counting: bluntness between 0.6 and 1; length of ellipse minor axis between 20 and 60 μm; ellipse elongation between 0.18 and 0.79; and ISO eccentricity higher than 0.15,
2. For urediniospore size measurement: bluntness between 0.6 and 1; inertia Feret elongation between 0.25 and 1; ISO eccentricity higher than 0.1; minimum Feret diameter between 13.5 and 70 μm; and Feret diameter length between 23 and 200 μm.

These filters were applied on the raw data to compute sporulation rate expressed in spores per day, length, and width of urediniospores produced by each uredinia. The volume of urediniospores was computed following the formula of the volume of an ellipsoid (Philibert et al., 2011):

$$Spore\;volume=\frac{4}{3}\pi \times \frac{length}{2}\times {\left(\frac{width}{2}\right)}^{2}$$

### DNA Extraction and qPCR Quantitation of *in planta* Mycelium

DNA was extracted from infected poplar leaf disks using the DNeasy96^®^ DNA plant kit (Qiagen). The Frozen Plant Tissue protocol (DNeasy 96 Plant Handbook, June 2015) was followed except that 20 1-mm-diameter glass beads were added to one 3-mm-diameter tungsten carbide bead in the 2 mL Eppendorf tubes. The 158 samples were randomly processed in two DNA extraction plates, which were stored at 4°C until qPCR analysis.

In order to correct for amplification differences among qPCR, three technical replicates were performed.

In order to quantify *M. larici-populina* mycelium *in planta*, quantitative Taqman^®^ PCR was performed using new primers and probe designed for the rDNA ITS (Internal Transcribed Spacer). These primers and probe allowed a significant gain in sensitivity compared to a previously used primer pair, which was designed for a single-copy gene (Guinet et al., 2016). Reagent mix contained 3.1 μL of pure water, 6.5 μL of Mastermix^®^, 0.13 μL of primer ITS-Mlp-F (Primer sequence 5′-3′: TGACTCTTTGTATAAACCATTACCC), 0.13 μL of primer ITS-Mlp-R (TCAAAGTTGCCTTTGAGATACG), and 0.13 μL of probe ITS-Mlp-P (6-FAM-TGCATTGTGGCCCGTCAAAA-BHQ1) per sample. A volume of 3 μL of extracted DNA was added to 10 μL of reagent mix. Standard samples consisted of calibrated quantities of plasmid DNA containing the cloned ITS amplicon. Two series of eight 10-fold dilutions of plasmid DNA (from 1.47 × 10^7^ to 1.47 fg μL^-1^) were included in each qPCR run to set a standard curve. Quantitative PCR runs were performed on a QuantStudio 6 Flex (Life Technologies) machine and consisted in 10 min at 95°C followed by 45 cycles of 15 s at 95°C and 45 s at 62°C. DNA quantity was analyzed using the QuantStudio^TM^ Real-time PCR software (version 1.1).

### Leaf Content Measurements: Total Nitrogen and Total Carbon Contents, Sugars, Phenolic Compounds

All leaf content measurements were conducted on the uninfected leaves, stored at -80°C just after disk excision.

Total nitrogen and total carbon concentrations in uninfected leaves were analyzed using an elemental analyser (Thermo Quest, Type NCS 2500). Measurements, data analysis, data record, and weighing record (Mettler Tolédo MT5 microbalance) were performed using the EAGER 200 software.

Soluble sugars were extracted from 20 mg of dry leaf powder with 1 mL of 70% (v/v) methanol/water for 10 min and then centrifuged at 17,000 *g* for 5 min at 4°C. This step was repeated twice and the resulting supernatants were pooled together. The supernatants, containing soluble sugars, were dried overnight with a vacuum evaporator (Maxi-Dry Plus; Hetomodel DW1, 0-110, Heto-HOLTEN A/S) to eliminate methanol. Dried extracts were rehydrated in 1.5 mL of distilled water, dissolved by sonication and then filtered at 0.2 μm (Acrodisc^®^ Supor^®^ filter; 0.2 μm, Van Waters Rogers). Undiluted aliquots of 20 μL were injected into a high-pressure liquid chromatography system (Dionex^TM^ ICS-5000+ HPLC^TM^) to determine soluble sugar composition. Soluble sugar separation was achieved on a Dionex^TM^ CarboPac^TM^ SA10 IC separation column (6 μm 250 × 4 mm; Thermo Scientific^TM^). The flow rate was maintained at 1 mL min^-1^, and the column temperature at 40°C. Individual carbohydrates were eluted from 0 to 30 min after injection. The sugar peaks were detected by light scattering (Sedex 45 ELSD system, Seder) then identified according to retention time and commercial standards. Sucrose, glucose, and fructose concentrations (% of dry weight) were determined by peak height and standard calibrations.

Phenolics were extracted from 30 mg dry leaf powder using the method described by Royer et al. (2013). Total phenolic content was determined according to the Folin–Ciocalteu method. Briefly, each extract was diluted 1:16 in distilled water, and 20 μL of each diluted extract was distributed in a 96-well plate. Then 100 μL of Folin–Ciocalteu’s reagent (Sigma), diluted 1:10 in distilled water, and 80 μL of 7.5% sodium carbonate (w/v) were added. The microplate was incubated 30 min at 25°C and shaken 5 s every 10 min. Absorbance of the sample was measured with a microplate-reader (SynergyHT, BioTek) at 760 nm. Quantification of total phenolics was performed according to a calibration curve of gallic acid, and the results were expressed as milligram of gallic acid equivalent per gram of leaf dry weight.

In addition to total phenolic content, the concentration of major phenolics was analyzed on ultra-high performance liquid chromatography (U-HPLC) system (Shimadzu) equipped with a photo diode array detector (DAD) and a mass spectrometer. Each sample (3 μl) was separated on a C18 kinetex (100 mm × 2.1 mm) column (Phenomenex). The mobile phase consisted in 0.1% formic acid in ultra-pure water (solvent A) and 0.1% formic acid in methanol (solvent B). The molecules were eluted with a flow rate of 300 μL min^-1^, through a gradient elution from 1 to 50% B for 10 min, then to 99% B in 3 min, which was maintained for three additional minutes. The column was then re-equilibrated to 1% B for 4 min prior to the next run. Mass spectrometry analysis was carried out in ESI positive and negative modes. Quantification was performed by measuring the area under each peak at 280, 320, or 350 nm, depending on the lambda max of each molecule. Due to co-eluting signal at 280 nm, catechin quantification was realized by measuring the area under peak at *m/z* 289, corresponding to the [M-H]– of the molecule. Experimental exact masses and MS fragments were compared to metabolomics databases (Respect^1^; MassBank^2^, DNP^3^) and data available in the literature in order to identify the nature of the metabolites. Sixteen phenolic compounds were identified. According to their structure, these 16 metabolites could be grouped into four phenolic subclasses, which are Flavan-3-ols (catechin, proanthocyanidin dimer), hydroxycinnamic acid esters (chlorogenic acid, caffeoyl shikimate, caffeoyl glucose isomers (×2), coumaroyl glucose isomers (×2)), flavonols (rutin, quercitrin), and salicinoids (salicine, salicortin, acetylsalicortin, homaloside D, tremulacin, and a putative populoside) (**Supplementary Figure S2**).

### Data Analysis

Principal component analyses (PCA) and linear models were used to explore correlations between phenotypic traits. Each independent phenotypic trait was analyzed separately using a generalized linear model evaluating effects of LPI, plant, day of inoculation, and the interaction between LPI and plant. To normalize the error, infection efficiency was analyzed with a binomial error family; latent period and sporulation capacity with a Gamma error family; uredinia size, spore volume, sporulation rate, and mycelium quantity with a Gaussian error family; and uredinia size and sporulation rate were square root transformed. An analysis of variance (ANOVA) with an alpha error <5% was performed on each model. Differences of means were checked with Tukey tests. PCA on leaf characteristics was performed to explore their variations. Spearman correlation coefficients between phenotypic traits and leaf content were computed on mean phenotypic traits per LPI and plant based on 4 to 12 replicates. Spearman correlation coefficients’ associated *p*-values were adjusted with a False-Discovery-Rate correction for multiple tests. All data analyses were performed using R version 3.2.5 (see details in the RMARKDOWN document, **Supplementary Note S2**).

## Results

### Variation in *M. larici-populina* Quantitative Traits

**FIGURE 1:**
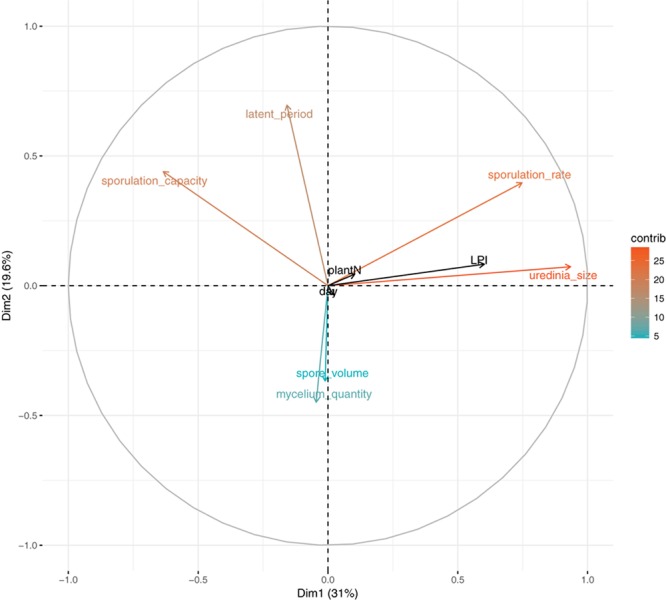
Principal component analysis of fungal phenotypic traits (latent period, uredinia size, sporulation rate, sporulation capacity, spore volume, and mycelium quantity, measured on 4 to 12 replicates per LPI and per plant); LPI, plant and day are supplementary variables. Colors correspond to variables’ contribution to the first and second axes.

To describe the variation in quantitative traits of *M. larici-populina* in this experiment, a PCA was performed on individual trait measurements assessed for each infected leaf disk (latent period, uredinia size, sporulation rate, sporulation capacity, mycelium quantity, and mean volume of produced spores). Infection efficiency cannot be included in the PCA analysis because it is set to one for infected leaf disk. Experimental factors (LPI, plant harvested, and day of inoculation) were considered as supplementary variables. The first principal component (PC1) explained 31% of the total inertia and was related to uredinia size, sporulation rate and to a lesser extent to sporulation capacity (**Figure 1**). PC2 and PC3 accounted for almost 20% and 17% of the total variance, respectively. PC2 was related to latent period which varied independently from uredinia size and sporulation rate, and PC3 was related to spore volume. However, there was large dispersal of individual measures that remained unexplained by experimental factors (**Supplementary Note S2**).

**FIGURE 2:**
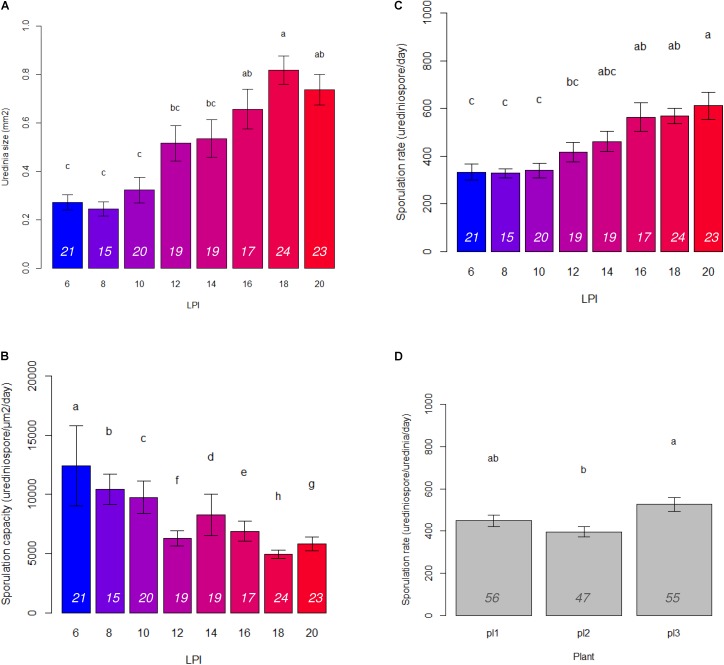
Barplots of means and standard error calculated on three plants and 15 to 24 replicates per LPI (italic numbers at the bottom of the bars) of **(A)** uredinia size, **(B)** sporulation capacity, and **(C)** sporulation rate for each LPI; and **(D)** sporulation rate for each plant. Letters correspond to Tukey test results.

In accordance with PCA analysis, LPI was the main structuring variable in GLM analyses and had a significant effect on uredinia size (*P*_adjusted_ = 1.22 × 10^-21^), sporulation rate (*P*_adjusted_ = 7.67 × 10^-15^), and sporulation capacity (*P*_adjusted_ = 1.40 × 10^-05^) (**Table 1**). Uredinia size and sporulation rate increased with the LPI, while sporulation capacity decreased (**Figure 2**). Uredinia size exhibited a threefold increase (from 0.272 to 0.737 mm^2^) and sporulation rate a twofold increase (from 333 to 611 urediniospores day^-1^) from LPI 6 to 20 (**Figure 2**). Sporulation capacity was reduced more than twofold from LPI 6 to 20 (from 12,420 to 5,800 urediniospores day^-1^ mm^-2^) (**Figure 2**). The sporulation rate was also significantly, although less strongly, explained by the plant used for the experiment (*P*_adjusted_ = 4.63 × 10^-05^). Sporulation rate was significantly higher on plant No. 3 (mean rate = 526 urediniospores day^-1^) and lower on plant No. 2 (mean rate = 396 urediniospores day^-1^) (**Figure 2**), but no interaction was found between plant and LPI (**Table 1**). Other fungal traits varied but not in accordance with LPI, plant and day (**Supplementary Figure S3**).

**Table 1:** Summary of the analysis of variance of each experimental variable in generalized linear models (Chi-square test probability adjusted with a False-Discovery-Rate correction for multiple tests).

Traits	LPI	Plant	LPI:Plant	Day
Infection efficiency	0.459	0.661	0.346	0.165
Latent period	0.720	0.165	0.720	0.661
Uredinia size	**1.22 × 10^-21^**	0.092	0.458	0.346
Spore volume	0.980	0.362	0.720	0.720
Sporulation rate	**7.67 × 10^-15^**	**4.63 × 10^-05^**	0.497	0.090
Sporulation capacity	**1.40 × 10^-05^**	0.379	0.720	0.092
Mycelium quantity	0.866	0.980	0.996	0.720


*P-values < 0.05 are indicated in bold characters.*

We found no significant effect of the day of inoculation on any aggressiveness traits. As there was no difference in fungal traits across days of inoculation, we assumed that the incubation conditions during the pre-inoculation period described above kept the leaf disks in their initial physiological state.

### Variation in Leaf Content Characteristics

**FIGURE 3:**
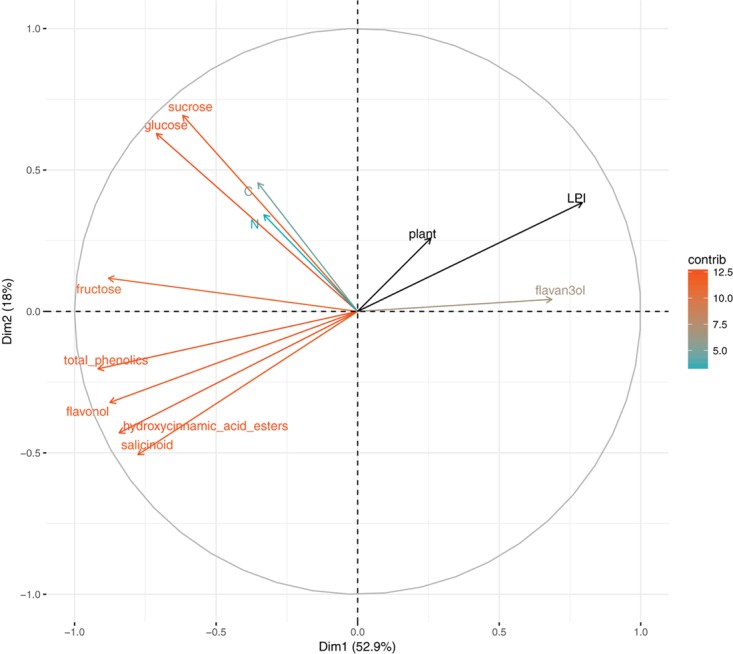
Principal component analysis on leaf chemical content (carbon, nitrogen, glucose, fructose, sucrose, total phenolics, flavan-3-ols, hydroxycinnamic acid esters, flavonols, and salicinoids), LPI and plant are supplementary variables. Colors correspond to variables’ contribution to the first and second axes.

To assess leaf content variations among the range of leaf maturity, a PCA was performed on leaf characteristics (**Figure 3**). Leaf characteristics were measured on one uninfected half-leaf of each plant and each LPI, so only plant and LPI supplementary variables were taken into account. PC1 explained almost 53% of the total inertia and was highly related to sugars (sucrose, fructose, glucose) and phenolic compounds (total phenolics, and all the phenolic subclasses) variations. Explaining 18% and 12% of the inertia, PC2 and PC3 were associated to sucrose and nitrogen, respectively. The total carbon content of leaves did not vary among the range of leaf maturity studied. Leaf maturity, characterized by the content in phenolic compounds, appeared to structure more leaf characteristics than plant did.

### Correlation of Fungal Phenotypic Trait Variation and Leaf Content Characteristics

To assess how fungal and leaf data were correlated, pairwise Spearman’s correlation coefficients and their adjusted *p*-values were calculated.

**FIGURE 4:**
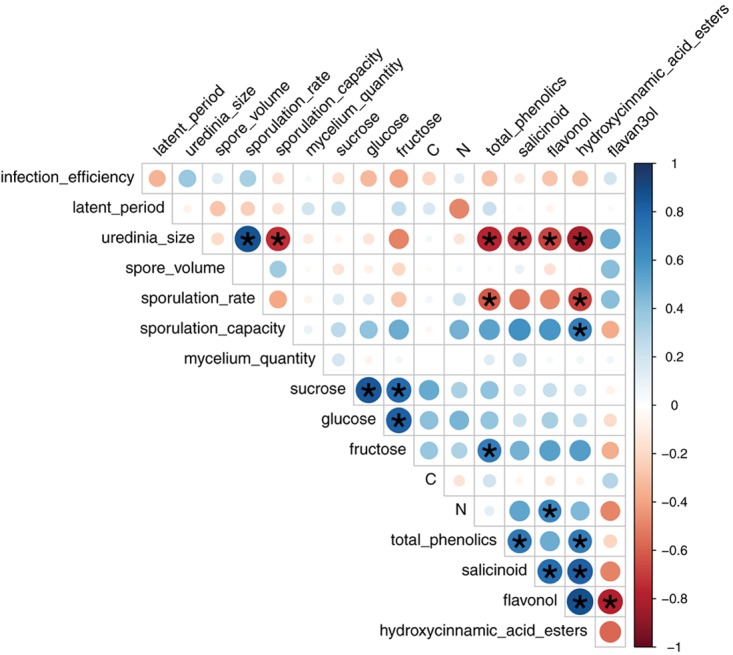
Pairwise Spearman correlation coefficients between leaf chemical content and mean fungal phenotypic traits per LPI and plant based on 4 to 12 replicates. Colors correspond to the sign and dot size to the strength of the correlation. Stars indicate significant correlations according to Student *t*-test adjusted with a False-Discovery-Rate correction for multiple tests.

Spearman’s coefficient for uredinia size and both sporulation rate and sporulation capacity were highly significant (rhô = 0.87 and -0.73, *P*_adjusted_ = 3.37 × 10^-6^ and 4.66 × 10^-4^, respectively) (**Figure 4**), which is consistent with PCA results on individual traits. No other Spearman’s coefficient appeared significant between fungal traits.

**FIGURE 5:**
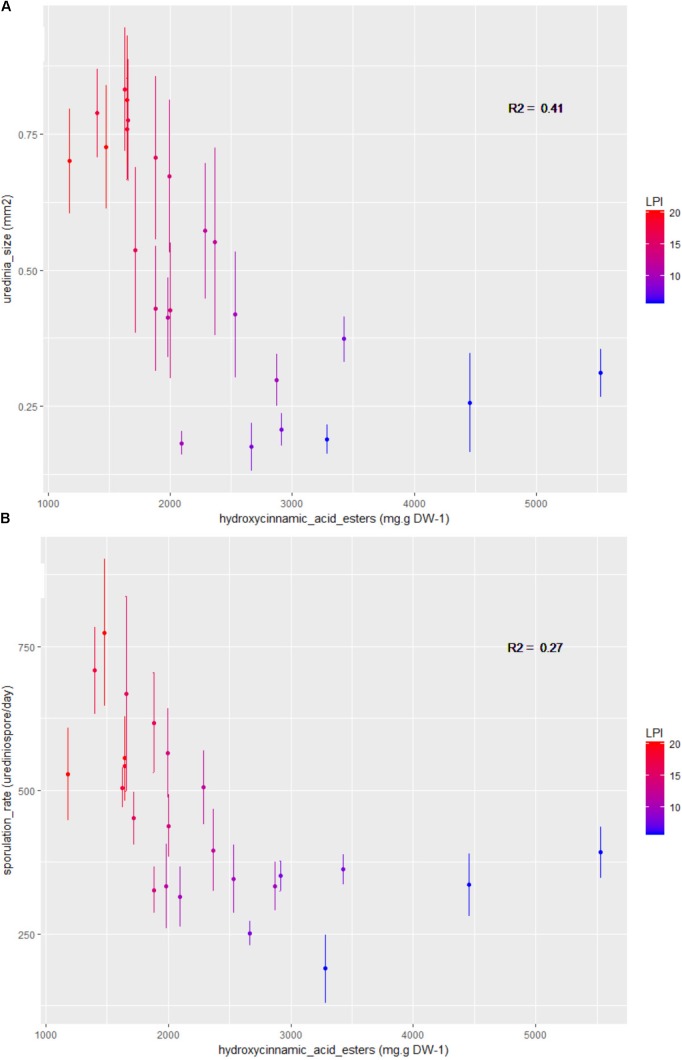
Bivariate plots (LPI and plant mean ± standard error calculated on three plants and 15 to 24 replicates per LPI) showing the variation in **(A)** uredinia size and **(B)** sporulation rate according to the phenolic subclass hydroxycinnamic acid esters content. Colors stand for LPI and R2 gives the coefficients of determination.

Spearman coefficients showed a negative correlation between total phenolic content and uredinia size and sporulation rate (rhô = -0.77 and -0.61, *P*_adjusted_ = 1.62 × 10^-4^ and 1.07 × 10^-2^, respectively), as well as a negative correlation between hydroxycinnamic acid esters content and uredinia size and sporulation rate (rhô = -0.83 and -0.68, *P*_adjusted_ = 2.25 × 10^-5^ and 1.85 × 10^-3^, respectively), and a positive correlation between hydroxycinnamic acid esters content and sporulation capacity (rhô = 0.68, *P*_adjusted_ = 1.85 × 10^-3^) (**Figures 4, 5**). Three of the four phenolic subclasses (salicinoids, hydroxycinnamic acid esters, and flavonols) showed the same trends as total phenolic content, with the exception that the correlation between sporulation rate and sporulation capacity, on one side, and salicinoids and flavonols, on the other side, were not significant (*P*_adjusted_ > 0.05). In accordance with the PCA on leaf characteristics, all sugar contents were correlated with each other. However, no correlation was found between fungal phenotypic traits and sugars, carbon and nitrogen contents. Salicinoids, flavonols, and hydroxycinnamic acid esters were all positively correlated to each other. Flavan-3-ols were, however, negatively correlated to the three other phenolic subclasses, although this was only significant with flavonols.

## Discussion

In this study, we analyzed the variation in seven aggressiveness and morphological traits of the poplar rust fungus when inoculated on poplar leaves of varying maturity levels. A single-spore inoculation technique was used, which enabled us to study trait variance and covariance at the individual lesion level. Applying this method, we aimed at assessing the effect of leaf maturity on aggressiveness traits of *M. larici-populina*. In a second step, we explored correlations between the variation in fungal phenotypic traits and physiological features of the leaves in order to determine whether variation in phenotypic traits was influenced by nutritive or defensive compounds of leaves.

### Effect of Leaf Maturity on Aggressiveness and Morphological Traits

Regarding the variation in aggressiveness traits, our first result was a positive correlation between uredinia size and sporulation rate, whereby both increased with leaf maturity. That is, on more mature leaves, uredinia were wider with a higher production of urediniospores. Some authors found that the breaking down of sporulation rate into uredinia size and sporulation capacity enabled to explain differences in aggressiveness levels: an increase in sporulation rate can either be due to wider uredinia or higher sporulation capacity (flux of urediniospores) (Pariaud et al., 2009b). In our study, we found that the increase in sporulation rate is linked to wider uredinia, but not to higher sporulation capacity. The correlation between uredinia size and sporulation rate has already been identified by several authors, including Dowkiw et al. (2003) on poplar rust, Pei et al. (2002) on willow rust and Robert et al. (2002) on wheat leaf rust. Based on our data, we add to this that sporulation capacity is not necessarily even as assumed by Robert et al. (2002). Indeed, sporulation capacity significantly decreased with leaf maturity, in contrast to uredinia size and sporulation rate. Even if the flux of urediniospores was lower on mature leaves, the amount of urediniospores produced was higher, leading to an increased aggressiveness on mature leaves compared to younger ones.

In this study, we also quantified individual variation in mycelium quantity through qPCR analysis. This trait has received little attention as a component of aggressiveness, mostly for methodological reasons. However, it can serve to measure the relative investment in sporulation vs. mycelium growth, the two main functions to which host extracted resources are allocated (Gilchrist et al., 2006; Yegorov et al., 2017). Notably, the quantity of *in planta* mycelium did not vary along the leaf maturity gradient and was correlated to neither sporulation rate nor lesion size. Hence, lesion size does not equate to mycelium quantity. This makes sense as for biotrophic fungi like rusts, lesion (uredinia) size is only the size of the aperture through which urediniospores are extruded, and may thus not be directly linked to the size of the area within a leaf that is colonized by the mycelium.

Neither leaf maturity, nor the two other experimental factors (plant and day of inoculation) had an effect on latent period, infection efficiency, mycelium quantity and spore volume, even though substantial variances were observed for those traits. The latent period and spore volume varied independently from each other and independently from the main structuring traits (uredinia size and sporulation rate). Variation in latent period and spore volume might have been affected by an unidentified environmental factor. Even though inoculated leaf disks were incubated under controlled conditions of temperature and light, micro-scale variation may have occurred for these crucial parameters influencing fungal development and infection dynamics (Cochrane, 1959; Rotem et al., 1978; Trapero-Casas and Kaiser, 1992; Turechek and Stevenson, 1998; Turechek et al., 2001; Pap et al., 2013). Moreover, the initial position of the disk in the leaf might have influenced infection outcome (Katsuya and Green, 1967; Johnston et al., 2016). Single-spore inoculation is more prone to inter-replicate variance, in contrast to spray-inoculation which buffers micro-environmental variations. Apart from this drawback, this method allowed us to accurately disentangle the variation in each trait and to properly assess putative correlations. In our study, the dissection of the infection process into elementary traits enabled us to assess that the leaf maturity gradient only influences the sporulation function.

### Disentangling Nutritive Limitation vs. Defense Reaction Effects

Two hypotheses were considered to explain variation in uredinia size and sporulation rate. On the one hand, sporulation could be enhanced by a higher nutrient availability in older leaves. On the other hand, higher levels of defense compounds could in contrast prevent fungal development in younger leaves.

Leaf sugars have long been known to be essential to the development of plant pathogenic fungi (Bilgrami and Verma, 1978; Madan and Thind, 1998). Using *Populus deltoides* leaves inoculated with *Melampsora medusae* f. sp. *deltoidae*, Coleman (1986) showed that the maximum level of susceptibility, measured by the number of lesions (uredinia) as a proxy for infection efficiency, is reached when the sugar availability is maximal, i.e., at a LPI of 5. This finding is inconsistent with our study, in which the infection efficiency did not vary with leaf maturity and the two responsive traits (uredinia size and sporulation rate) steadily increased from LPI 6 to 20. The discrepancy between Coleman (1986) experiments and our results may lie in differences in the growth conditions of poplar plants, which may lead to different levels of nutrient availability. The light quality available in growth chambers has been significantly improved during the last decades. In Coleman (1986), the total sugar content decreased from LPI 6 to 9 (the highest LPI measured). In our experiment, even though sugar contents displayed some variance among leaves, there was no consistent pattern along LPI. Our growth conditions may have led to sugar availability sufficient for steady infection success even at higher LPI. We therefore conclude that leaf sugar content may not be linked to leaf susceptibility *per se*.

Leaf nitrogen content also plays a role in fungal growth and sporulation (Bilgrami and Verma, 1978; Madan and Thind, 1998). Bilgrami and Verma (1978) stated that nitrogen sources are used by fungi for both structural and metabolic functions. These functions require the assimilation of nitrogen into amino acids, proteins, and some peptides. For example, Jensen and Munk (1997) and Robert et al. (2004) found that higher nitrogen fertilization enhanced barley powdery mildew and wheat leaf rust sporulation. In our experiment, although there was a substantial variance in leaf nitrogen contents [same order of magnitude as in Robert et al. (2004)], this variation did not significantly affect *M. larici-populina* development. The leaf nitrogen content only slightly correlated with latent period and sporulation capacity (urediniospores produced par day and per area).

Overall, we did not observe consistent variation in nutrient content along the leaf maturity gradient and, moreover, the variation in nutrient content was not correlated with the two responsive traits, uredinia size, and sporulation rate. Consequently, nutrient availability was apparently not the environmental factor best explaining fungal development in our experiment.

By contrast, the defense hypothesis, tested by assessing the constitutive level of phenolics (total and individual) appeared to better explain fungal development related to leaf age. Indeed, the total phenolic content decreased with leaf age and correlated negatively with uredinia size and sporulation rate. Although higher phenolic concentration in younger leaves is common in poplar and other plants (Coleman, 1986; Johnson and Kim, 2005; Royer et al., 2013; Ullah et al., 2017), the correlation between phenolic concentrations and resistance to fungal infection has not been demonstrated. Coleman (1986) and Coleman et al. (1987) did not detect a correlation between leaf phenolic content and the amount of uredinia produced by *M. medusae* on poplar trees. By contrast, in the same pathosystem, a negative correlation was observed between total phenolic content and urediniospore germination efficiency and hyphae length (Johnson and Kim, 2005). Other studies on black currant (*Ribes nigrum*) and willow (*Salix myrsinifolia*) identified negative correlation between the concentrations of phenolic compounds and development traits of rusts (Hakulinen et al., 1999; Vagiri et al., 2017). By analyzing specific phenolics from poplar leaves, we were able to precisely identify groups of phenolic compounds that correlate with poplar resistance to rust. Among the four phenolic subclasses (from a total of 16 compounds), three subclasses (hydroxycinnamic acid esters, flavonols, and salicinoids) showed the same effect as total phenolic contents and correlated negatively with uredinia size and sporulation rate. The hydroxycinnamic acid ester chlorogenic acid has been shown to correlate to rust resistance in black currant (Vagiri et al., 2017). In addition, several studies highlighted the role of leaf surface flavonoids in the resistance to rust in poplar and birch (Shain and Miller, 1982; Valkama et al., 2005). In our study, we did not address the leaf location (inside or on the surface) of the two flavonols, rutin and quercetrin, however, the fact that they negatively correlate with rust development is in agreement with these previous studies. The only phenolic subclass that did not correlate with resistance to rust was flavan-3-ol which integrates catechin and proanthocyanidin dimers. A role of this phenolic subclass – especially catechin – in resistance to fungal pathogens, including rust fungi, has been proposed in several studies (Brignolas et al., 1998; Hakulinen et al., 1999; Vagiri et al., 2017). Moreover, a recent study on the *M. larici-populina* – *Populus nigra* pathosystem demonstrated that catechin is an effective chemical defense compound against rust (Ullah et al., 2017). This demonstration was based on the fact that (i) catechin is strongly induced upon rust infection, (ii) catechin exhibit a strong antifungal activity against *M. larici-populina* at *in planta* concentration, and (iii) poplar engineered to accumulate high or low levels of catechin were more resistant or more susceptible to rust, respectively. In our study, the concentrations of flavan-3-ols increased with leaf age. The same trend was observed in the study by Ullah et al. (2017) (see **Supplementary Figure S2**). The absence of a correlation between flavan-3-ol concentrations and rust resistance does not indicate that flavan-3-ols are not important factors in leaf rust resistance. Indeed, the compounds were assayed in uninfected leaves; hence reflect constitutive (i.e., non-induced) defense levels. In addition, the concentrations measured in our experiment (from 0.2 to 0.5 mg.g DW^-1^) were below the concentrations proven to efficiently impair rust development on black poplar leaves (Ullah et al., 2017).

## Conclusion

*Melampsora larici-populina* development after single-spore inoculation onto poplar leaf disks was more constrained by the level of plant constitutive defenses than limited by nutrient availability. For the tested strain, uredinia size and sporulation rate, both components of the sporulation function, were the only traits responsive to reaction norms along a gradient of leaf maturity. This experiment – conducted on a single fungal isolate and on a single plant genotype – can nonetheless serve as a pilot study. We report the correlation of a fungal fitness component (sporulation) to an environmental characteristic imposed by the leaf i.e., the constitutive level of phenolic content. In the future, it would be worthwhile to assess the generality of this finding with more fungal isolates and plant genotypes and decipher further the interplay between constitutive and induced defense compound levels. From a broader perspective, it would be interesting to determine the plasticity and heritability of sporulation-related traits to better assess how the complexity of leaf environment shapes the evolutionary trajectory of a plant pathogen.

## Author Contributions

FH, PF, and BF designed the study. AM conducted the experiment and analyses. MP developed the single-spore inoculation and trait measurement methods. AA developed the qPCR method based on new primers and probe for *Melampsora larici-populina* in the rDNA ITS (Internal Transcribed Spacer) designed by CG and A-LB. RL conducted leaf phenolics analyses. AM and FH wrote the first draft of the manuscript. PF, MP, and RL contributed to the manuscript. All the authors approved the final version of the manuscript.

## Conflict of Interest Statement

The authors declare that the research was conducted in the absence of any commercial or financial relationships that could be construed as a potential conflict of interest.

## Footnotes

1 http://spectra.psc.riken.jp/

2 http://massbank.jp/

3 http://dnp.chemnetbase.com/
