# Supplementary material for: Defense Compounds Rather Than Nutrient Availability Shape Aggressiveness Trait Variation Along a Leaf Maturity Gradient in a Biotrophic Plant Pathogen
Source: Front Plant Sci. 2018 Sep 28;9:1396. doi: 10.3389/fpls.2018.01396 (PMC6172983; doi:10.3389/fpls.2018.01396)
Supplement: Supplementary file 1 [file Data_Sheet_1.pdf]

Supplementary Material

Defense compounds rather than nutrient availability shape aggressiveness trait variation along a leaf maturity gradient in a biotrophic plant pathogen

Agathe Maupetit, Romain Larbat, Michaël Pernaci, Axelle Andrieux, Cécile Guinet, Anne-Laure Boutigny, Bénédicte Fabre, Pascal Frey and Fabien Halkett

Frontiers in Plant Science (2018) 9:1396. doi: 10.3389/fpls.2018.01396

Table of Content:

Figure S1: **Page 2**

Experimental design for the 24 cell culture plates. Numbers correspond to the 3 eight LPIs and colours to the three plants.

Figure S2: **Page 4**

Principal component analysis on leaf chemical content (carbon, nitrogen, glucose, fructose, sucrose, salicin, total phenolics, Flavan-3-ols (Catechin, Proanthocyanidin dimers), Hydroxycinnamic acid esters (chlorogenic acid, caffeoyl shikimate, caffeoyl glucose isomer1 and isomer2, coumaroyl glucose isomer1 and isomer2), Flavonols (rutin, quercitrin) and Salicinoids (Salicine, salicortin, acetylsalicortin, homaloside D, tremulacin and a putative populoside), LPI and plant are supplementary variables. Colors correspond to variables' contribution to the first and second axes.

Figure S3: **Page 5**

Barplots of trait means and standard error, (i) across LPI: (A) infection efficiency, (D) latent period, (G) mycelium quantity, and (J) spore volume; (ii) for each inoculation day and each plant: (B and C) infection efficiency, (E and F) latent period, (H and I) mycelium quantity, (K and L) spore volume, (M and N) uredinia size, (O and P) sporulation capacity, and (Q) sporulation rate (for each day only).

Note S1: **Page 8**

ImageJ script for uredinia size analysis

Note S2: **Page 10**

Script and outputs of statistical analyses using R (RMarkdown document)

Figure S1: Experimental design for the 24 cell culture plates. Numbers correspond to the eight LPIs and colours to the three plants.

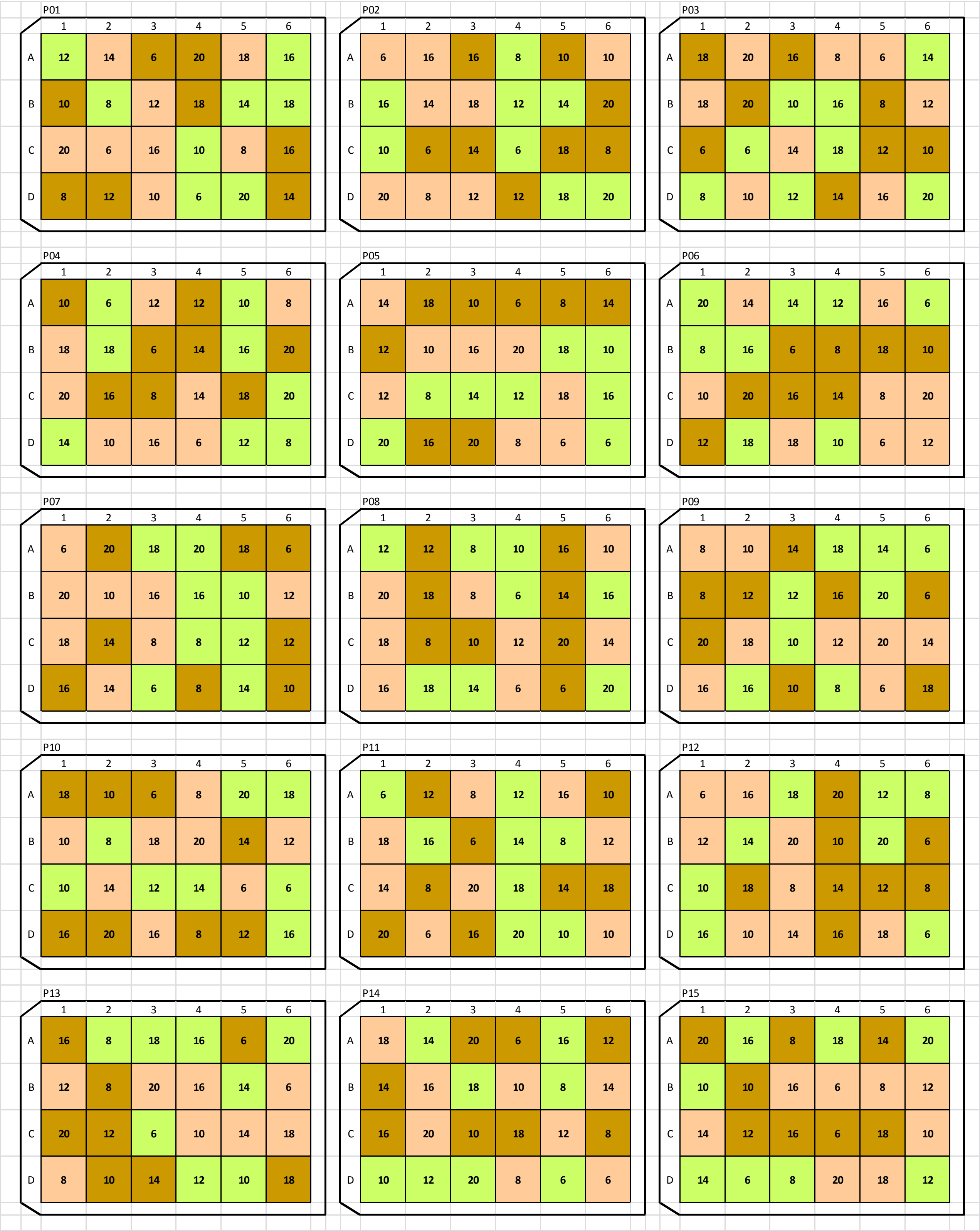

P19

	1	2	3	4	5	6
A	20	14	10	20	20	14
B	12	18	16	12	10	6
C	6	6	12	8	8	18
D	16	14	8	10	16	18

P20

	1	2	3	4	5	6
A	16	8	20	16	6	6
B	10	20	8	14	6	16
C	14	10	12	14	18	8
D	18	12	20	12	10	18

P21

	1	2	3	4	5	6
A	6	18	18	14	12	20
B	6	12	6	20	14	10
C	8	14	16	16	12	16
D	10	10	20	8	8	18

P22

	1	2	3	4	5	6
A	16	14	10	18	6	12
B	20	10	18	6	16	20
C	12	18	10	16	8	6
D	14	8	8	14	20	12

P23

	1	2	3	4	5	6
A	12	12	6	16	10	8
B	10	20	6	20	8	14
C	12	16	6	14	18	14
D	10	16	18	20	8	18

P24

	1	2	3	4	5	6
A	8	6	12	20	18	12
B	16	18	10	16	20	16
C	8	12	20	14	10	10
D	18	14	6	14	8	6

Figure S2: Principal component analysis on leaf chemical content (carbon, nitrogen, glucose, fructose, sucrose, salicin, total phenolics, Flavan-3-ols (Catechin, Proanthocyanidin dimers), Hydroxycinnamic acid esters (chlorogenic acid, caffeoyl shikimate, caffeoyl glucose isomer1 and isomer2, coumaroyl glucose isomer1 and isomer2), Flavonols (rutin, quercitrin) and Salicinoids (Salicine, salicortin, acetylsalicortin, homaloside D, tremulacin and a putative populoside), LPI and plant are supplementary variables. Colors correspond to variables' contribution to the first and second axes.

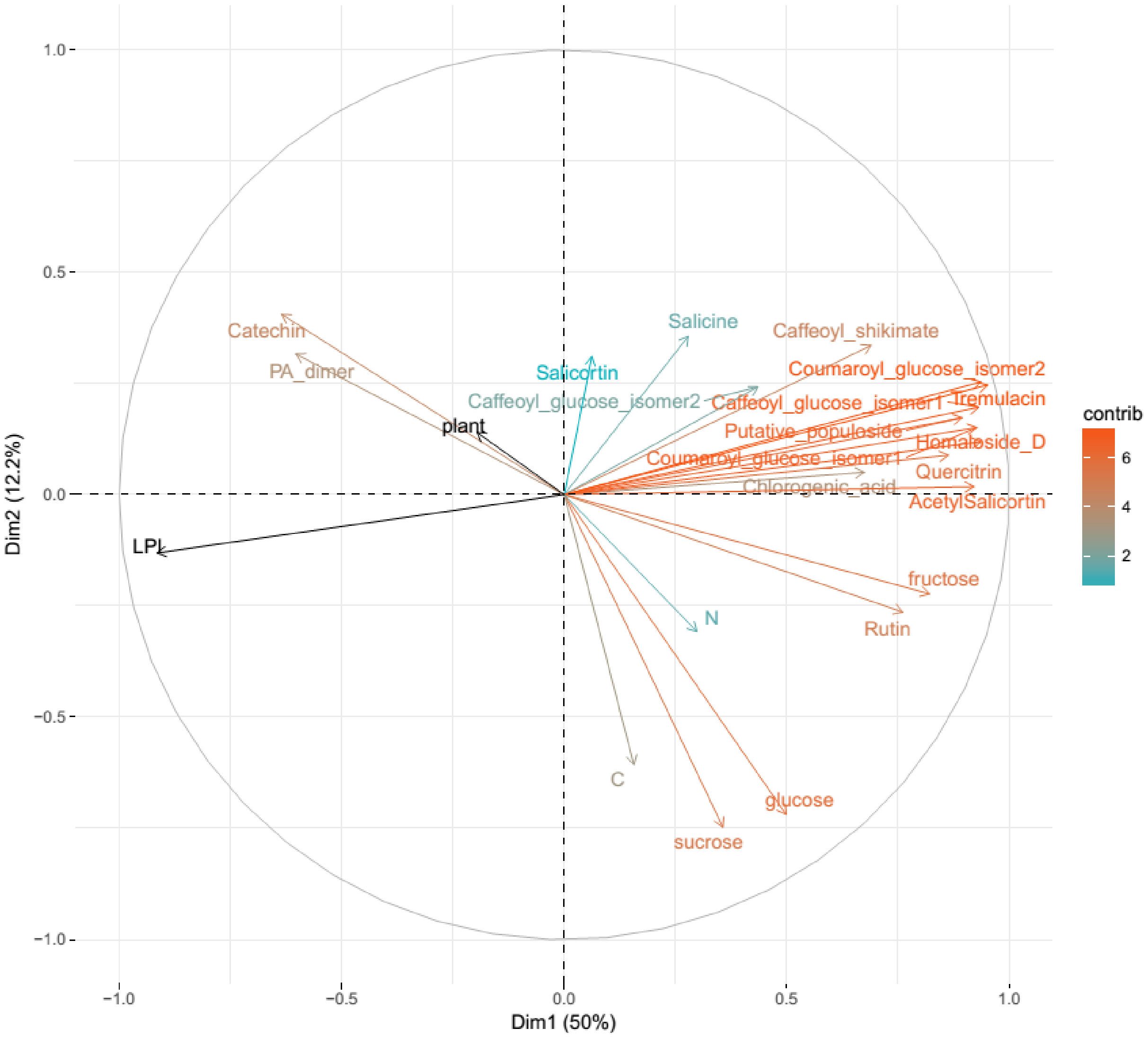

Figure S3: Barplots of trait means and standard error, (i) across LPI: **(A)** infection efficiency, **(D)** latent period, **(G)** mycelium quantity, and **(J)** spore volume; (ii) for each inoculation day and each plant: **(B and C)** infection efficiency, **(E and F)** latent period, **(H and I)** mycelium quantity, **(K and L)** spore volume, **(M and N)** uredinia size, **(O and P)** sporulation capacity, and **(Q)** sporulation rate (for each day only).

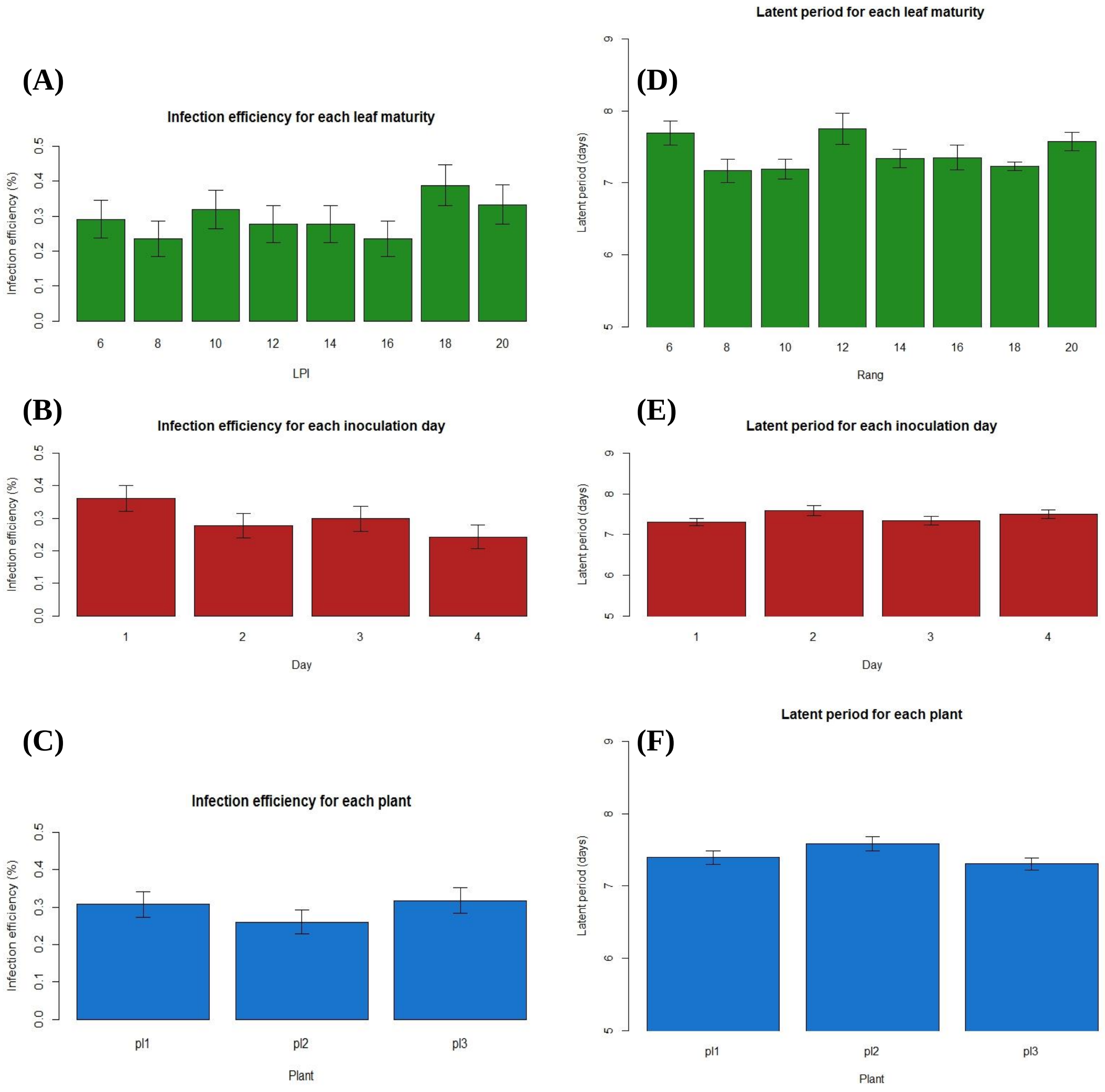

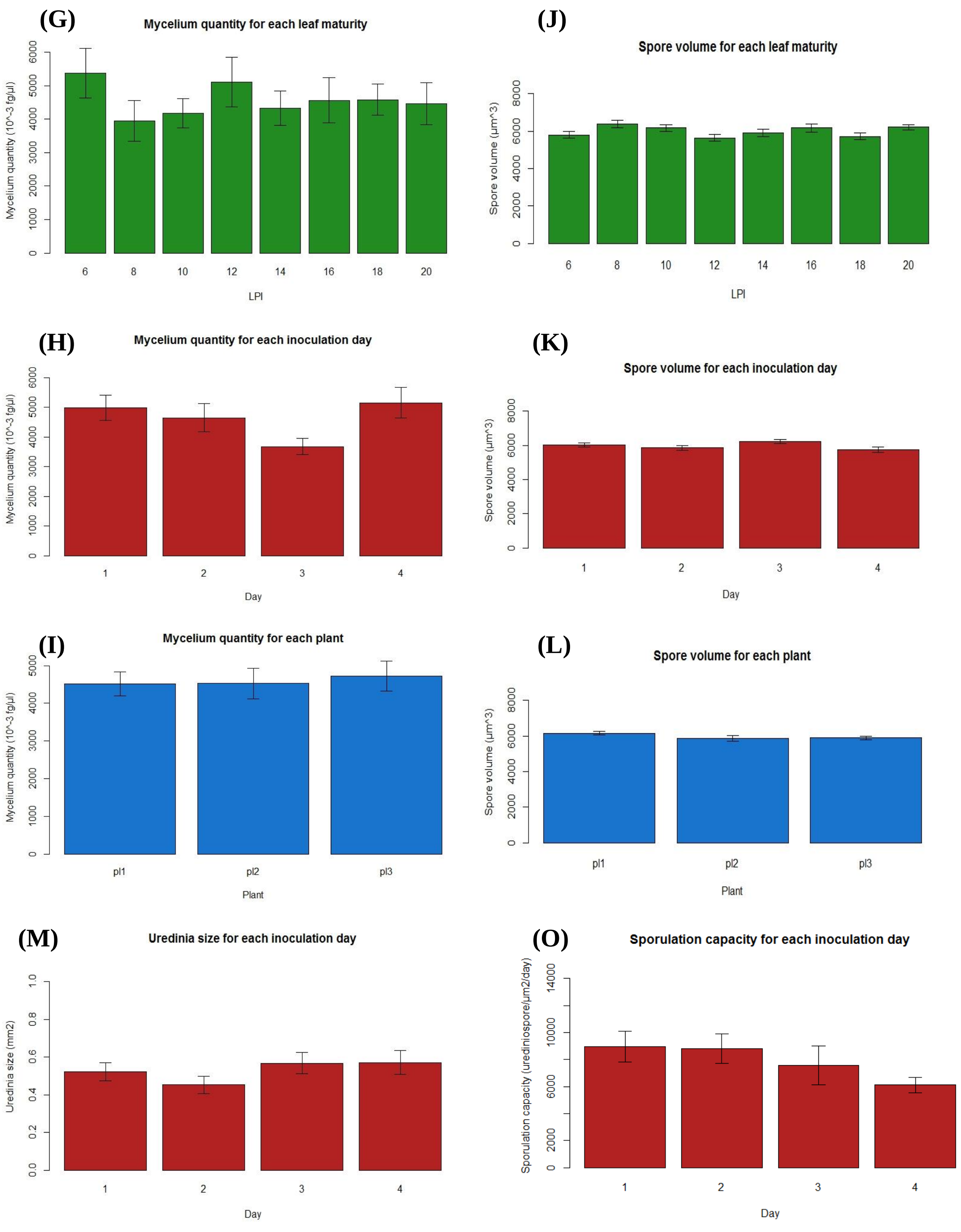

(G)
Mycelium quantity for each leaf maturity
Mycelium quantity (10^-3 fg/µl)
LPI
(J)
Spore volume for each leaf maturity
Spore volume (µm^3)
LPI
(H)
Mycelium quantity for each inoculation day
Mycelium quantity (10^-3 fg/µl)
Day
(K)
Spore volume for each inoculation day
Spore volume (µm^3)
Day
(I)
Mycelium quantity for each plant
Mycelium quantity (10^-3 fg/µl)
Plant
(L)
Spore volume for each plant
Spore volume (µm^3)
Plant
(M)
Uredinia size for each inoculation day
Uredinia size (mm2)
Day
(O)
Sporulation capacity for each inoculation day
Sporulation capacity (urediniospore/µm2/day)
Day

(N)

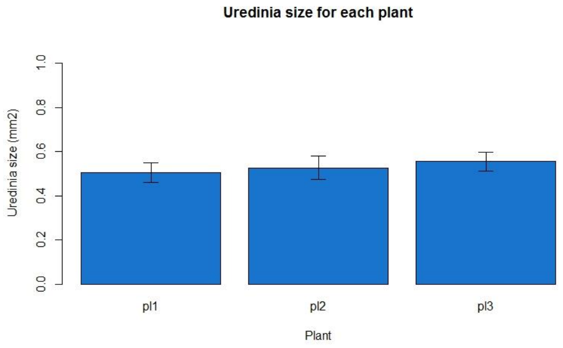

Uredinia size for each plant
Uredinia size (mm2)
0.0
0.2
0.4
0.6
0.8
1.0
pl1
pl2
pl3
Plant

(P)

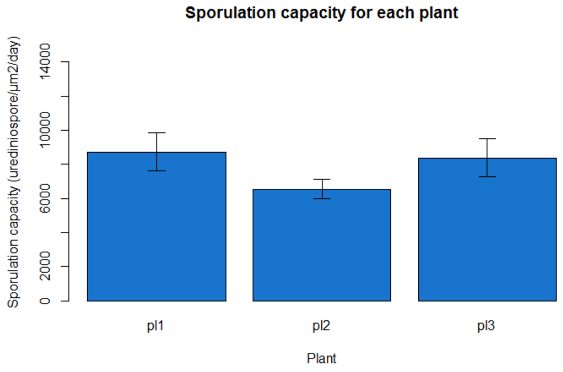

Sporulation capacity for each plant
Sporulation capacity (urediniospore/µm2/day)
0
2000
6000
10000
14000
pl1
pl2
pl3
Plant

(Q)

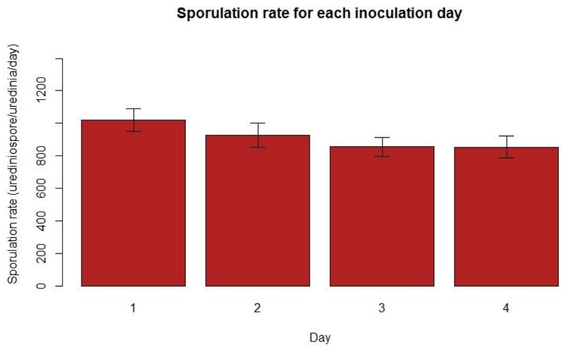

Sporulation rate for each inoculation day
Sporulation rate (urediniospore/uredinia/day)
0
200
400
600
800
1200
1
2
3
4
Day

Note S1: ImageJ script for uredinia size analysis

```
// Area calculation for all pictures in the directory
input = "Z:\\Rangf\\Rgf_photoU\\";
list = getFileList(input);

for (p = 0; p < list.length; p++) {
open(list[p]) ;
// Color Thresholder 1.49v
// Autogenerated macro, single images only!
	min=newArray(3);
	max=newArray(3);
	filter=newArray(3);
	a=getTitle();
	run("HSB Stack");
	run("Convert Stack to Images");
	selectWindow("Hue");
	rename("0");
	selectWindow("Saturation");
	rename("1");
	selectWindow("Brightness");
	rename("2");
	min[0]=0;
	max[0]=37;
	filter[0]="pass";
	min[1]=0;
	max[1]=255;
	filter[1]="pass";
	min[2]=0;
	max[2]=255;
	filter[2]="pass";
	for (i=0;i<3;i++){
	  selectWindow(""+i);
	  setThreshold(min[i], max[i]);
	  run("Convert to Mask");
	  if (filter[i]=="stop")  run("Invert");
	}
	imageCalculator("AND create", "0","1");
	imageCalculator("AND create", "Result of 0","2");
	for (i=0;i<3;i++){
	  selectWindow(""+i);
	  close();
	}
	selectWindow("Result of 0");
	close();
```

```
        selectWindow("Result of Result of 0");
        rename(a);
        // Colour Thresholding-------------
        run("8-bit");
        run("Close-");
        run("Fill Holes");
        run("Set Scale...", "distance=392 known=1000 pixel=1 unit=microm");
        run("Analyze Particles...", "size=19000.00-Infinity display exclude");
        run("Close All");
}
```

Note S2: Script and outputs of statistical analyses using R (RMarkdown document)

Analysis: Leaf maturity

```
knitr::opts_chunk$set(echo = T, include = T, message = T, error = T, warnin
g = F)
setwd("C:/OneDrive Entreprise 1/Rangf")
rm(list=ls())

library(sciplot) # bargraph
library(car) # Anova
library(agricolae) # HSD.test
library(Hmisc) # test of correlations
library(lme4) # (g)lmer
library(ggplot2) # plots
library(factoextra) # plot PCA
library(ade4) # PCA
library(plotrix) # locator in plot
library(corrplot) # correlation matrix representation
library(knitr) # table
```

Dataset

```
results <- read.table('M:/Rangf/Rgf_results.txt', header =T, dec=".")
results <- results[,c(2:7,9:12)]
names(results) <- c( "LPI", "plant", "day", "infection_efficiency", "latent
_period", "uredinia_size", "spore_volume", "sporulation_rate", "sporulation
_capacity", "mycelium_quantity")

# Add LPI-plant id
results$leaf_id <- paste("lpi", results$LPI, results$plant, sep="")

# Add sugars and phenolics contents
sugar <- read.table('M:/Rangf/Rgf_sugar.txt', header=T)
sugar$leaf_id <- paste("lpi", sugar$LPI, "pl",sugar$plant, sep="")
phenolics <- read.table('M:/Rangf/phenolicsRomain.txt', header=T)
phenolics$leaf_id <- paste("lpi", phenolics$LPI, "pl",phenolics$plant, sep=
"")

results$sugars <- NA
results$sucrose <- NA
results$salicin <- NA
results$glucose <- NA
results$fructose <- NA
results$Chlorogenic_acid <- NA
results$PA_dimer <- NA
```

```
results$Salicine <- NA
results$Caffeoyl_glucose_isomer1 <- NA
results$Catechin <- NA
results$Coumaroyl_glucose_isomer1 <- NA
results$Caffeoyl_glucose_isomer2 <- NA
results$Coumaroyl_glucose_isomer2 <- NA
results$Caffeoyl_shikimate <- NA
results$Salicortin <- NA
results$Rutin <- NA
results$AcetylSalicortin <- NA
results$Quercitrin <- NA
results$Homaloside_D <- NA
results$Putative_populoside <- NA
results$Tremulacin <- NA
results$flavan3ol <- NA
results$hydroxycinnamic_acid_esters <- NA
results$flavonol <- NA
results$salicinoid <- NA

i=1 # line of results
for (li in 1:nrow(results)){
  t=1 # line of sugar
  for (t in 1:nrow(sugar)){
    if (results[i,"leaf_id"] == sugar[t,"leaf_id"]){
      results[i,"sugars"] = sugar[t,"total.sugar"];
      results[i,"sucrose"] = sugar[t,"sucrose"];
      results[i,"salicin"] = sugar[t,"salicin"];
      results[i,"glucose"] = sugar[t,"glucose"];
      results[i,"fructose"] = sugar[t,"fructose"];
      results[i,"Chlorogenic_acid"] = phenolics[t,"Chlorogenic_acid"];
      results[i,"PA_dimer"] = phenolics[t,"PA_dimer"];
      results[i,"Salicine"] = phenolics[t,"Salicine"];
      results[i,"Caffeoyl_glucose_isomer1"] = phenolics[t,"Caffeoyl_glucose
_isomer1"];
      results[i,"Catechin"] = phenolics[t,"Catechin"];
      results[i,"Coumaroyl_glucose_isomer1"] = phenolics[t,"Coumaroyl_gluco
se_isomer1"];
      results[i,"Caffeoyl_glucose_isomer2"] = phenolics[t,"Caffeoyl_glucose
_isomer2"];
      results[i,"Coumaroyl_glucose_isomer2"] = phenolics[t,"Coumaroyl_gluco
se_isomer2"];
      results[i,"Caffeoyl_shikimate"] = phenolics[t,"Caffeoyl_shikimate"];
      results[i,"Salicortin"] = phenolics[t,"Salicortin"];
      results[i,"Rutin"] = phenolics[t,"Rutin"];
      results[i,"AcetylSalicortin"] = phenolics[t,"AcetylSalicortin"];
      results[i,"Quercitrin"] = phenolics[t,"Quercitrin"];
      results[i,"Homaloside_D"] = phenolics[t,"Homaloside_D"];
```

```
      results[i,"Putative_populoside"] = phenolics[t,"Putative_populoside"]
;
      results[i,"Tremulacin"] = phenolics[t,"Tremulacin"];
      results[i,"flavan3ol"] = phenolics[t,"flavan3ol"];
      results[i,"hydroxycinnamic_acid_esters"] = phenolics[t,"hydroxycinnam
ic_acid_esters"];
      results[i,"flavonol"] = phenolics[t,"flavonol"];
      results[i,"salicinoid"] = phenolics[t,"salicinoid"];}
    else{t=t+1}
  }
  i=i+1
}

# Add C and N content
CN <- read.table('C:/OneDrive Entreprise 1/Rangf/Rgf_CN.txt', header=T)
CN$leaf_id <- paste("lpi", CN$LPI, "pl",CN$plant, sep="")
results$C <- NA
results$N <- NA
i=1 # line of results
for (li in 1:nrow(results)){
  t=1 # line of CN
  for (t in 1:nrow(CN)){
    if (results[i,"leaf_id"] == CN[t,"leaf_id"])
    {results[i,"C"] = CN[t,"C"];
    results[i,"N"] = CN[t,"N"]}
    else{t=t+1}
  }
  i=i+1
}

# Add total_phenolics content
phenol <- read.table('Rgf_phenol.txt', header=T)
phenol$leaf_id <- paste("lpi", phenol$LPI, "pl", phenol$plant, sep="")
results$total_phenolics <- NA
i=1 # line of results
for (li in 1:nrow(results)){
  t=1 # line of phenol
  for (t in 1:nrow(phenol)){
    if (results[i,"leaf_id"] == phenol[t,"leaf_id"]){results[i,"total_pheno
lics"] = phenol[t,"polyphenols"]}
    else{t=t+1}
  }
  i=i+1
}

# Create dataset without NA
resna <- na.omit(results)
```

```r
print(results[1:5,])
```

```
##   LPI plant day infection_efficiency latent_period uredinia_size
## 1  12   pl2   1                    0            NA            NA
## 2  14   pl3   1                    0            NA            NA
## 3   6   pl1   1                    0            NA            NA
## 4  20   pl1   1                    0            NA            NA
## 5  18   pl3   1                    0            NA            NA
##   spore_volume sporulation_rate sporulation_capacity mycelium_quantity
## 1           NA               NA                   NA                NA
## 2           NA               NA                   NA                NA
## 3           NA               NA                   NA                NA
## 4           NA               NA                   NA                NA
## 5           NA               NA                   NA                NA
##    leaf_id  sugars sucrose salicin glucose fructose Chlorogenic_acid
## 1 lpi12pl2 11.5433  5.9248  3.3874  1.7929   0.4382         293.3551
## 2 lpi14pl3 12.4972  6.6359  2.9483  2.5042   0.4088         235.6265
## 3  lpi6pl1 19.5985  8.4850  7.0431  3.2729   0.7975         329.5043
## 4 lpi20pl1  9.9788  5.5914  2.2550  1.7540   0.3784         144.9384
## 5 lpi18pl3 13.3956  7.1842  2.4821  3.2714   0.4579         208.6286
##    PA_dimer Salicine Caffeoyl_glucose_isomer1 Catechin
## 1 102.09041 225.5841                225.11115 194.6124
## 2  83.63304 212.9060                243.87910 232.0630
## 3 105.26757 402.4872                828.99821 258.8848
## 4 169.07114 192.3394                 64.12952 491.6920
## 5  60.43040 217.1388                114.77916 230.8109
##   Coumaroyl_glucose_isomer1 Caffeoyl_glucose_isomer2
## 1                 374.29476                 390.6262
## 2                 434.34332                 383.9139
## 3                1606.89498                 605.2932
## 4                  64.94667                 168.3602
## 5                 207.05440                 359.4917
##   Coumaroyl_glucose_isomer2 Caffeoyl_shikimate Salicortin    Rutin
## 1                  170.5567           527.2811   2183.894 3863.224
## 2                  177.3747           519.3555   2286.413 5446.480
## 3                  485.8900           596.7956   2515.940 3922.821
## 4                  175.0707           560.6161   7817.305 2379.541
## 5                  103.3844           406.3848   1749.402 3909.784
##   AcetylSalicortin Quercitrin Homaloside_D Putative_populoside Tremulaci
n
## 1         1954.644   6432.348     3051.530            2175.010   487.539
6
## 2         2525.289   7793.757     3043.833            2594.959   555.302
2
## 3         3326.623   9308.485     4290.555            5048.813  1429.551
3
```

```
## 4         1294.262   4549.841     2361.481            1457.454   284.545
8
## 5         1592.809   4713.801     2596.871            1408.428   304.051
5
##   flavan3ol hydroxycinnamic_acid_esters  flavonol salicinoid        C
## 1  296.7028                    1981.225 10295.572  10078.202 43.87680
## 2  315.6960                    1994.493 13240.237  11218.703 43.60076
## 3  364.1524                    4453.376 13231.306  17013.969 43.71679
## 4  660.7631                    1178.062  6929.382  13407.387 43.72755
## 5  291.2413                    1399.723  8623.585   7868.701 43.40789
##          N total_phenolics
## 1 1.746716            41.1
## 2 2.334496            37.7
## 3 2.139641            54.5
## 4 1.862166            39.3
## 5 2.237561            32.3
```

```
summary(results)
```

```
##       LPI        plant          day       infection_efficiency
##  Min.   : 6.0   pl1:192   Min.   :1.00   Min.   :0.0000
##  1st Qu.: 9.5   pl2:192   1st Qu.:1.75   1st Qu.:0.0000
##  Median :13.0   pl3:192   Median :2.50   Median :0.0000
##  Mean   :13.0             Mean   :2.50   Mean   :0.2951
##  3rd Qu.:16.5             3rd Qu.:3.25   3rd Qu.:1.0000
##  Max.   :20.0             Max.   :4.00   Max.   :1.0000
##
##  latent_period    uredinia_size     spore_volume  sporulation_rate
##  Min.   : 6.000   Min.   :0.0266   Min.   :3326   Min.   :  98.0
##  1st Qu.: 7.000   1st Qu.:0.2431   1st Qu.:5522   1st Qu.: 309.8
##  Median : 7.250   Median :0.4429   Median :6019   Median : 413.4
##  Mean   : 7.413   Mean   :0.5213   Mean   :5988   Mean   : 458.3
##  3rd Qu.: 7.500   3rd Qu.:0.7383   3rd Qu.:6497   3rd Qu.: 608.5
##  Max.   :10.000   Max.   :1.6141   Max.   :9109   Max.   :1241.1
##  NA's   :410      NA's   :410      NA's   :417    NA's   :417
##  sporulation_capacity mycelium_quantity   leaf_id
##  Min.   : 2092        Min.   :  613.1   Length:576
##  1st Qu.: 4322        1st Qu.: 2692.8   Class :character
##  Median : 6255        Median : 4309.4   Mode  :character
##  Mean   : 7945        Mean   : 4607.5
##  3rd Qu.: 9110        3rd Qu.: 5692.0
##  Max.   :64429        Max.   :14388.9
##  NA's   :417          NA's   :411
##      sugars          sucrose          salicin          glucose
##  Min.   : 3.232   Min.   :1.586   Min.   :0.8027   Min.   :0.4798
##  1st Qu.: 8.580   1st Qu.:3.889   1st Qu.:2.1578   1st Qu.:1.6700
##  Median :11.912   Median :5.914   Median :2.6902   Median :2.2866
##  Mean   :11.135   Mean   :5.511   Mean   :2.9631   Mean   :2.1963
```

```
##  3rd Qu.:14.130   3rd Qu.:7.188   3rd Qu.:3.8746   3rd Qu.:2.9285  
##  Max.   :19.599   Max.   :8.485   Max.   :7.0431   Max.   :3.4722  
##                                                                    
##     fructose      Chlorogenic_acid    PA_dimer         Salicine    
##  Min.   :0.3273   Min.   :144.9    Min.   : 36.02   Min.   :182.2  
##  1st Qu.:0.3782   1st Qu.:235.0    1st Qu.: 79.25   1st Qu.:193.1  
##  Median :0.4401   Median :280.4    Median :104.60   Median :217.0  
##  Mean   :0.4644   Mean   :279.1    Mean   :106.59   Mean   :240.9  
##  3rd Qu.:0.5192   3rd Qu.:322.6    3rd Qu.:130.10   3rd Qu.:246.2  
##  Max.   :0.7975   Max.   :427.5    Max.   :212.45   Max.   :491.6  
##                                                                    
##  Caffeoyl_glucose_isomer1    Catechin     Coumaroyl_glucose_isomer1
##  Min.   :  64.13          Min.   :171.3   Min.   :  64.95          
##  1st Qu.: 141.72          1st Qu.:223.3   1st Qu.: 223.32          
##  Median : 234.28          Median :252.5   Median : 385.07          
##  Mean   : 314.29          Mean   :271.4   Mean   : 598.48          
##  3rd Qu.: 370.11          3rd Qu.:311.2   3rd Qu.: 824.29          
##  Max.   :1131.92          Max.   :491.7   Max.   :2430.21          
##                                                                    
##  Caffeoyl_glucose_isomer2 Coumaroyl_glucose_isomer2 Caffeoyl_shikimate
##  Min.   :168.4            Min.   :101.7             Min.   :406.4     
##  1st Qu.:388.9            1st Qu.:116.9             1st Qu.:474.1     
##  Median :442.3            Median :175.1             Median :510.3     
##  Mean   :432.7            Mean   :213.7             Mean   :516.2     
##  3rd Qu.:469.2            3rd Qu.:275.5             3rd Qu.:549.9     
##  Max.   :605.3            Max.   :485.9             Max.   :658.6     
##                                                                       
##    Salicortin       Rutin      AcetylSalicortin   Quercitrin   
##  Min.   :1576   Min.   :2380   Min.   :1250     Min.   : 4550  
##  1st Qu.:1812   1st Qu.:2984   1st Qu.:1575     1st Qu.: 5032  
##  Median :2253   Median :3883   Median :1996     Median : 6652  
##  Mean   :2487   Mean   :4177   Mean   :2289     Mean   : 6830  
##  3rd Qu.:2527   3rd Qu.:4510   3rd Qu.:3096     3rd Qu.: 8358  
##  Max.   :7817   Max.   :8637   Max.   :4391     Max.   :11105  
##                                                                
##   Homaloside_D  Putative_populoside   Tremulacin       flavan3ol    
##  Min.   :2361   Min.   :1408        Min.   : 183.7   Min.   :209.3  
##  1st Qu.:2643   1st Qu.:1666        1st Qu.: 331.9   1st Qu.:291.9  
##  Median :3048   Median :2217        Median : 506.3   Median :361.8  
##  Mean   :3159   Mean   :2674        Mean   : 612.9   Mean   :378.0  
##  3rd Qu.:3502   3rd Qu.:3319        3rd Qu.: 736.5   3rd Qu.:434.0  
##  Max.   :4348   Max.   :5049        Max.   :1450.8   Max.   :660.8  
##                                                                     
##  hydroxycinnamic_acid_esters    flavonol       salicinoid   
##  Min.   :1178                Min.   : 6929   Min.   : 7425  
##  1st Qu.:1653                1st Qu.: 8502   1st Qu.: 8298  
##  Median :1998                Median :10860   Median :10824  
```

```
##  Mean   :2354                Mean   :11007   Mean   :11462
##  3rd Qu.:2718                3rd Qu.:13234   3rd Qu.:13403
##  Max.   :5529                Max.   :17380   Max.   :18781
##
##        C               N          total_phenolics
##  Min.   :43.07   Min.   :1.721   Min.   :28.70
##  1st Qu.:43.41   1st Qu.:1.846   1st Qu.:33.73
##  Median :43.64   Median :2.004   Median :38.15
##  Mean   :43.62   Mean   :2.071   Mean   :37.61
##  3rd Qu.:43.79   3rd Qu.:2.262   3rd Qu.:41.15
##  Max.   :44.04   Max.   :2.697   Max.   :54.50
##
```

Check for biological replicates

```
# replicates per LPI
dim(resna[which(resna$LPI == 6),])[1]

## [1] 21

dim(resna[which(resna$LPI == 8),])[1]

## [1] 15

dim(resna[which(resna$LPI == 10),])[1]

## [1] 20

dim(resna[which(resna$LPI == 12),])[1]

## [1] 19

dim(resna[which(resna$LPI == 14),])[1]

## [1] 19

dim(resna[which(resna$LPI == 16),])[1]

## [1] 17

dim(resna[which(resna$LPI == 18),])[1]

## [1] 24

dim(resna[which(resna$LPI == 20),])[1]

## [1] 23

# replicates per LPI in plant1
plant1 <- resna[which(resna$plant == 'pl1'),]
dim(plant1[which(plant1$LPI == 6),])[1]

## [1] 4
```

```r
dim(plant1[which(plant1$LPI == 8),])[1]

## [1] 7

dim(plant1[which(plant1$LPI == 10),])[1]

## [1] 9

dim(plant1[which(plant1$LPI == 12),])[1]

## [1] 6

dim(plant1[which(plant1$LPI == 14),])[1]

## [1] 8

dim(plant1[which(plant1$LPI == 16),])[1]

## [1] 5

dim(plant1[which(plant1$LPI == 18),])[1]

## [1] 8

dim(plant1[which(plant1$LPI == 20),])[1]

## [1] 9

# replicates per LPI in plant2
plant2 <- resna[which(resna$plant == 'pl2'),]
dim(plant2[which(plant2$LPI == 6),])[1]

## [1] 5

dim(plant2[which(plant2$LPI == 8),])[1]

## [1] 4

dim(plant2[which(plant2$LPI == 10),])[1]

## [1] 4

dim(plant2[which(plant2$LPI == 12),])[1]

## [1] 6

dim(plant2[which(plant2$LPI == 14),])[1]

## [1] 4

dim(plant2[which(plant2$LPI == 16),])[1]

## [1] 7

dim(plant2[which(plant2$LPI == 18),])[1]
```

```
## [1] 10
dim(plant2[which(plant2$LPI == 20),])[1]
## [1] 7
# replicates per LPI in plant3
plant3 <- resna[which(resna$plant == 'pl3'),]
dim(plant3[which(plant3$LPI == 6),])[1]
## [1] 12
dim(plant3[which(plant3$LPI == 8),])[1]
## [1] 4
dim(plant3[which(plant3$LPI == 10),])[1]
## [1] 7
dim(plant3[which(plant3$LPI == 12),])[1]
## [1] 7
dim(plant3[which(plant3$LPI == 14),])[1]
## [1] 7
dim(plant3[which(plant3$LPI == 16),])[1]
## [1] 5
dim(plant3[which(plant3$LPI == 18),])[1]
## [1] 6
dim(plant3[which(plant3$LPI == 20),])[1]
## [1] 7
rm(plant1, plant2, plant3)
```

Phenotypic data exploration: PCA

Describe the raw fungal data:

```
# PCA on fungal data
pca <- dudi.pca(resna[,c('latent_period', 'uredinia_size', 'spore_volume',
'sporulation_rate', 'sporulation_capacity', 'mycelium_quantity')], center=T
, scale=T, nf=3,scannf=FALSE)
resna$plantN <- as.numeric(resna$plant)
sup <- supcol(pca,scalewt(resna[c('LPI','plantN','day')]))$cosup
p <- fviz_pca_var(pca,
```

```
             col.var = "contrib", # Color by contributions to the PC
             gradient.cols = c("#00AFBB","#FC4E07"),
             repel = TRUE)      # Avoid text overlapping
# Add experimental (LPI, plant and day) data as supplementary variables
fviz_add(p, sup, geom="arrow", linetype='solid', color ="black")
```

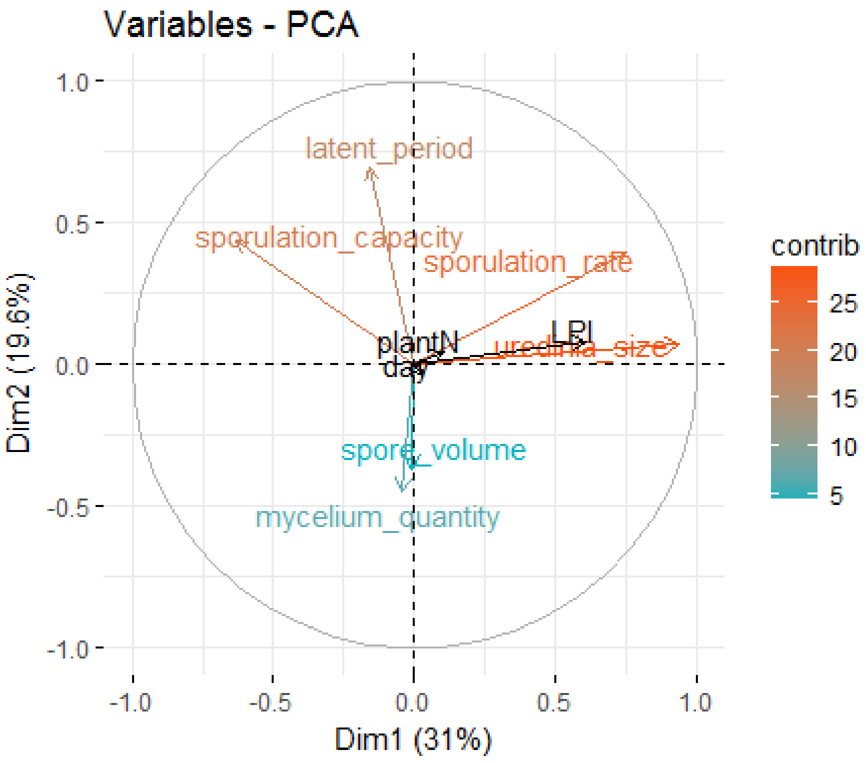


```
# color scale for LPI representation on 1st and 2nd axis
cc <- scales::seq_gradient_pal("blue", "red", "Lab")(seq(0,1,length.out=8))
s.class(dfxy = pca$li, fac = as.factor(resna$LPI),col = cc, clabel = 0.9, g
rid= 0, xax = 1, yax = 2, add.plot= F, cellipse = F)
```

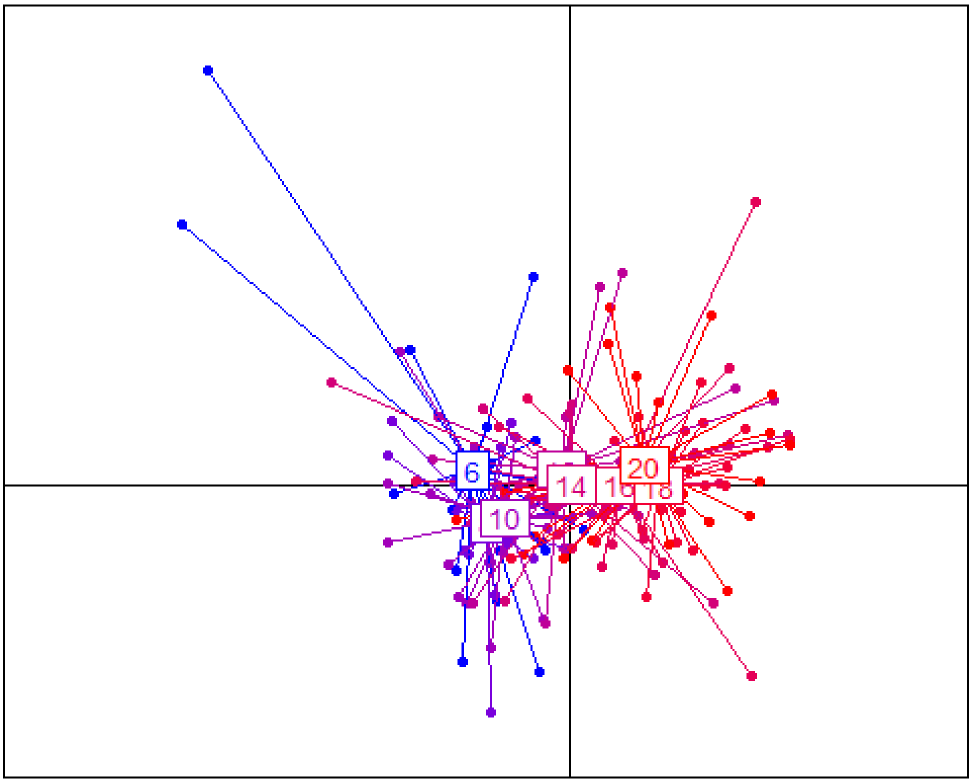

Uredinia size and sporulation rate shape the 1st axis. The experimental variable LPI well explained this 1st axis even if some residual variance is not explained. Noteworthy the two other experimental variables (plant and day) do not contributed to the 1st or 2nd axis. This is consistent with GLM results: LPI has a significant effect on uredinia size and sporulation rate.

```
# PCA representation of 1st and 3rd axis
fviz_pca_var(pca, axes = c(2, 3),
             col.var = "contrib", # Color by contributions to the PC
             gradient.cols = c("#00AFBB","#FC4E07"),
             repel = TRUE)     # Avoid text overlapping
```

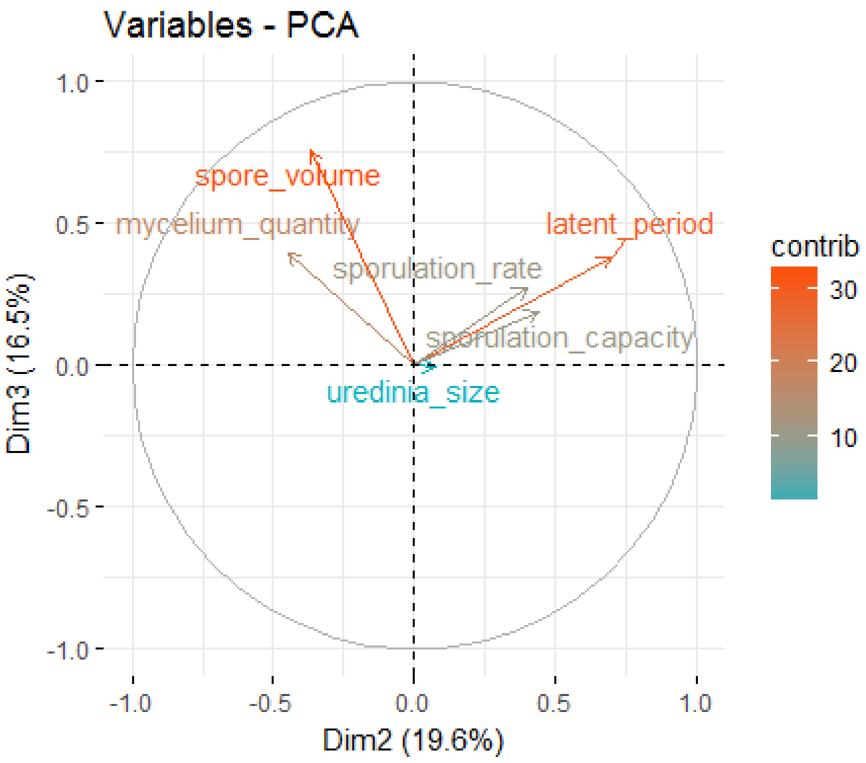


```r
# PCA variances explained on the 3 first axis
pca.var<-data.frame(pca$co)
kable(pca.var)
```

	Comp1	Comp2	Comp3
latent_period	-0.1580970	0.6958418	0.3825418
uredinia_size	0.9344581	0.0729967	-0.0021118
spore_volume	-0.0110515	-0.3672860	0.7577132
sporulation_rate	0.7473166	0.3964454	0.2771676
sporulation_capacity	-0.6344263	0.4396288	0.1862767
mycelium_quantity	-0.0453659	-0.4492560	0.3995747

Spore volume and latent period shape the 2nd axis. Mycelium quantity shapes the 3rd axis. These axes are not explained by experimental variables (LPI, plant and day) but by something else not taken into account in our experimental design.

Test for experimental data effect on fungus traits: Barplots and GLM

Color gradient

```r
cc <- scales::seq_gradient_pal("blue", "red", "Lab")(seq(0,0.9,length.out=8
))
```

Data transformation to better fit normality
Error family:
infection efficiency: binomial family
latent period: Gamma family, link= identity

```
shapiro.test(results$uredinia_size) # not normal

##
##  Shapiro-Wilk normality test
##
## data:  results$uredinia_size
## W = 0.93982, p-value = 1.792e-06

shapiro.test(sqrt(results$uredinia_size)) # much better

##
##  Shapiro-Wilk normality test
##
## data:  sqrt(results$uredinia_size)
## W = 0.98341, p-value = 0.04482

shapiro.test(results$spore_volume) # normal

##
##  Shapiro-Wilk normality test
##
## data:  results$spore_volume
## W = 0.98802, p-value = 0.1927

shapiro.test(results$sporulation_rate) # not normal

##
##  Shapiro-Wilk normality test
##
## data:  results$sporulation_rate
## W = 0.93841, p-value = 2.195e-06

shapiro.test(sqrt(results$sporulation_rate)) # normal

##
##  Shapiro-Wilk normality test
##
## data:  sqrt(results$sporulation_rate)
## W = 0.98702, p-value = 0.1466

shapiro.test(results$sporulation_capacity) # not normal

##
##  Shapiro-Wilk normality test
##
```

```
## data:  results$sporulation_capacity
## W = 0.54072, p-value < 2.2e-16
```

```r
shapiro.test(results$mycelium_quantity) # not normal
```

```
##
##  Shapiro-Wilk normality test
##
## data:  results$mycelium_quantity
## W = 0.9178, p-value = 4.876e-08
```

```r
shapiro.test(sqrt(results$mycelium_quantity)) # normal
```

```
##
##  Shapiro-Wilk normality test
##
## data:  sqrt(results$mycelium_quantity)
## W = 0.98487, p-value = 0.07016
```

uredinia size: square root transformation, Gaussian family
spore volume: Gaussian family
sporulation rate: square root transformation, Gaussian family
sporulation capacity: Gamma family, link= inverse
mycelium quantity: square root transformation, Gaussian family

Experimental data effect on infection efficiency

```r
# Distribution
hist(results$infection_efficiency, col='blue')
```

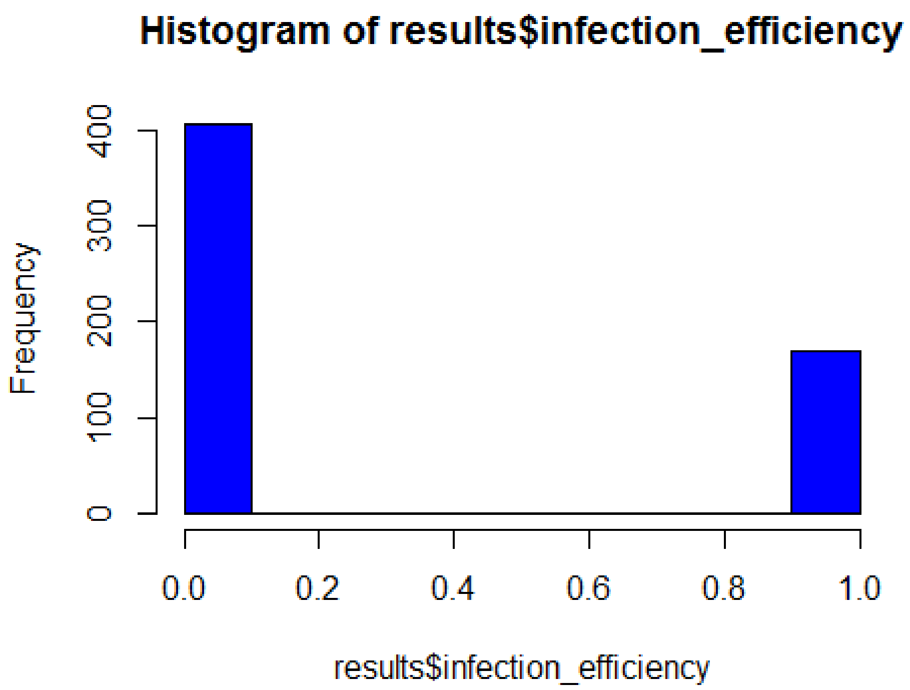


```r
# Infection efficiency per LPI
graphe=with(results,bargraph.CI(x.factor=LPI,response=infection_efficiency,
fun=mean,xlab="LPI",ylab="Infection efficiency (%)",main="Infection efficie
ncy for each leaf maturity",col=cc, ylim=c(0,0.5), xpd=T,legend = T, lc=T,u
c=T,ci.fun= function(x) c(mean(x)-se(x), mean(x) + se(x))))
```

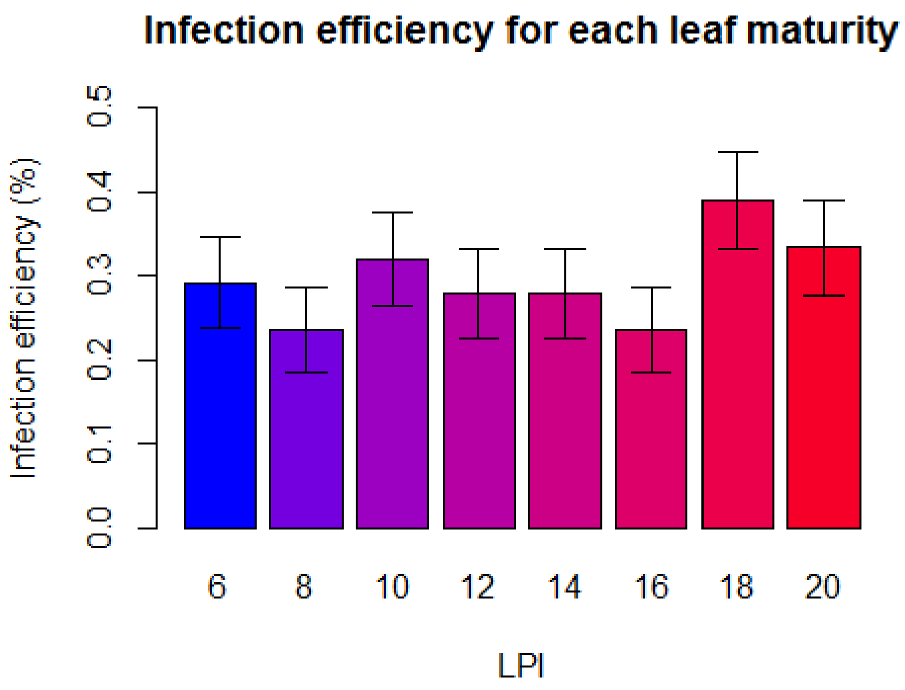

```
# Infection efficiency per plant
graphe=with(results,bargraph.CI(x.factor=plant,response=infection_efficienc
y,fun=mean,xlab="Plant",ylab="Infection efficiency (%)",main="Infection eff
iciency for each plant",col='grey',ylim=c(0,0.5),xpd=T,legend = T, lc=T,uc=
T,ci.fun= function(x) c(mean(x)-se(x), mean(x) + se(x))))
```

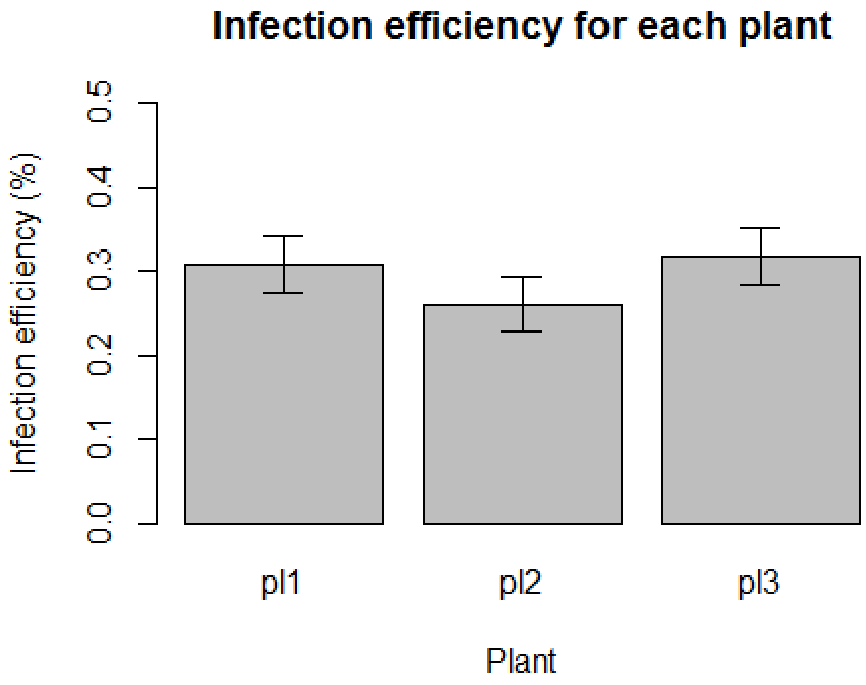


```
# Infection efficiency per inoculation day
graphe=with(results,bargraph.CI(x.factor=day,response=infection_efficiency,
fun=mean,xlab="Day",ylab="Infection efficiency (%)",main="Infection efficie
ncy for each inoculation day",col='grey',ylim=c(0,0.5),xpd=T,legend = T, lc
=T,uc=T,ci.fun= function(x) c(mean(x)-se(x), mean(x) + se(x))))
```

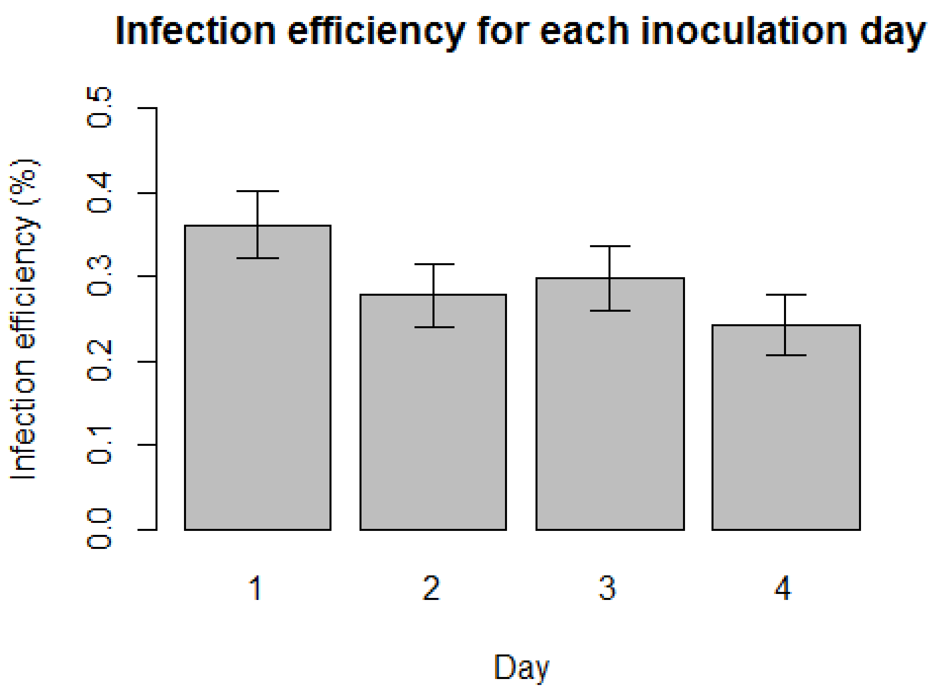

GLM

```
glmeff = glm(infection_efficiency ~ day + LPI*plant, results, family = bino
mial)
plot(glmeff, ask=F)
```

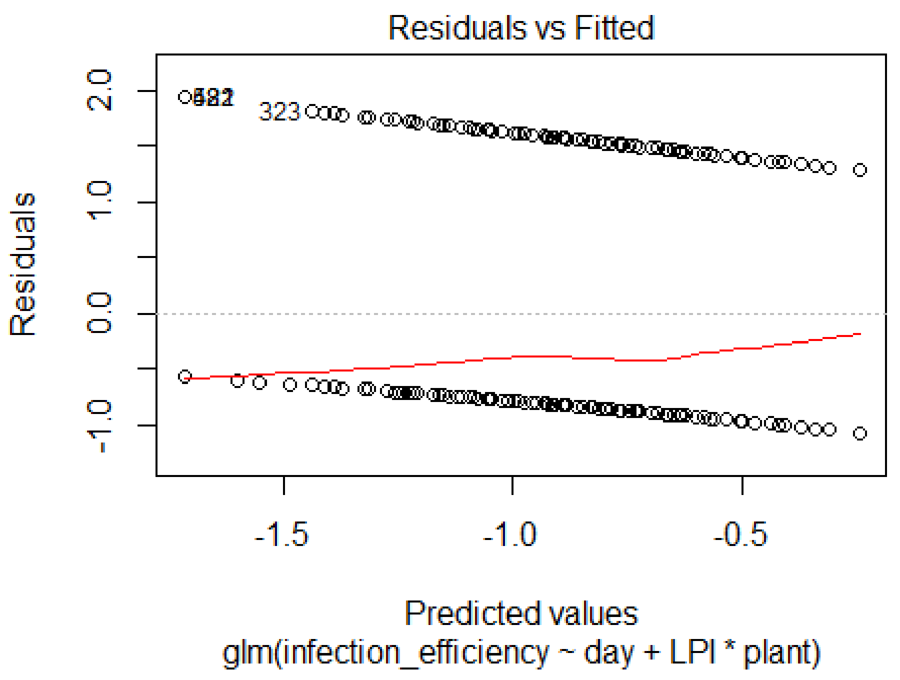
Residuals vs Fitted
Residuals
2.0
1.0
0.0
-1.0
323
-1.5
-1.0
-0.5
Predicted values
glm(infection_efficiency ~ day + LPI * plant)

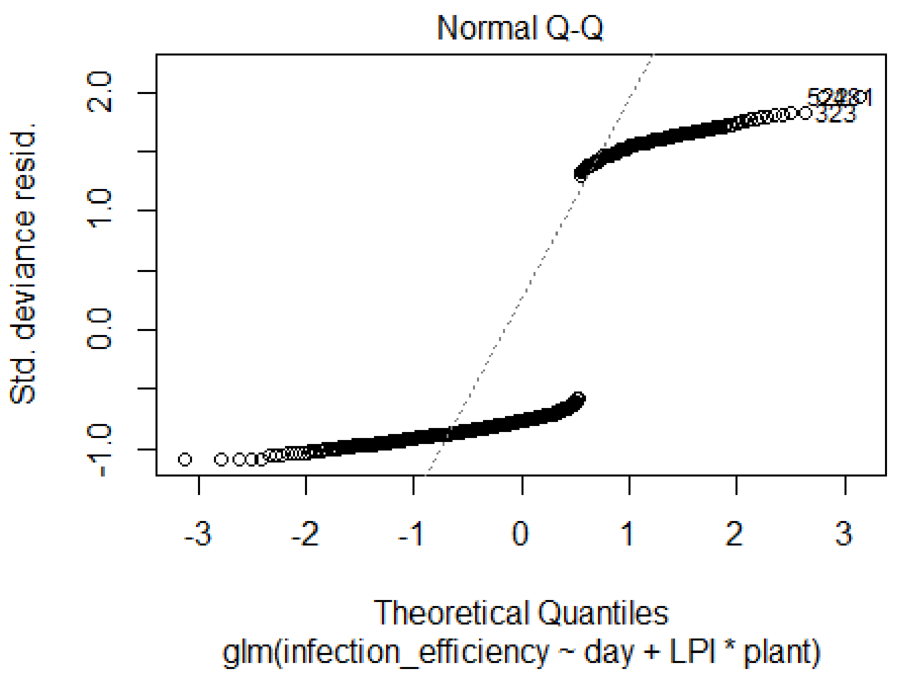
Normal Q-Q
Std. deviance resid.
2.0
1.0
0.0
-1.0
323
-3
-2
-1
0
1
2
3
Theoretical Quantiles
glm(infection_efficiency ~ day + LPI * plant)

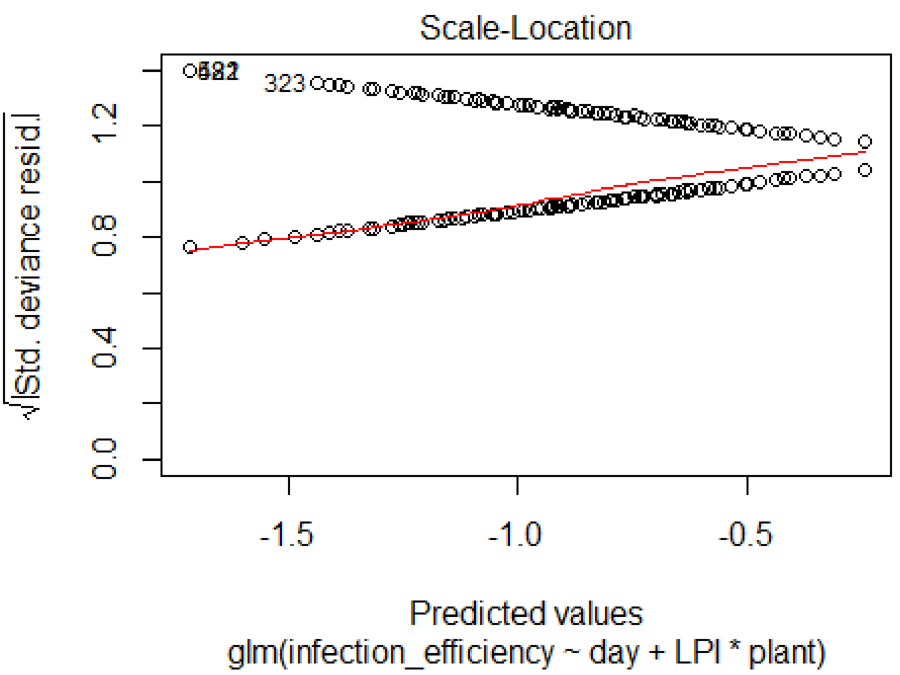

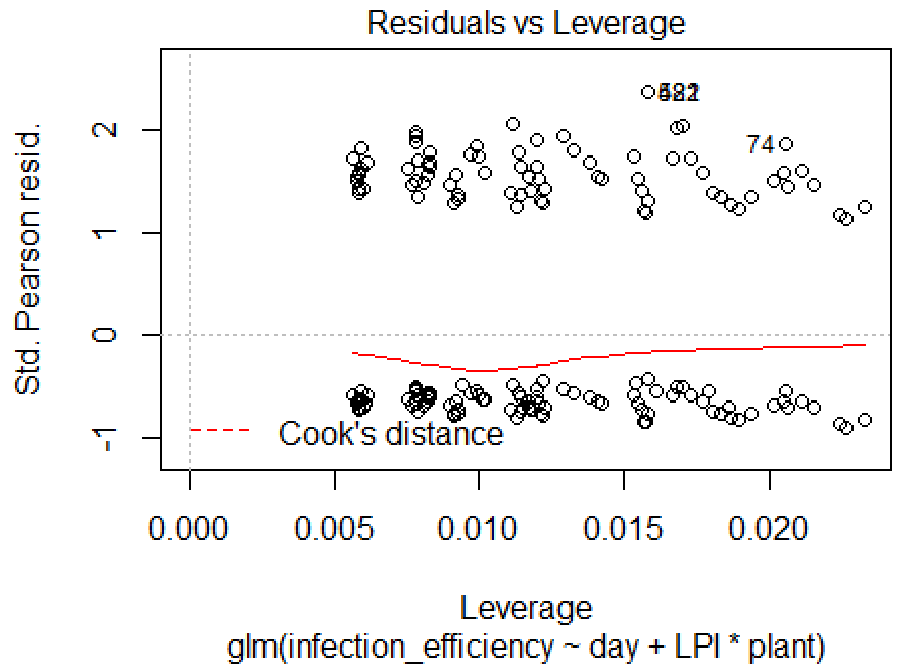


```
summary(glmeff) # Sign : Intercept, day, plant

##
## Call:
## glm(formula = infection_efficiency ~ day + LPI * plant, family = binomia
l,
##     data = results)
```

```
##
## Deviance Residuals:
##     Min       1Q   Median       3Q      Max
## -1.0754  -0.8738  -0.7607   1.3957   1.9399
##
## Coefficients:
##                 Estimate Std. Error z value Pr(>|z|)
## (Intercept)   -1.051171   0.527589  -1.992   0.0463 *
## day           -0.162994   0.082825  -1.968   0.0491 *
## LPI            0.048469   0.034643   1.399   0.1618
## plantpl2      -0.358953   0.716938  -0.501   0.6166
## plantpl3       1.090348   0.673628   1.619   0.1055
## LPI:plantpl2   0.009178   0.050390   0.182   0.8555
## LPI:plantpl3  -0.079680   0.048616  -1.639   0.1012
## ---
## Signif. codes:  0 '***' 0.001 '**' 0.01 '*' 0.05 '.' 0.1 ' ' 1
##
## (Dispersion parameter for binomial family taken to be 1)
##
##     Null deviance: 698.91  on 575  degrees of freedom
## Residual deviance: 687.96  on 569  degrees of freedom
## AIC: 701.96
##
## Number of Fisher Scoring iterations: 4
```

```
aov.eff <- anova(glmeff,test="Chisq")
kable(aov.eff) # Sign: None
```

	Df	Deviance	Resid. Df	Resid. Dev	Pr(>Chi)
NULL	NA	NA	575	698.9058	NA
day	1	3.854980	574	695.0508	0.0495985
LPI	1	1.347044	573	693.7038	0.2457956
plant	2	1.754594	571	691.9492	0.4159056
LPI:plant	2	3.992344	569	687.9568	0.1358544

No experimental effect on infection efficiency.

Experimental data effect on latent period

```
# Distribution
hist(results$latent_period, col='blue')
```

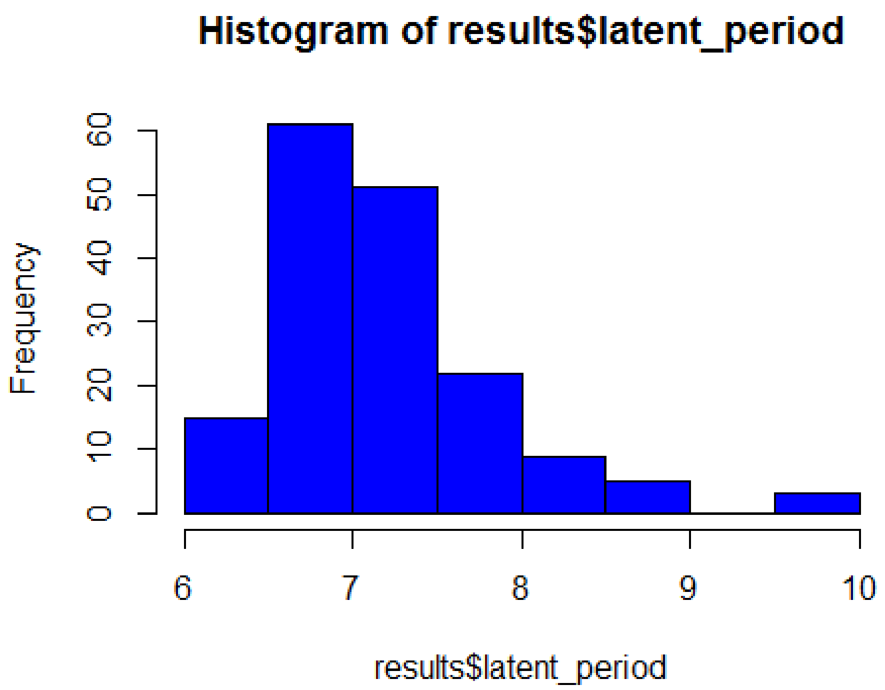


```
# Latent period per LPI
graphe=with(resna,bargraph.CI(x.factor=LPI,response=latent_period,fun=mean,
xlab="LPI",ylab="Latent period (days)",main="Latent period for each leaf ma
turity",col=cc,xpd=F,legend = T, lc=T,uc=T, ylim=c(5,9),ci.fun= function(x)
c(mean(x)-se(x), mean(x) + se(x))))
```

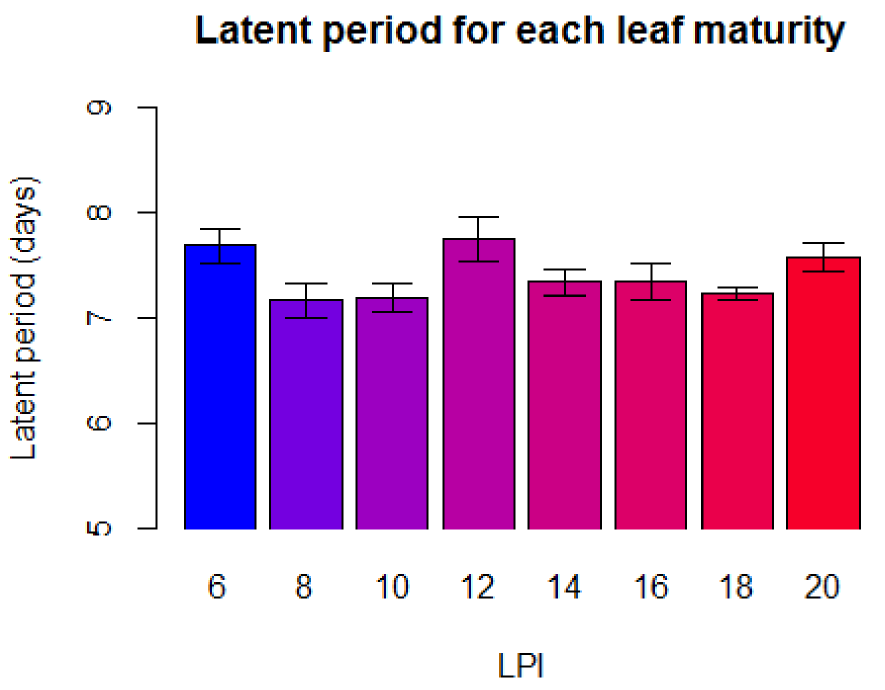

```r
# Latent period per plant
graphe=with(resna,bargraph.CI(x.factor=plant,response=latent_period,fun=mea
n,xlab="Plant",ylab="Latent period (days)",main="Latent period for each pla
nt",col='grey',xpd=F,legend = T, lc=T,uc=T, ylim=c(5,9),ci.fun= function(x)
c(mean(x)-se(x), mean(x) + se(x))))
```

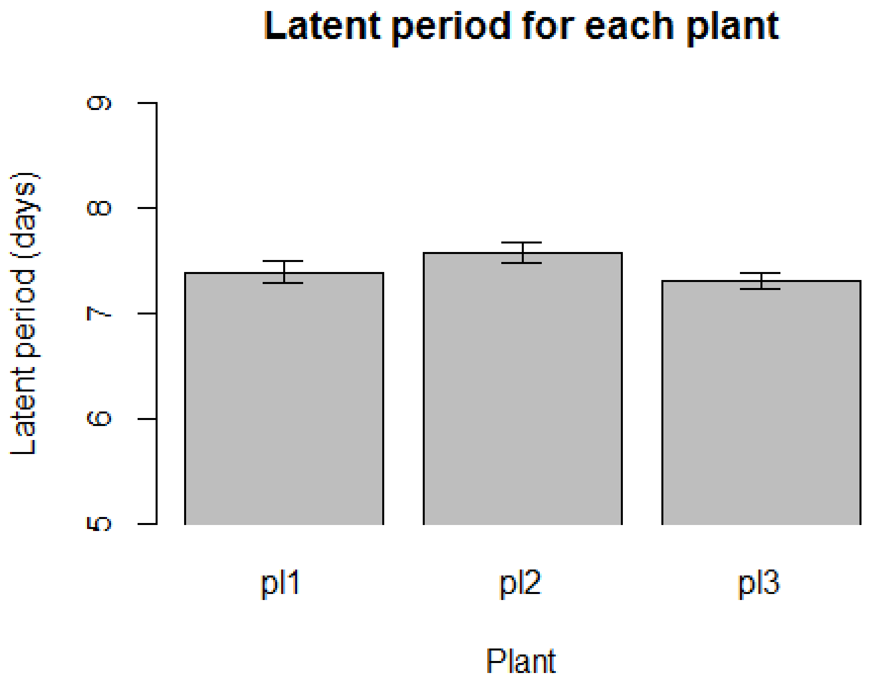


```r
# Latent period per inoculation day
graphe=with(resna,bargraph.CI(x.factor=day,response=latent_period,fun=mean,
xlab="Day",ylab="Latent period (days)",main="Latent period for each inocula
tion day",col='grey',xpd=F,legend = T, lc=T,uc=T, ylim=c(5,9),ci.fun= funct
ion(x) c(mean(x)-se(x), mean(x) + se(x))))
```

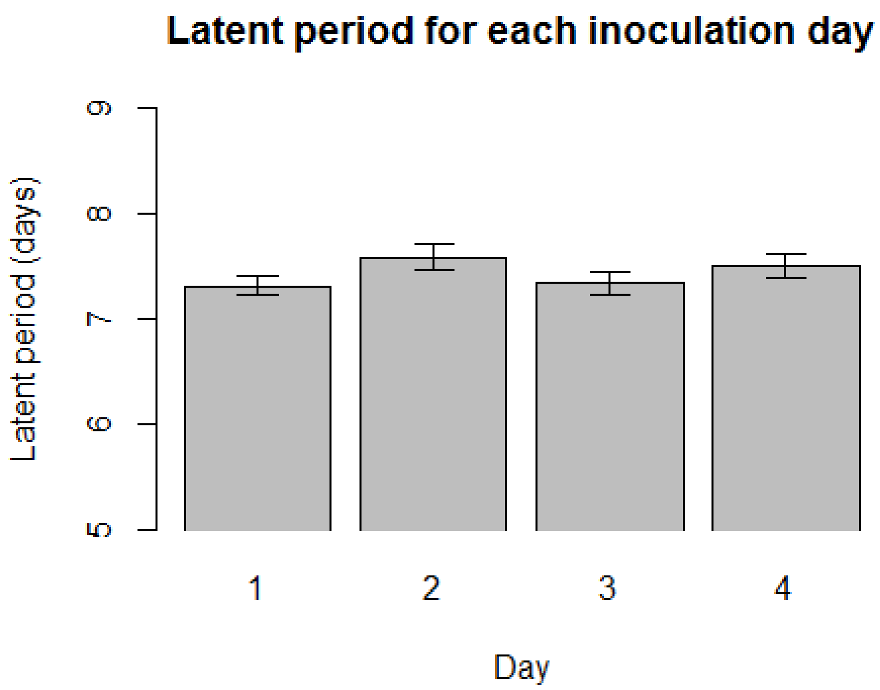

GLM

```
glmlat = glm(latent_period ~ day + LPI*plant, results, family = Gamma(link=
identity))
plot(glmlat, ask=F)
```

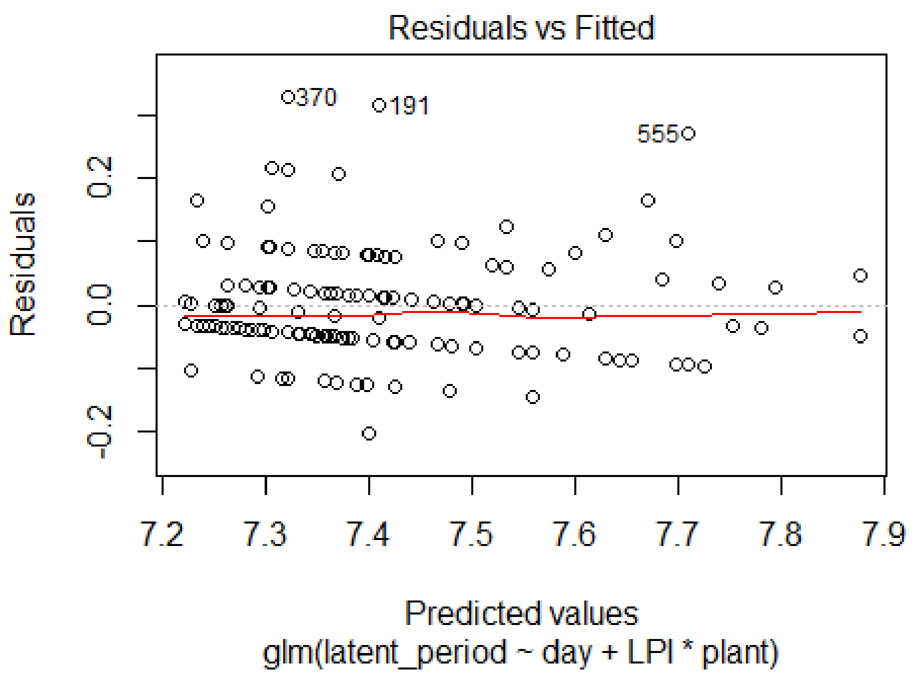
Residuals vs Fitted
Residuals
-0.2
0.0
0.2
370
191
555
7.2
7.3
7.4
7.5
7.6
7.7
7.8
7.9
Predicted values
glm(latent_period ~ day + LPI * plant)

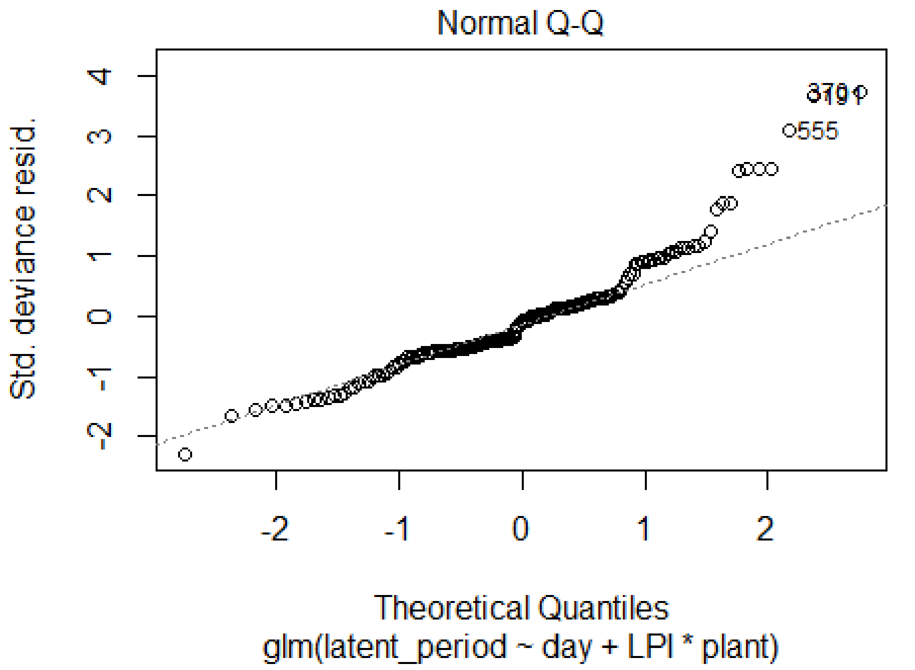
Normal Q-Q
Std. deviance resid.
-2
-1
0
1
2
3
4
555
Theoretical Quantiles
glm(latent_period ~ day + LPI * plant)

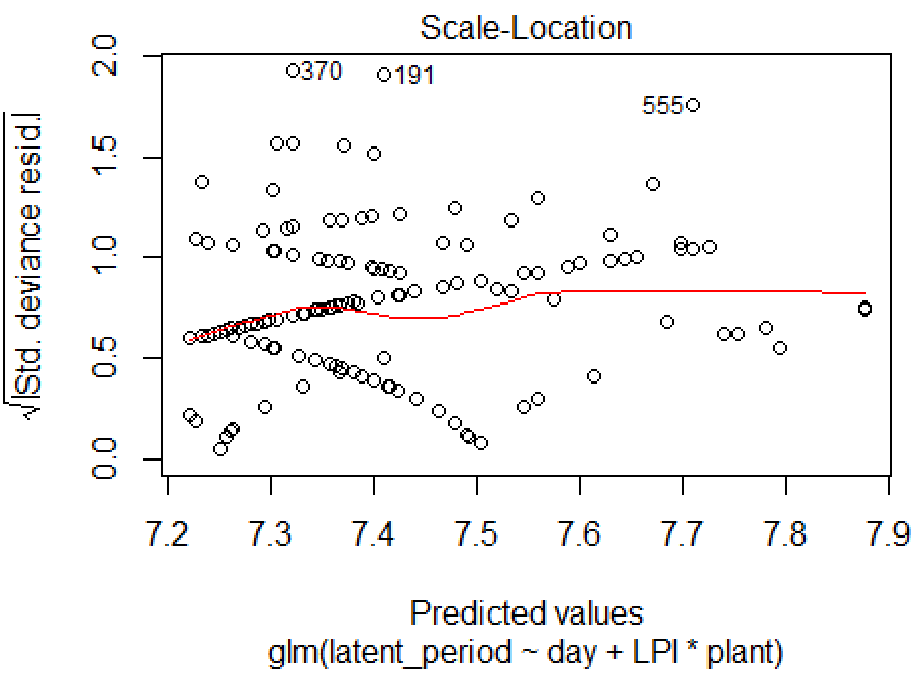

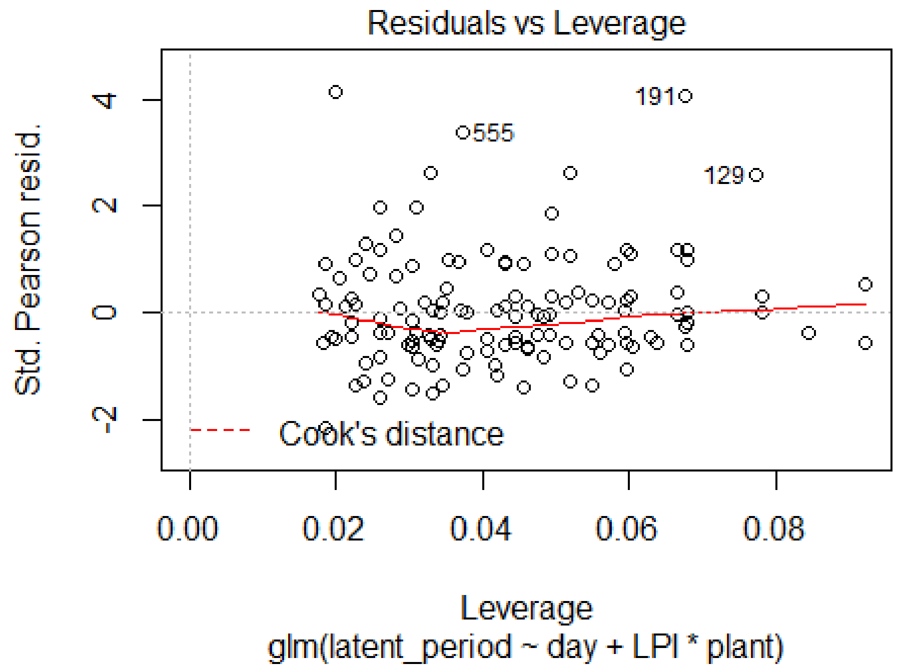


```
summary(glmlat) # Sign : Intercept

##
## Call:
## glm(formula = latent_period ~ day + LPI * plant, family = Gamma(link = i
dentity),
##     data = results)
```

```
##
## Deviance Residuals:
##      Min        1Q    Median        3Q       Max
## -0.20271  -0.05140  -0.00952   0.02678   0.32892
##
## Coefficients:
##                 Estimate Std. Error t value Pr(>|t|)
## (Intercept)   7.367096   0.296804  24.821   <2e-16 ***
## day           0.040862   0.046537   0.878    0.381
## LPI          -0.006368   0.019083  -0.334    0.739
## plantpl2      0.510198   0.416502   1.225    0.222
## plantpl3     -0.205383   0.364056  -0.564    0.573
## LPI:plantpl2 -0.021166   0.028456  -0.744    0.458
## LPI:plantpl3  0.009429   0.026138   0.361    0.719
## ---
## Signif. codes:  0 '***' 0.001 '**' 0.01 '*' 0.05 '.' 0.1 ' ' 1
##
## (Dispersion parameter for Gamma family taken to be 0.007991393)
##
##     Null deviance: 1.2558  on 165  degrees of freedom
## Residual deviance: 1.1912  on 159  degrees of freedom
##   (410 observations deleted due to missingness)
## AIC: 331.55
##
## Number of Fisher Scoring iterations: 4
```

```
aov.lat <- anova(glmlat,test="Chisq")
kable(aov.lat) # Sign: none
```

	Df	Deviance	Resid. Df	Resid. Dev	Pr(>Chi)
NULL	NA	NA	165	1.255796	NA
day	1	0.0050837	164	1.250713	0.4251101
LPI	1	0.0024763	163	1.248236	0.5777575
plant	2	0.0470148	161	1.201222	0.0527818
LPI:plant	2	0.0099845	159	1.191237	0.5354210

No experimental effect of latent period.

Experimental data effect on uredinia size

```
# Distribution
hist(results$uredinia_size, col='blue')
```

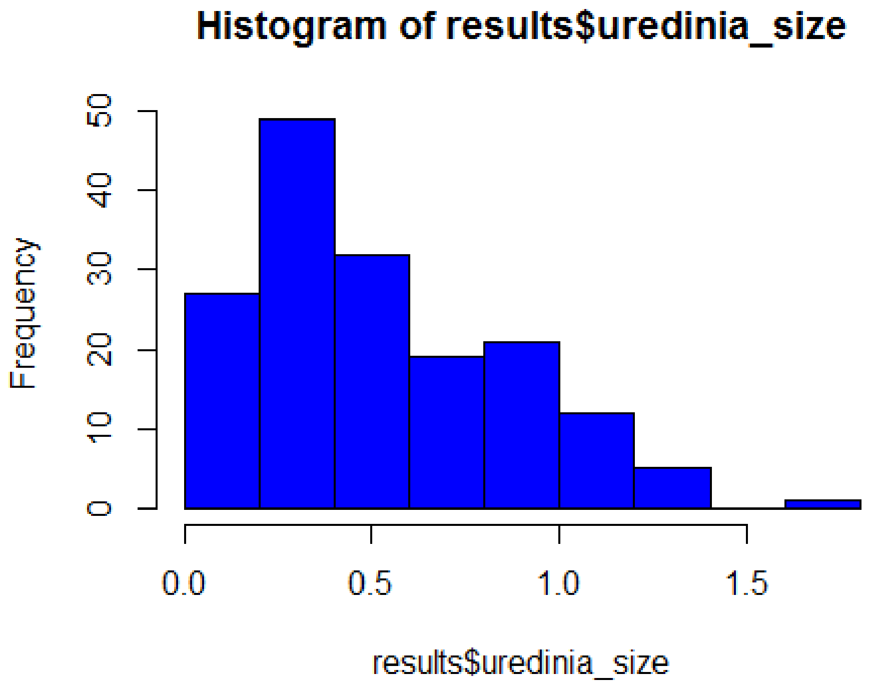


```r
# Uredinia size per LPI
graphe=with(resna,bargraph.CI(x.factor=LPI,response=uredinia_size,fun=mean,
xlab="LPI",ylab="Uredinia size (mm2)", main="Uredinia size for each leaf ma
turity", col=cc, ylim=c(0,1), xpd=T,legend = T, lc=T,uc=T, ci.fun= function
(x) c(mean(x)-se(x), mean(x) + se(x))))
text(graphe$xvals,graphe$vals+0.15,c('c', 'c', 'c', 'bc', 'bc', 'ab', 'a',
'ab')) # add Tukey coefficients
```

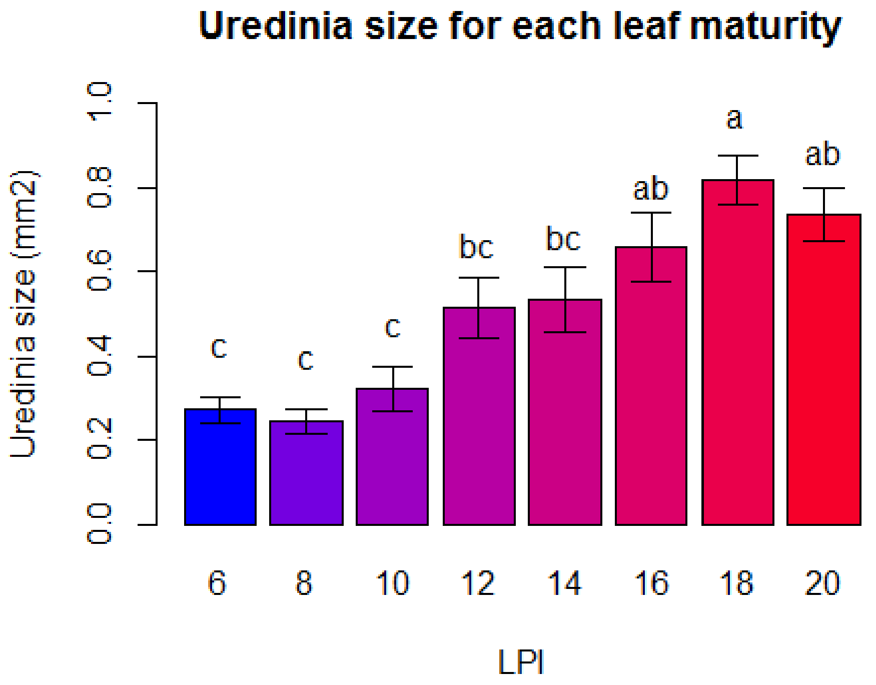


```
# Uredinia size per plant
graphe=with(resna,bargraph.CI(x.factor=plant,response=uredinia_size,fun=mea
n,xlab="Plant",ylab="Uredinia size mm2)",main="Uredinia size for each plant
", col='grey', ylim=c(0,1), xpd=T,legend = T, lc=T,uc=T, ci.fun= function(x
) c(mean(x)-se(x), mean(x) + se(x))))
```

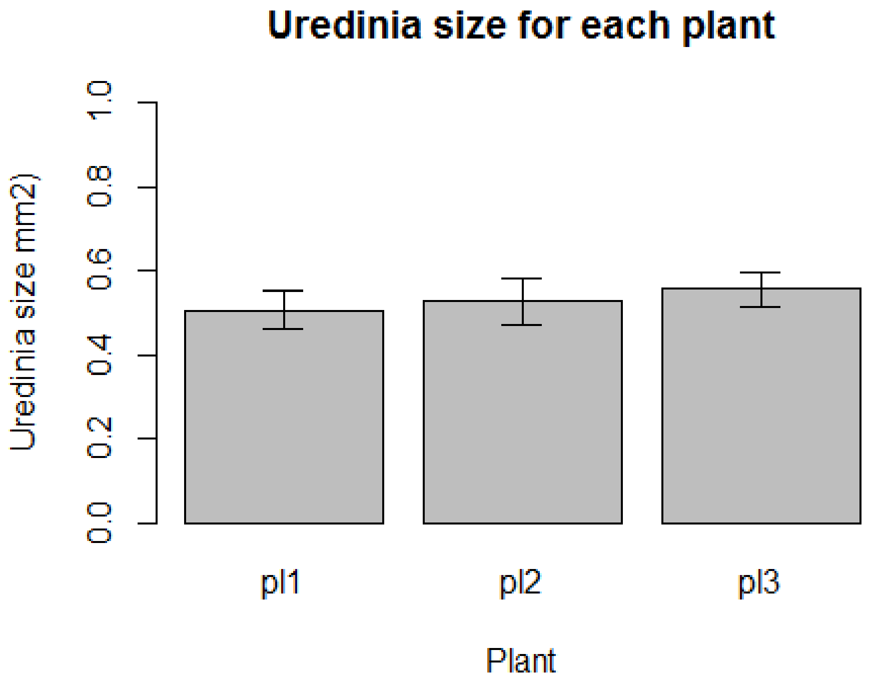

```r
# Uredinia size per inoculation day
graphe=with(resna,bargraph.CI(x.factor=day,response=uredinia_size,fun=mean,
xlab="Day",ylab="Uredinia size (mm2)",main="Uredinia size for each inoculat
ion day", col='grey', ylim=c(0,1), xpd=T,legend = T, lc=T,uc=T, ci.fun= fun
ction(x) c(mean(x)-se(x), mean(x) + se(x))))
```

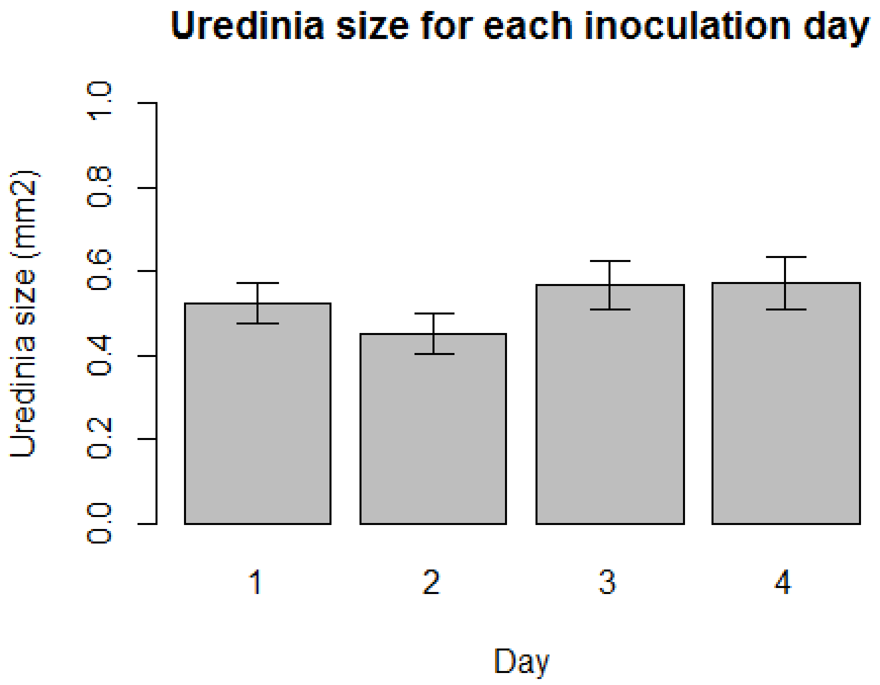

GLM

```r
glmus = glm(sqrt(uredinia_size) ~ day + LPI*plant, results, family = gaussi
an)
plot(glmus, ask=F)
```

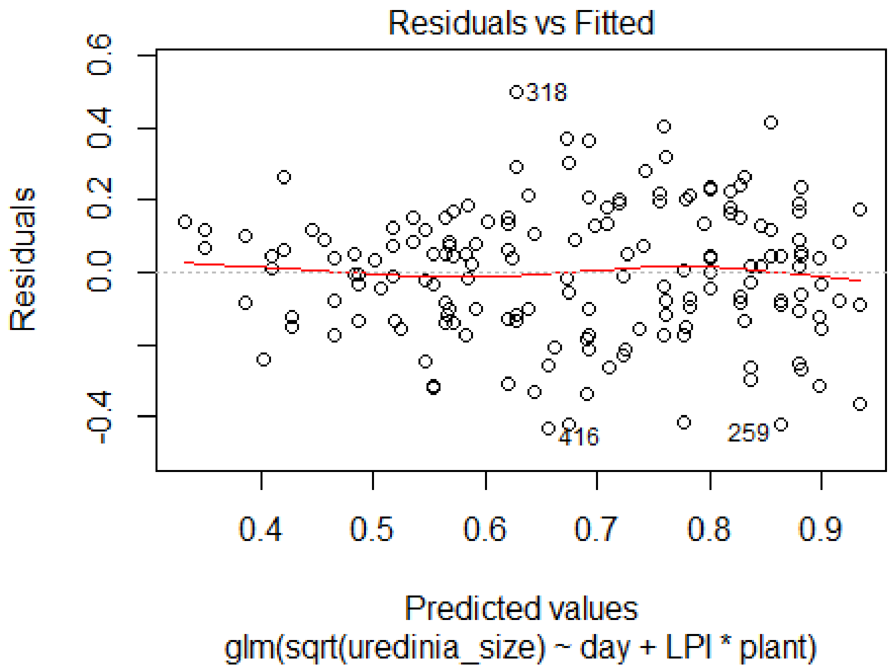
Residuals vs Fitted
318
416
259
Residuals
-0.4
0.0
0.2
0.4
0.6
0.4
0.5
0.6
0.7
0.8
0.9
Predicted values
glm(sqrt(uredinia_size) ~ day + LPI * plant)

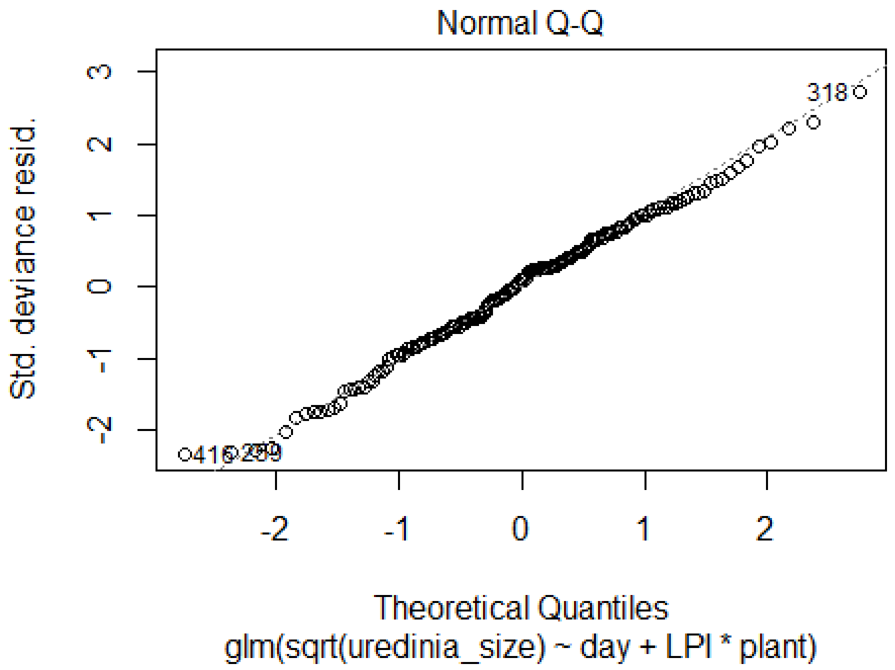
Normal Q-Q
318
416
259
Std. deviance resid.
-2
-1
0
1
2
3
-2
-1
0
1
2
Theoretical Quantiles
glm(sqrt(uredinia_size) ~ day + LPI * plant)

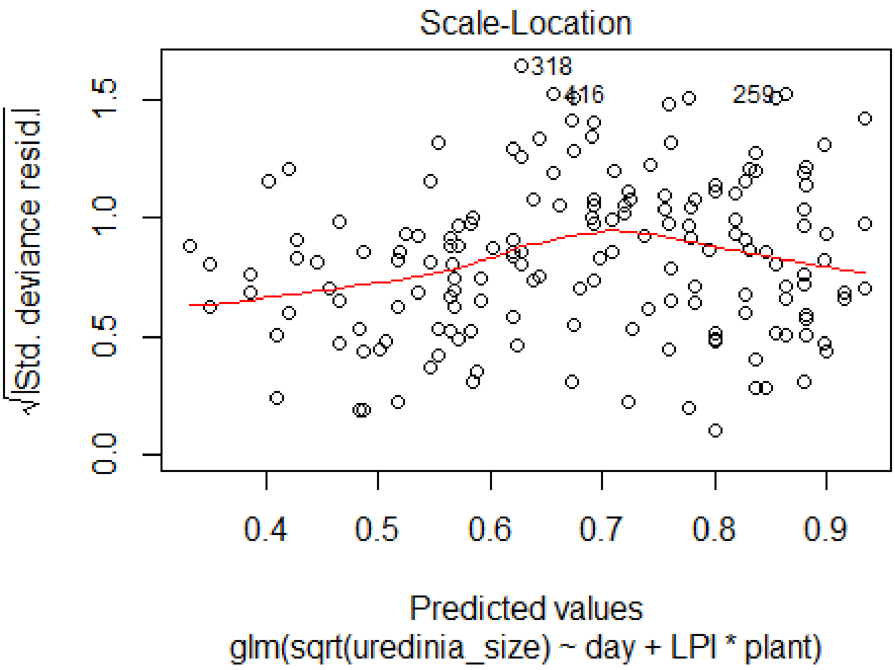

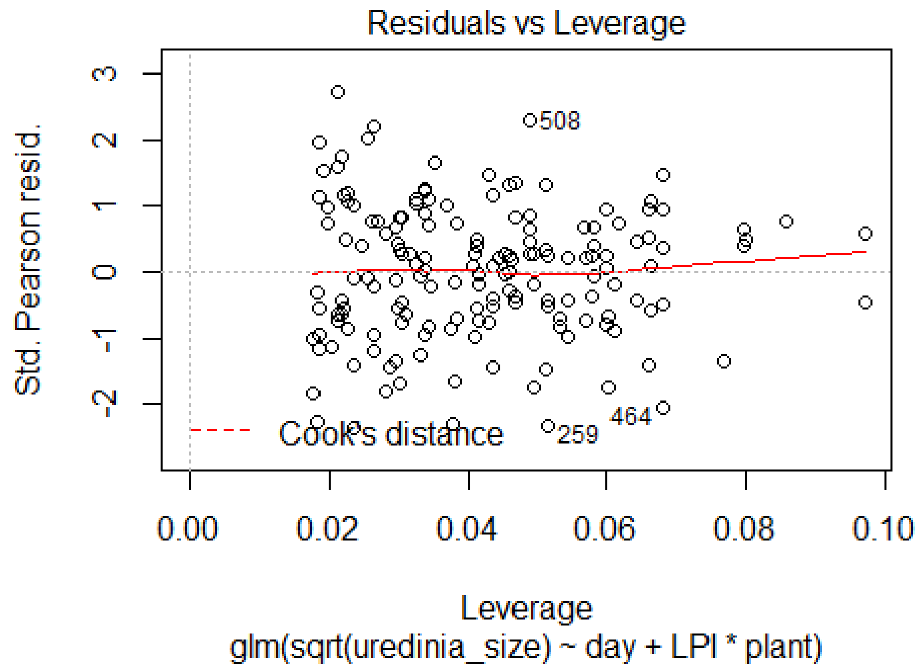


```
summary(glmus) # Sign : LPI

##
## Call:
## glm(formula = sqrt(uredinia_size) ~ day + LPI * plant, family = gaussian
,
##     data = results)
```

```
##
## Deviance Residuals:
##      Min        1Q    Median        3Q       Max
## -0.43038  -0.12384   0.01469   0.13009   0.49962
##
## Coefficients:
##               Estimate Std. Error t value Pr(>|t|)
## (Intercept)   0.194478   0.083547   2.328   0.0212 *
## day           0.018056   0.013060   1.383   0.1687
## LPI           0.031655   0.005383   5.881 2.33e-08 ***
## plantpl2     -0.115543   0.114705  -1.007   0.3153
## plantpl3      0.149476   0.103124   1.449   0.1492
## LPI:plantpl2  0.007458   0.007885   0.946   0.3456
## LPI:plantpl3 -0.005764   0.007414  -0.777   0.4380
## ---
## Signif. codes:  0 '***' 0.001 '**' 0.01 '*' 0.05 '.' 0.1 ' ' 1
##
## (Dispersion parameter for gaussian family taken to be 0.0346197)
##
##     Null deviance: 9.3355  on 165  degrees of freedom
## Residual deviance: 5.5045  on 159  degrees of freedom
##   (410 observations deleted due to missingness)
## AIC: -78.377
##
## Number of Fisher Scoring iterations: 2
```

```
aov.us <- anova(glmus,test="Chisq")
kable(aov.us) # LPI***
```

	Df	Deviance	Resid. Df	Resid. Dev	Pr(>Chi)
NULL	NA	NA	165	9.335536	NA
day	1	0.0767535	164	9.258783	0.1364938
LPI	1	3.3899649	163	5.868818	0.0000000
plant	2	0.2623572	161	5.606460	0.0226152
LPI:plant	2	0.1019284	159	5.504532	0.2294396

```
Tukey_LPI <- HSD.test(glmus,"LPI",group=TRUE,main="Uredinia size for each L
PI")
kable(Tukey_LPI$groups)
```

	sqrt(uredinia_size)	groups
18	0.8772363	a
20	0.8423182	ab
16	0.7827109	ab
12	0.6872449	bc

14	0.6759903	bcd
10	0.5297664	cde
6	0.4978778	de
8	0.4813555	e

LPI effect on uredinia size.

Experimental data effect on spore volume

```
# Distribution
hist(results$spore_volume, col='blue')
```

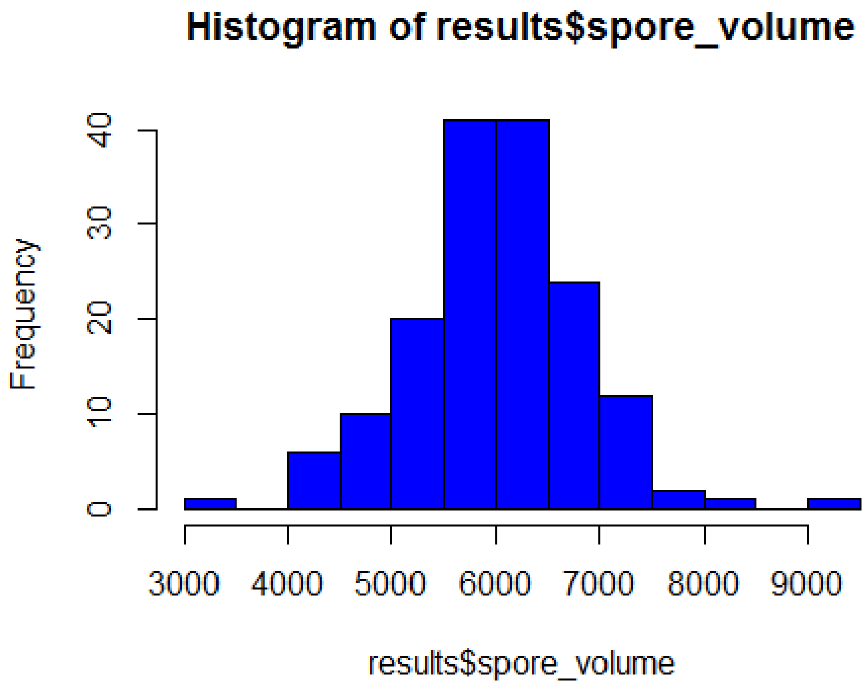


```
# Spore volume per LPI
graphe=with(resna,bargraph.CI(x.factor=LPI,response=spore_volume,fun=mean,x
lab="LPI",ylab="Spore volume ()",main="Spore volume for each leaf maturity"
, col='forestgreen',ylim=c(0,9000),xpd=T,legend = T, lc=T,uc=T, ci.fun= fun
ction(x) c(mean(x)-se(x), mean(x) + se(x))))
```

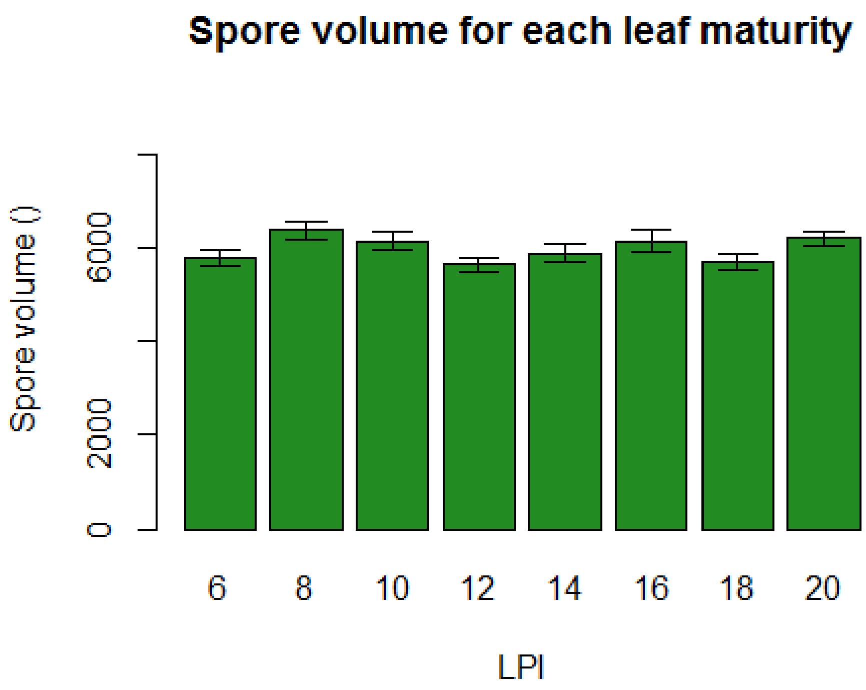


```
# Spore volume per plant
graphe=with(resna,bargraph.CI(x.factor=plant,response=spore_volume,fun=mean
,xlab="Plant",ylab="Spore volume ()",main="Spore volume for each plant",col
='grey',ylim=c(0,9000),xpd=T,legend = T, lc=T,uc=T, ci.fun= function(x) c(m
ean(x)-se(x), mean(x) + se(x))))
```

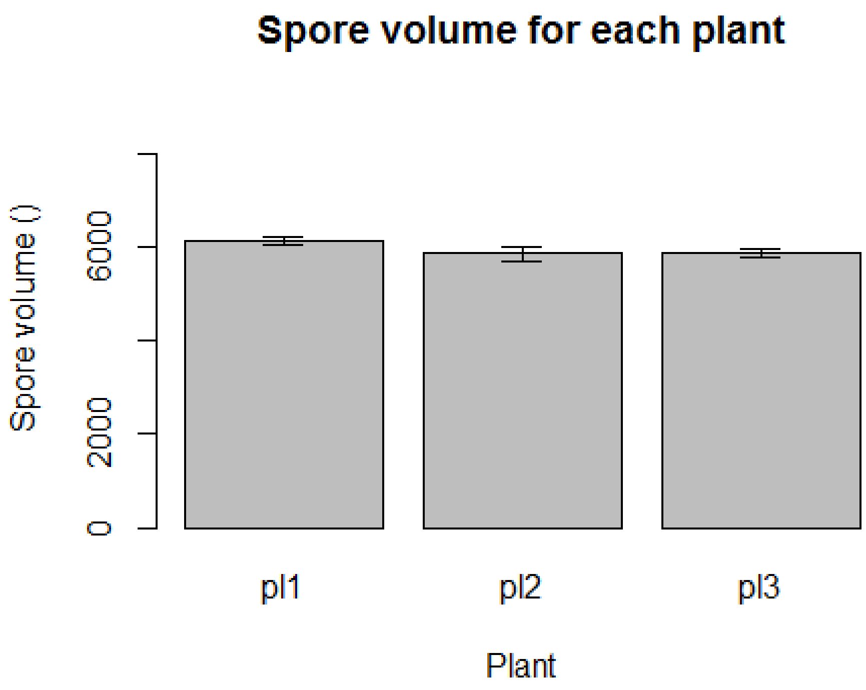

```r
# Spore volume per inoculation day
graphe=with(resna,bargraph.CI(x.factor=day,response=spore_volume,fun=mean,xlab="Day",ylab="Spore volume ()",main="Spore volume for each inoculation day", col='grey', ylim=c(0,9000), xpd=T,legend = T, lc=T,uc=T, ci.fun= function(x) c(mean(x)-se(x), mean(x) + se(x))))
```

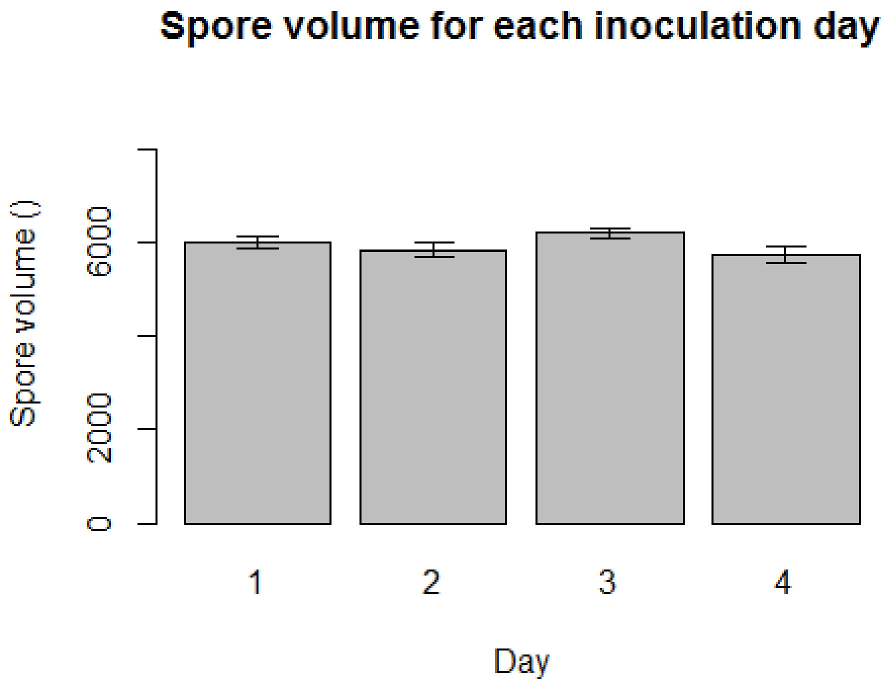

GLM

```r
glmvol = glm(spore_volume ~ day + LPI*plant, results, family = gaussian)
plot(glmvol, ask=F)
```

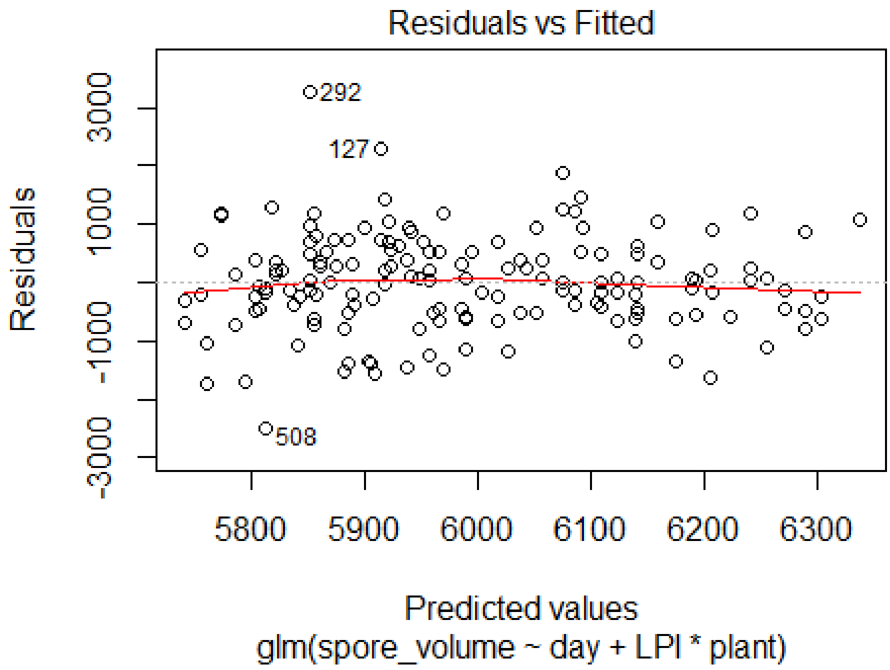
Residuals vs Fitted
Residuals
3000
1000
-1000
-3000
292
127
508
5800
5900
6000
6100
6200
6300
Predicted values
glm(spore_volume ~ day + LPI * plant)

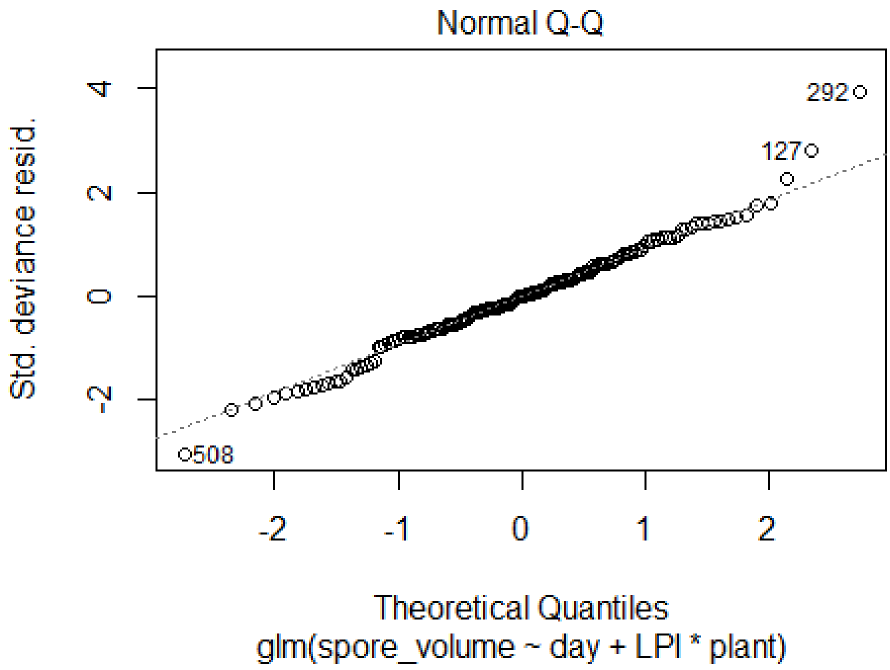
Normal Q-Q
Std. deviance resid.
4
2
0
-2
292
127
508
-2
-1
0
1
2
Theoretical Quantiles
glm(spore_volume ~ day + LPI * plant)

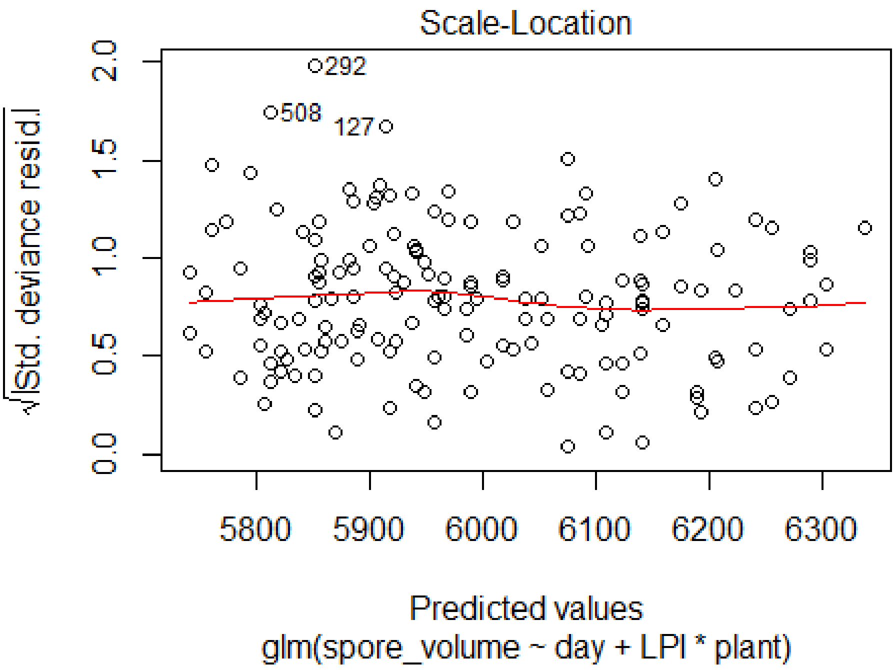

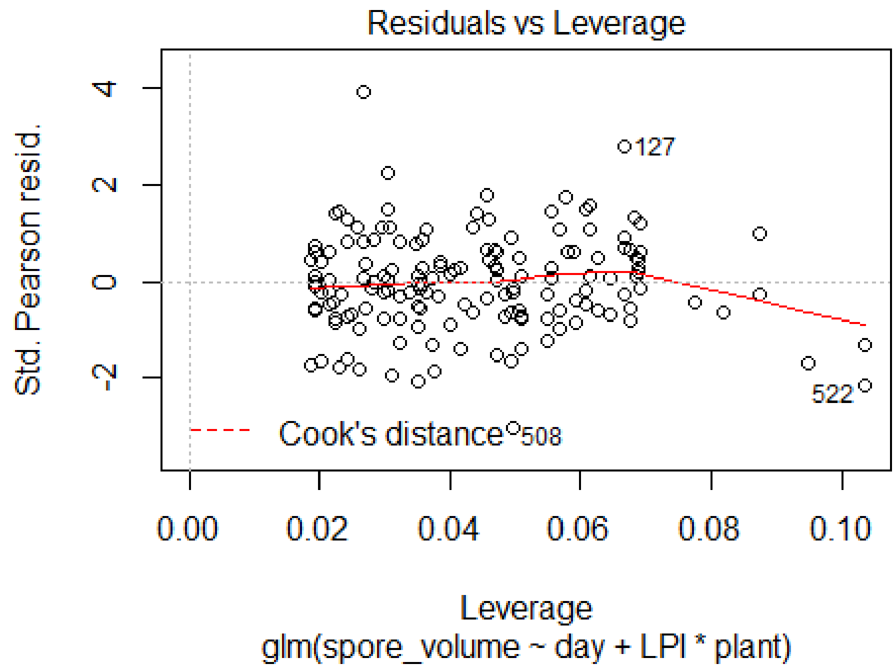


```
summary(glmvol) # Sign : Intercept

##
## Call:
## glm(formula = spore_volume ~ day + LPI * plant, family = gaussian,
##     data = results)
##
```

```
## Deviance Residuals: 
##     Min       1Q   Median       3Q      Max  
## -2486.2   -516.1      1.4    512.4   3257.5  
## 
## Coefficients:
##               Estimate Std. Error t value Pr(>|t|)    
## (Intercept)    6054.69     381.32  15.878   <2e-16 ***
## day             -48.26      60.49  -0.798    0.426    
## LPI              16.52      25.00   0.661    0.510    
## plantpl2       -128.87     532.64  -0.242    0.809    
## plantpl3        178.73     473.62   0.377    0.706    
## LPI:plantpl2    -12.12      36.58  -0.331    0.741    
## LPI:plantpl3    -33.24      34.34  -0.968    0.335    
## ---
## Signif. codes:  0 '***' 0.001 '**' 0.01 '*' 0.05 '.' 0.1 ' ' 1
## 
## (Dispersion parameter for gaussian family taken to be 708260.9)
## 
##     Null deviance: 111235622  on 158  degrees of freedom
## Residual deviance: 107655664  on 152  degrees of freedom
##   (417 observations deleted due to missingness)
## AIC: 2601.9
## 
## Number of Fisher Scoring iterations: 2
```

```
aov.vol <- anova(glmvol,test="Chisq")
kable(aov.vol) # Sign: non
```

	Df	Deviance	Resid. Df	Resid. Dev	Pr(>Chi)
NULL	NA	NA	158	111235622	NA
day	1	247095.924	157	110988526	0.5547495
LPI	1	3317.049	156	110985209	0.9454392
plant	2	2644704.792	154	108340505	0.1545804
LPI:plant	2	684841.000	152	107655664	0.6166420

No effect of environmental factor on spore volume.

Experimental data effect on sporulation rate

```
# Distribution
hist(results$sporulation_rate, col='blue')
```

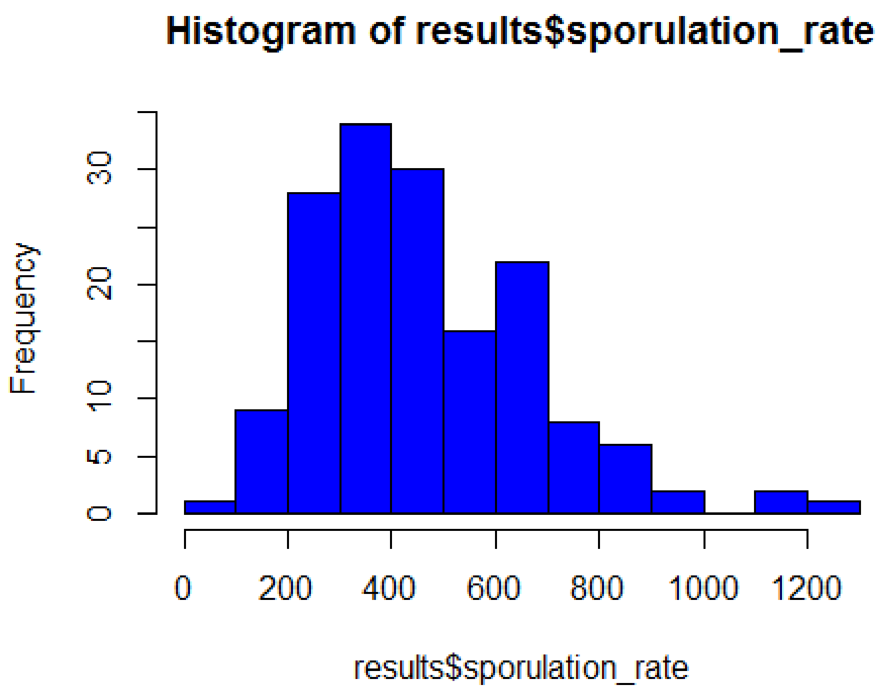


```
# Sporulation rate per LPI
graphe=with(resna,bargraph.CI(x.factor=LPI,response=sporulation_rate,fun=me
an,xlab="LPI",ylab="Sporulation rate (urediniospore/day)",main="Sporulation
rate for each leaf maturity", ylim=c(0,1000), col=cc, xpd=T,legend = T, lc=
T,uc=T, ci.fun= function(x) c(mean(x)-se(x), mean(x) + se(x))))
text(graphe$xvals,graphe$vals+300,c("c","c","c","bc","abc","ab","ab","a"))
# add Tukey coefficients
```

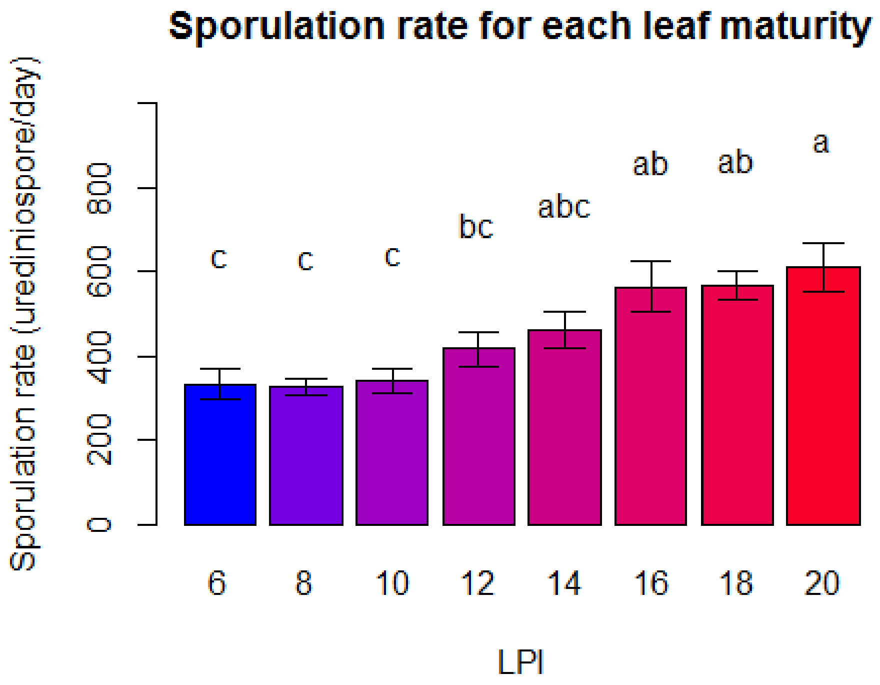


```
# Sporulation rate per plant
graphe=with(resna,bargraph.CI(x.factor=plant,response=sporulation_rate,fun=
mean,xlab="Plant",ylab="Sporulation rate (urediniospore/uredinia/day)",main
="Sporulation rate for each plant",ylim=c(0,1000),col='grey',xpd=T,legend =
T, lc=T,uc=T, ci.fun= function(x) c(mean(x)-se(x), mean(x) + se(x))))
text(graphe$xvals,graphe$vals+300,c("ab","b","a")) # add Tukey coefficients
```

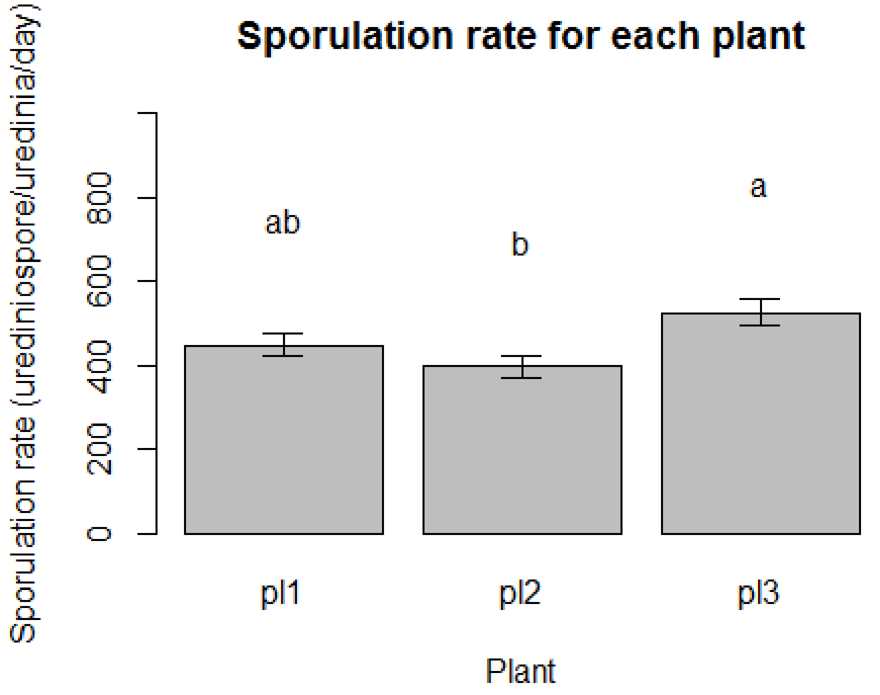

```r
# Sporulation rate per inoculation day
graphe=with(resna,bargraph.CI(x.factor=day,response=sporulation_rate,fun=me
an,xlab="Day",ylab="Sporulation rate (urediniospore/uredinia/day)",main="Sp
orulation rate for each inoculation day",ylim=c(0,1000),col='grey',xpd=T,le
gend = T, lc=T,uc=T, ci.fun= function(x) c(mean(x)-se(x), mean(x) + se(x)))
)
```

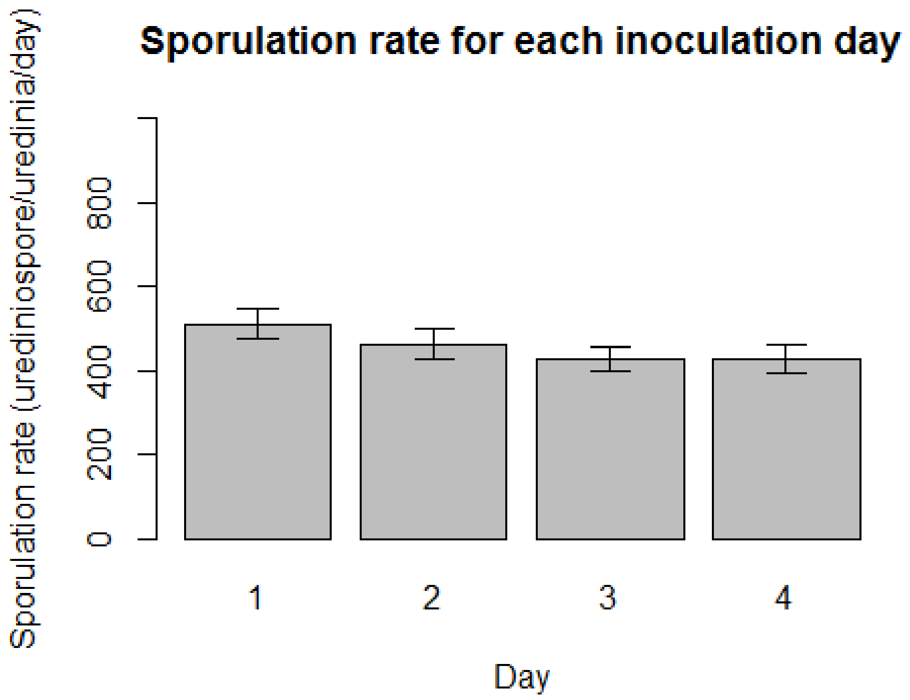

GLM

```r
glmrate = glm(sqrt(sporulation_rate) ~ day+LPI*plant, results, family = gau
ssian)
plot(glmrate, ask=F)
```

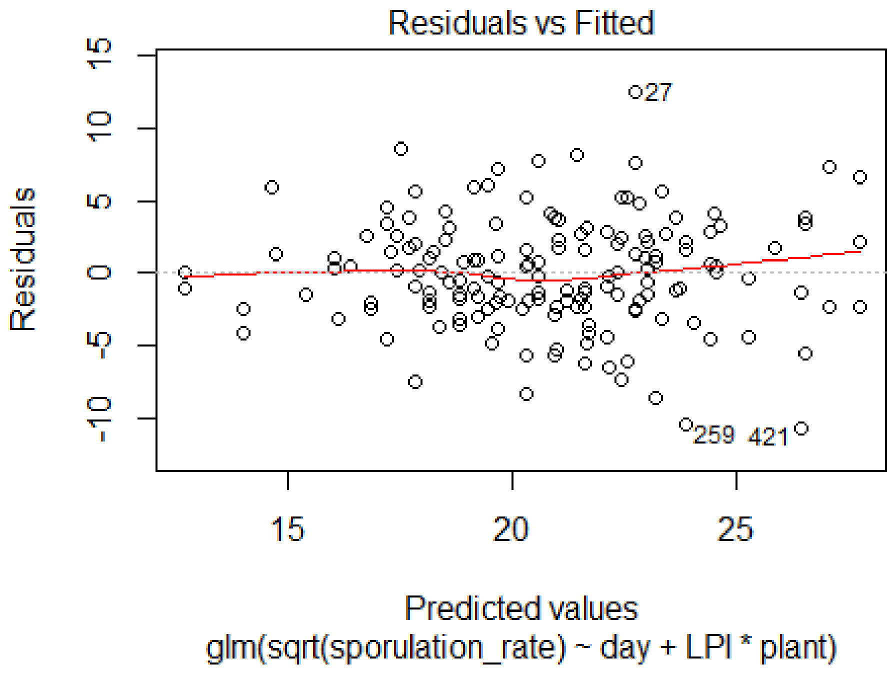
Residuals vs Fitted
Residuals
15
10
5
0
-5
-10
27
259
421
15
20
25
Predicted values
glm(sqrt(sporulation_rate) ~ day + LPI * plant)

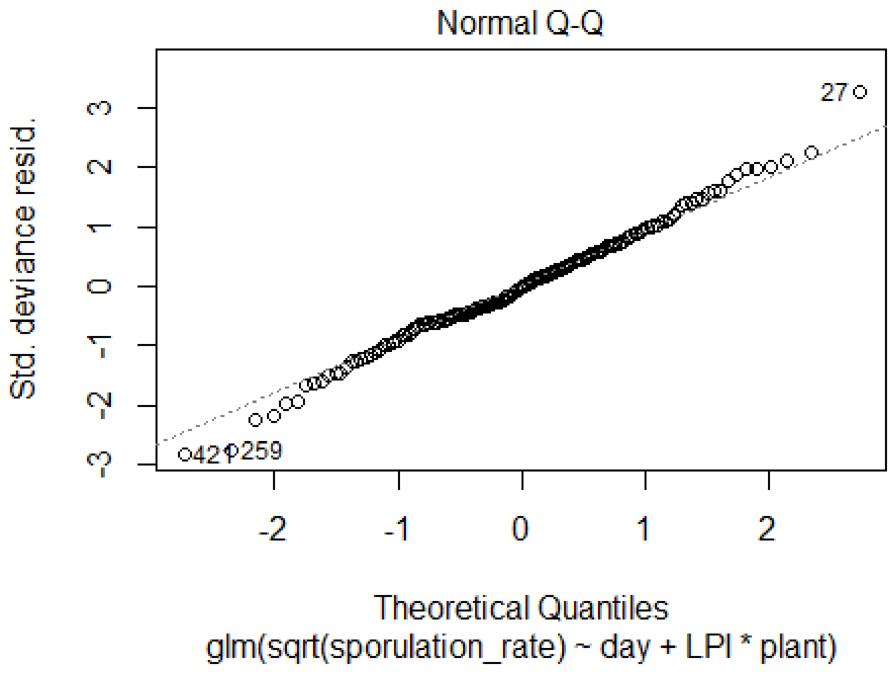
Normal Q-Q
Std. deviance resid.
3
2
1
0
-1
-2
-3
27
421
259
-2
-1
0
1
2
Theoretical Quantiles
glm(sqrt(sporulation_rate) ~ day + LPI * plant)

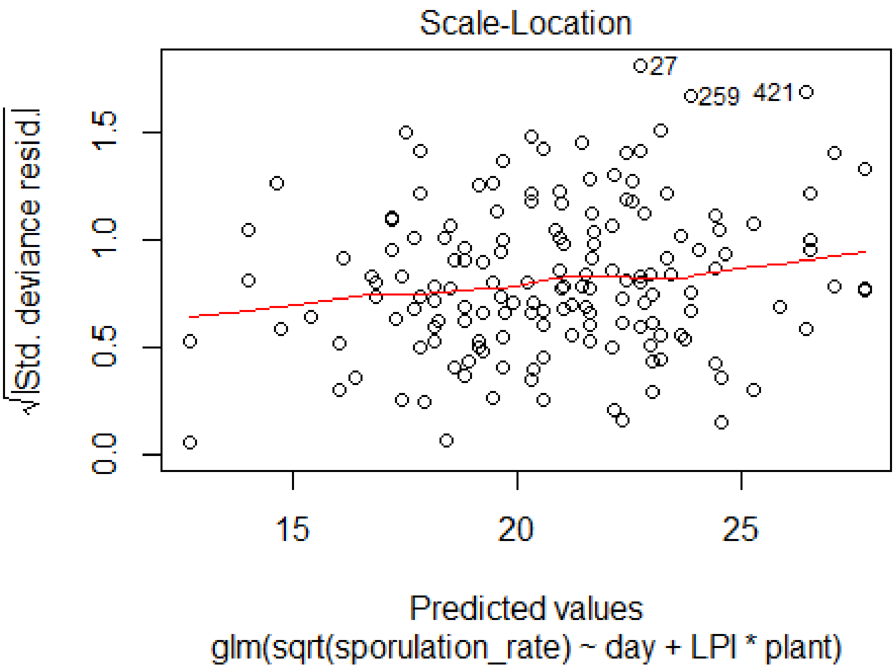

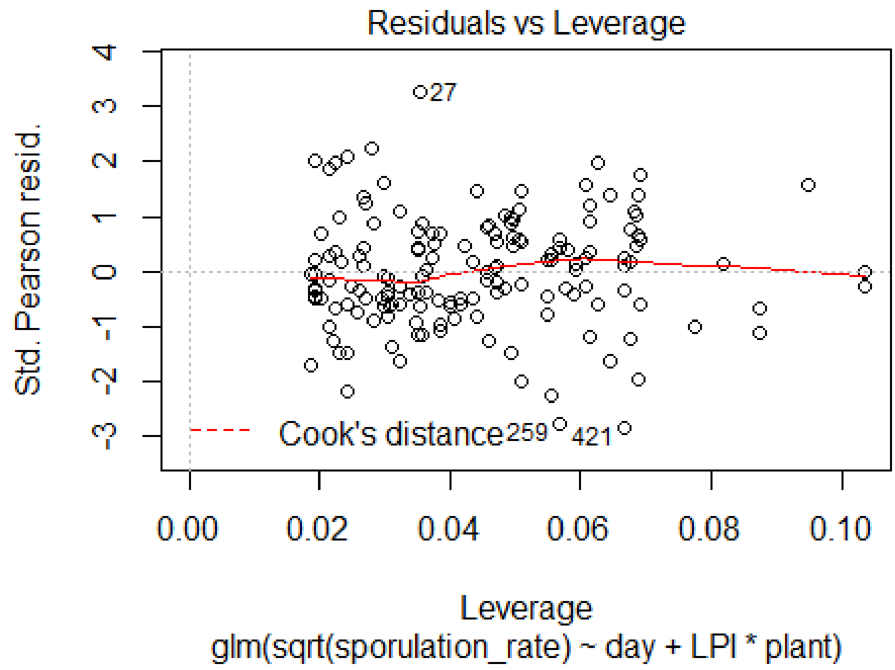


```
summary(glmrate) # Sign : Intercept, LPI, day

##
## Call:
## glm(formula = sqrt(sporulation_rate) ~ day + LPI * plant, family = gauss
ian,
##     data = results)
```

```
##
## Deviance Residuals:
##      Min        1Q    Median        3Q       Max
## -10.6398   -2.2896    0.0128    2.3649   12.4697
##
## Coefficients:
##               Estimate Std. Error t value Pr(>|t|)
## (Intercept)   16.3902     1.7623   9.301  < 2e-16 ***
## day           -0.6538     0.2796  -2.339 0.020653 *
## LPI            0.4390     0.1155   3.800 0.000209 ***
## plantpl2      -5.2921     2.4616  -2.150 0.033144 *
## plantpl3      -0.2790     2.1888  -0.127 0.898732
## LPI:plantpl2   0.2595     0.1690   1.535 0.126798
## LPI:plantpl3   0.1760     0.1587   1.109 0.269095
## ---
## Signif. codes:  0 '***' 0.001 '**' 0.01 '*' 0.05 '.' 0.1 ' ' 1
##
## (Dispersion parameter for gaussian family taken to be 15.12695)
##
##     Null deviance: 3778.7  on 158  degrees of freedom
## Residual deviance: 2299.3  on 152  degrees of freedom
##   (417 observations deleted due to missingness)
## AIC: 891.98
##
## Number of Fisher Scoring iterations: 2
```

```
aov.rate <- anova(glmrate,test="Chisq")
kable(aov.rate) # LPI***, plant***, day*
```

	Df	Deviance	Resid. Df	Resid. Dev	Pr(>Chi)
NULL	NA	NA	158	3778.730	NA
day	1	87.98223	157	3690.748	0.0158787
LPI	1	992.56956	156	2698.178	0.0000000
plant	2	360.81320	154	2337.365	0.0000066
LPI:plant	2	38.06786	152	2299.297	0.2841426

```
Tukey_LPI <- HSD.test(glmrate,"LPI",group=TRUE,main="Sporulation rate for e
ach LPI")
Tukey_plant <- HSD.test(glmrate,"plant",group=TRUE,main="Sporulation rate f
or each plant")
Tukey_day <- HSD.test(glmrate,"day",group=TRUE,main="Sporulation rate for e
ach day")
kable(Tukey_LPI$groups)
```

	sqrt(sporulation_rate)	groups
20	24.06006	a

18	23.60081	ab
16	23.28814	ab
14	21.12870	abc
12	19.95987	bc
8	17.99068	c
10	17.96135	c
6	17.67110	c

```r
kable(Tukey_plant$groups)
```

	sqrt(sporulation_rate)	groups
pl3	22.21492	a
pl1	20.69748	ab
pl2	19.39211	b

```r
kable(Tukey_day$groups)
```

sqrt(sporulation_rate)	groups
22.04345	a
20.77353	a
20.18230	a
20.12319	a

LPI and plant have significant effects on sporulation rate.

Experimental data effect on sporulation capacity

```r
# Distribution
hist(results$sporulation_capacity, col='blue')
```

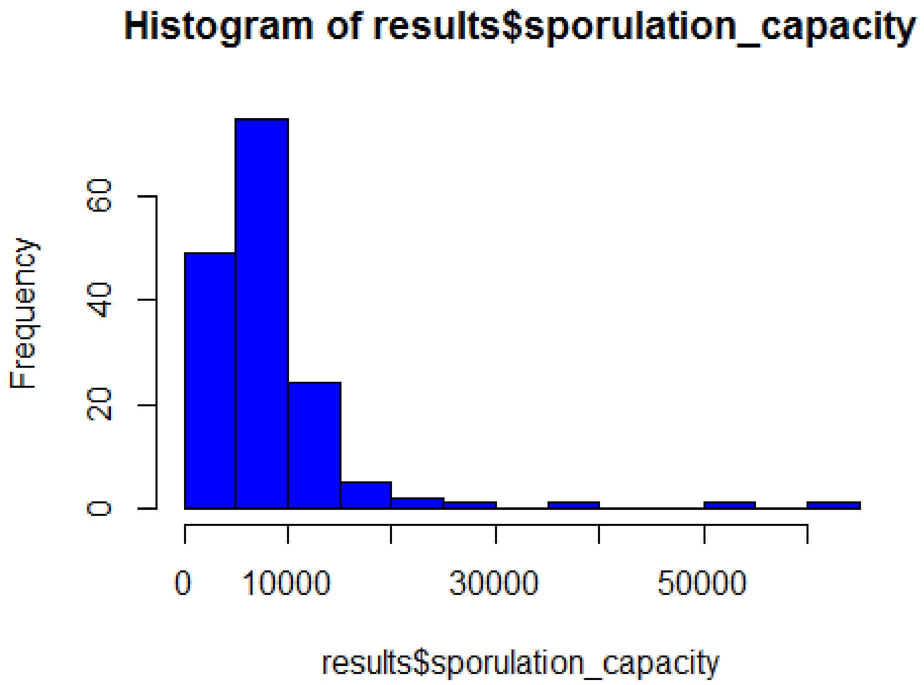


```
# Sporulation capacity per LPI
graphe=with(resna,bargraph.CI(x.factor=LPI,response=sporulation_capacity,fu
n=mean,xlab="LPI",ylab="Sporulation capacity (urediniospore/µm2/day)",main=
"Sporulation capacity for each leaf maturity", ylim=c(0,20000), col=cc, xpd
=T,legend = T, lc=T,uc=T, ci.fun= function(x) c(mean(x)-se(x), mean(x) + se
(x))))
text(graphe$xvals,graphe$vals+5000,c("a","b","c","f","d","e","h","g")) # ad
d Tukey coefficients
```

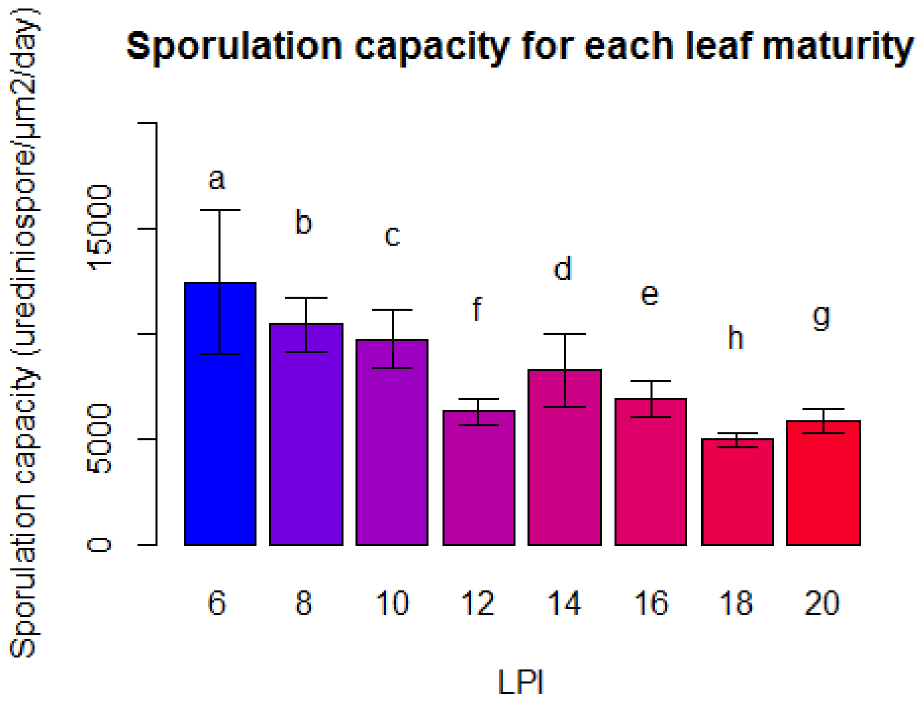


```
# Sporulation capacity per plant
graphe=with(resna,bargraph.CI(x.factor=plant,response=sporulation_capacity,
fun=mean,xlab="Plant",ylab="Sporulation capacity (urediniospore/µm2/day)",m
ain="Sporulation capacity for each plant",ylim=c(0,15000),col='grey',xpd=T,
legend = T, lc=T,uc=T, ci.fun= function(x) c(mean(x)-se(x), mean(x) + se(x)
)))
```

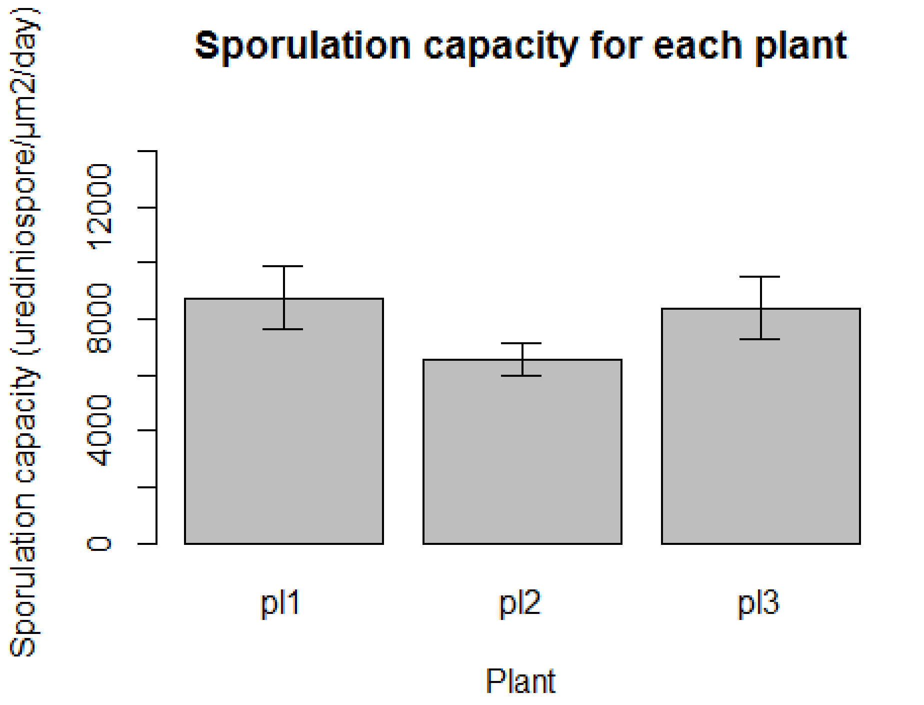

```r
# Sporulation capacity per inoculation day
graphe=with(resna,bargraph.CI(x.factor=day,response=sporulation_capacity,fun=mean,xlab="Day",ylab="Sporulation capacity (urediniospore/µm2/day)",main="Sporulation capacity for each inoculation day",ylim=c(0,15000),col='grey',xpd=T,legend = T, lc=T,uc=T, ci.fun= function(x) c(mean(x)-se(x), mean(x) + se(x))))
```

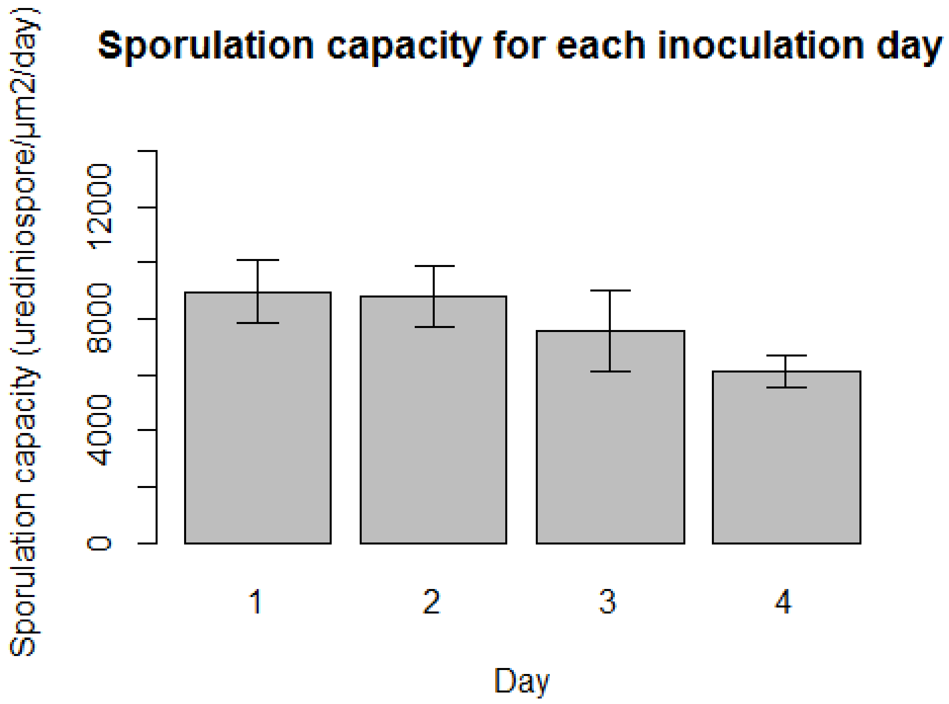

GLM

```r
glmcapa = glm(sporulation_capacity ~ day+LPI*plant, results, family = Gamma(link= inverse))
plot(glmcapa, ask=F)
```

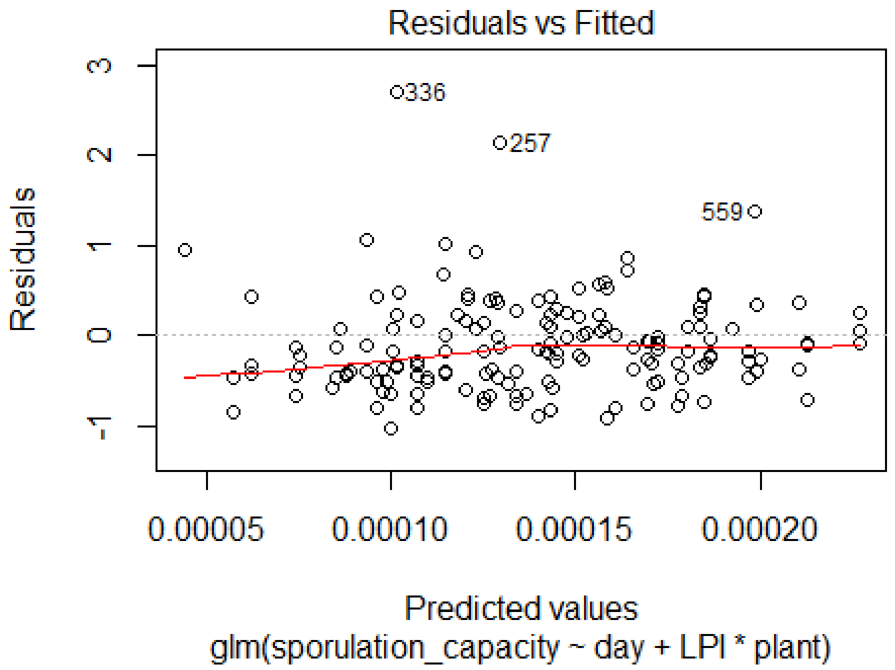
Residuals vs Fitted
Residuals
3
2
1
0
-1
336
257
559
0.00005
0.00010
0.00015
0.00020
Predicted values
glm(sporulation_capacity ~ day + LPI * plant)

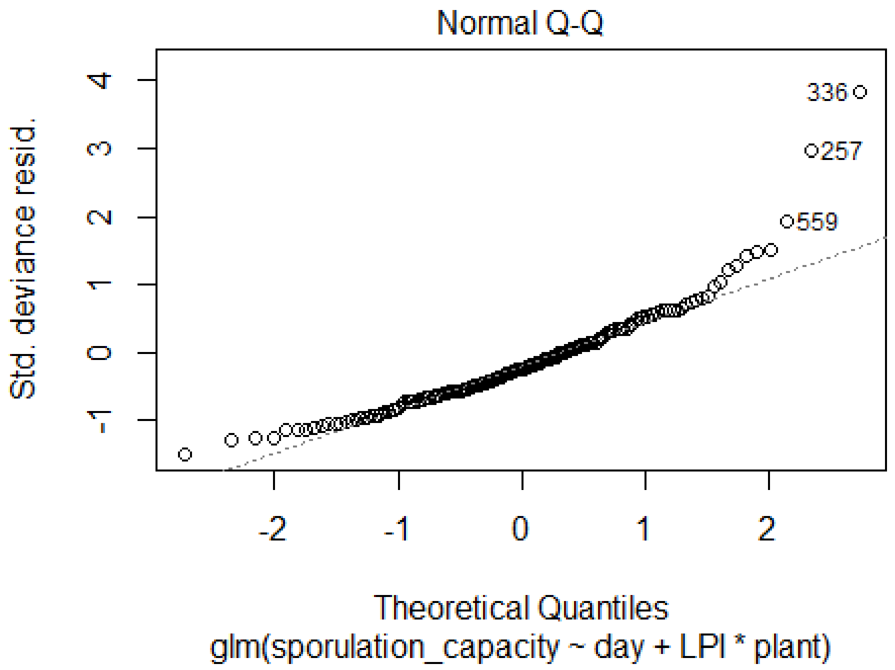
Normal Q-Q
Std. deviance resid.
4
3
2
1
0
-1
336
257
559
-2
-1
0
1
2
Theoretical Quantiles
glm(sporulation_capacity ~ day + LPI * plant)

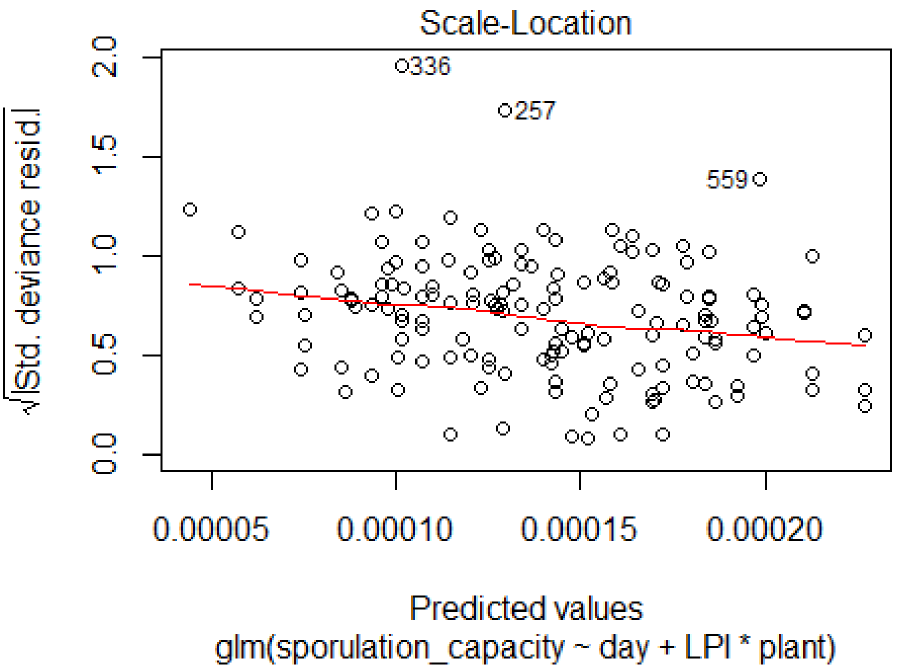

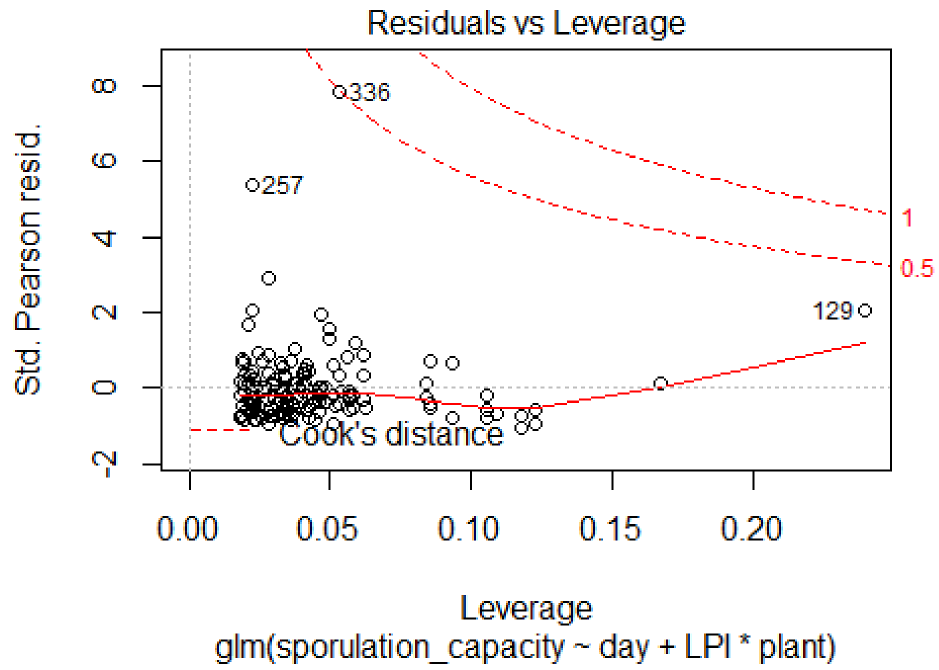


```
summary(glmcapa) # Sign : Intercept, LPI, day

##
## Call:
## glm(formula = sporulation_capacity ~ day + LPI * plant, family = Gamma(l
ink = inverse),
##     data = results)
```

```
##
## Deviance Residuals:
##     Min       1Q   Median       3Q      Max
## -1.0213  -0.4491  -0.1678   0.1673   2.7031
##
## Coefficients:
##                  Estimate Std. Error t value Pr(>|t|)
## (Intercept)  -2.390e-05  2.899e-05  -0.825 0.410944
## day           1.350e-05  6.531e-06   2.067 0.040464 *
## LPI           9.026e-06  2.524e-06   3.576 0.000469 ***
## plantpl2      5.364e-05  5.026e-05   1.067 0.287589
## plantpl3      5.208e-05  3.987e-05   1.306 0.193420
## LPI:plantpl2 -1.864e-06  4.152e-06  -0.449 0.654047
## LPI:plantpl3 -3.580e-06  3.573e-06  -1.002 0.318040
## ---
## Signif. codes:  0 '***' 0.001 '**' 0.01 '*' 0.05 '.' 0.1 ' ' 1
##
## (Dispersion parameter for Gamma family taken to be 0.5271046)
##
##     Null deviance: 63.521  on 158  degrees of freedom
## Residual deviance: 46.222  on 152  degrees of freedom
##   (417 observations deleted due to missingness)
## AIC: 3070.7
##
## Number of Fisher Scoring iterations: 7
```

```
aov.capa <- anova(glmcapa,test="Chisq")
kable(aov.capa) # LPI***
```

	Df	Deviance	Resid. Df	Resid. Dev	Pr(>Chi)
NULL	NA	NA	158	63.52131	NA
day	1	2.7419613	157	60.77935	0.0225618
LPI	1	12.1986333	156	48.58071	0.0000015
plant	2	1.8302893	154	46.75043	0.1761935
LPI:plant	2	0.5286968	152	46.22173	0.6056153

```
Tukey_LPI <- HSD.test(glmcapa,"LPI",group=TRUE,main="Sporulation capacity f
or each LPI")
kable(Tukey_LPI$groups)
```

	sporulation_capacity	groups
6	12423.577	a
8	10445.532	b
10	9525.264	c
14	8255.382	d

16	6888.095	e
12	6300.009	f
20	5808.520	g
18	4933.928	h

LPI has significant effects on sporulation capacity.

Experimental data effect on mycelium quantity

```
# Distribution
hist(results$mycelium_quantity, col='blue')
```

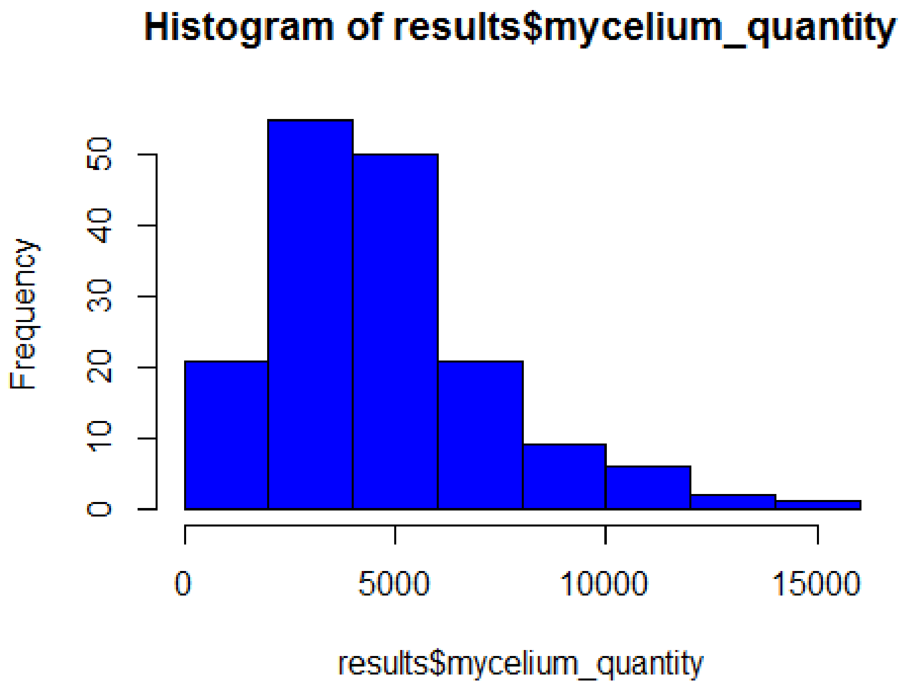


```
# Mycelium quantity per LPI
graphe=with(resna,bargraph.CI(x.factor=LPI,response=mycelium_quantity,fun=m
ean,xlab="LPI",ylab="Mycelium quantity (arbitrary unit)",main="Mycelium qua
ntity for each leaf maturity",col=cc, xpd=T,legend = T, lc=T,uc=T, ci.fun=
function(x) c(mean(x)-se(x), mean(x) + se(x))))
```

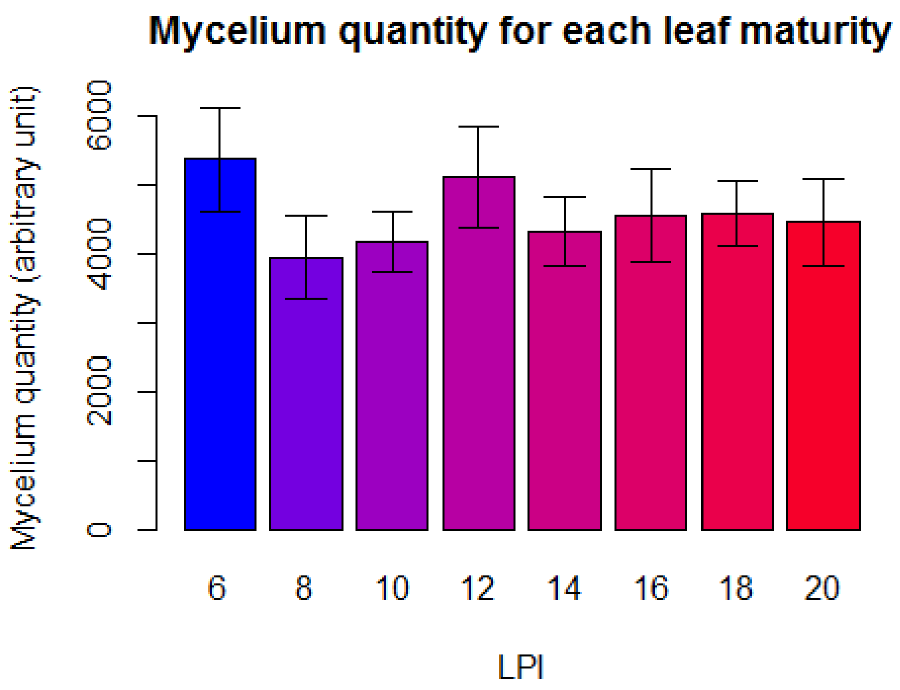


```
# Mycelium quantity per plant
graphe=with(resna,bargraph.CI(x.factor=plant,response=mycelium_quantity,fun
=mean,xlab="Plant",ylab="Mycelium quantity (arbitrary unit)",main="Mycelium
quantity for each plant",col='grey', xpd=T,legend = T, lc=T,uc=T, ci.fun= f
unction(x) c(mean(x)-se(x), mean(x) + se(x))))
```

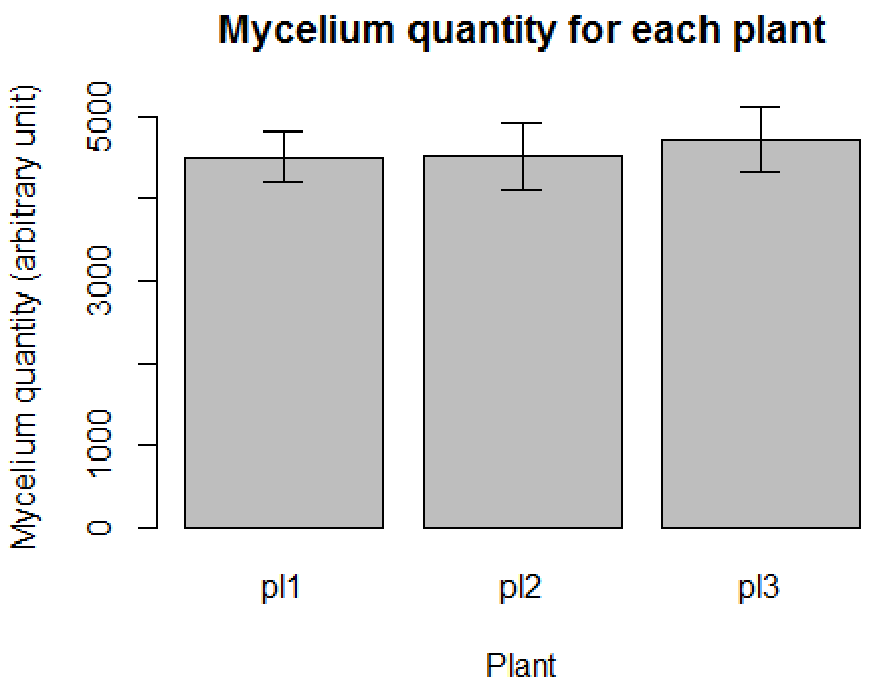

```
# Mycelium quantity per inoculation day
graphe=with(resna,bargraph.CI(x.factor=day,response=mycelium_quantity,fun=m
ean,xlab="Day",ylab="Mycelium quantity (arbitrary unit)",main="Mycelium qua
ntity for each inoculation day",ylim=c(0,6500),col='grey',xpd=T,legend = T,
lc=T,uc=T, ci.fun= function(x) c(mean(x)-se(x), mean(x) + se(x))))
```

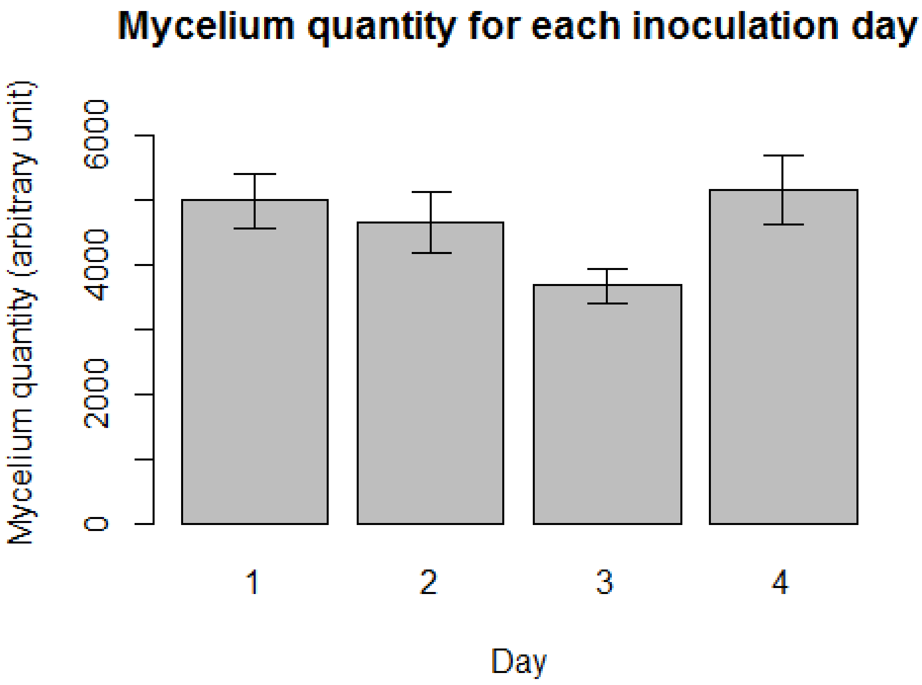

GLM

```
glmmyc <- glm(sqrt(mycelium_quantity) ~ day+LPI*plant, results, family = ga
ussian)
plot(glmmyc, ask=F)
```

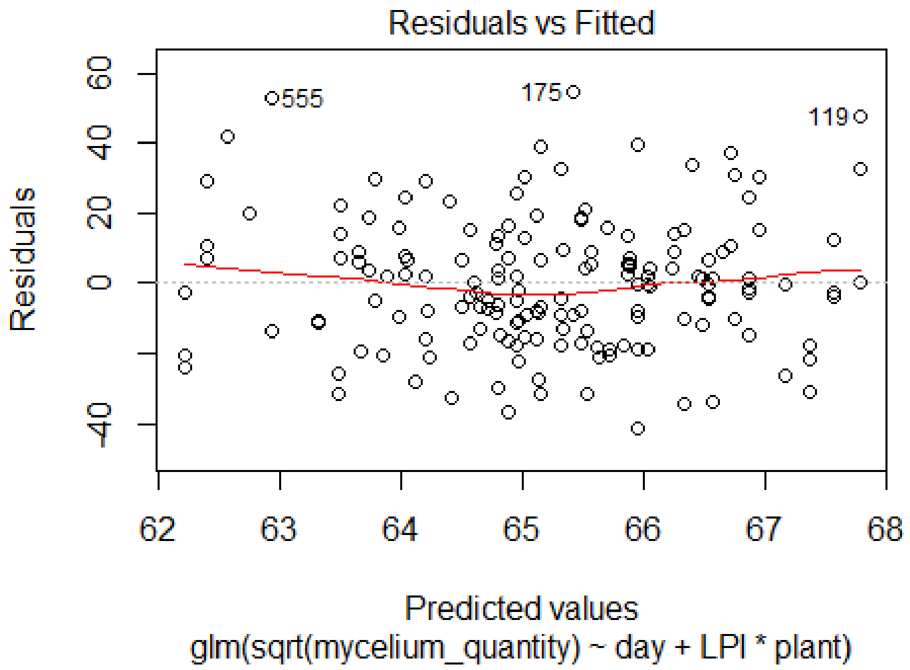
Residuals vs Fitted
Residuals
60
40
20
0
-40
555
175
119
62
63
64
65
66
67
68
Predicted values
glm(sqrt(mycelium_quantity) ~ day + LPI * plant)

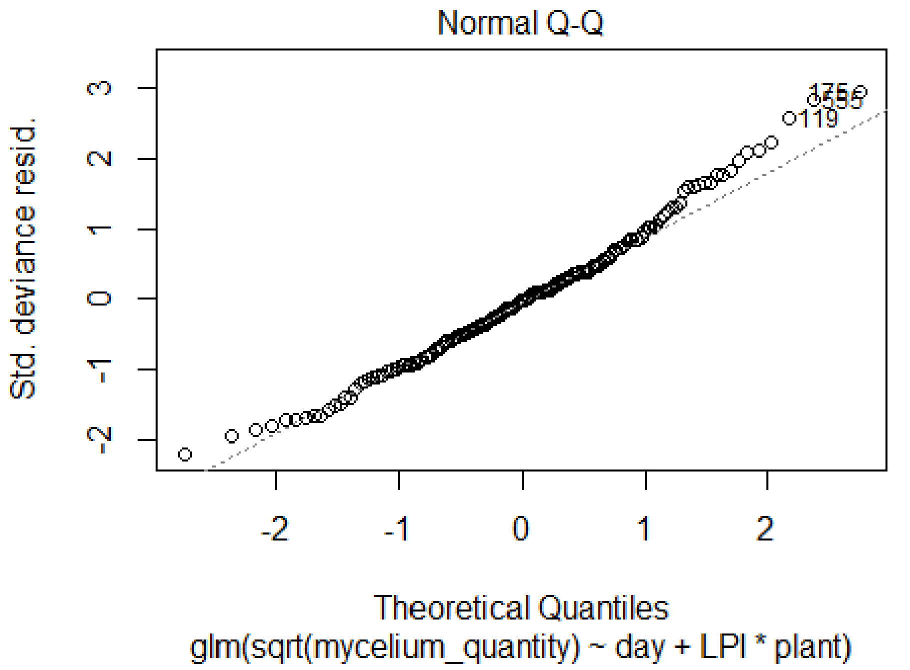
Normal Q-Q
Std. deviance resid.
3
2
1
0
-1
-2
175
555
119
-2
-1
0
1
2
Theoretical Quantiles
glm(sqrt(mycelium_quantity) ~ day + LPI * plant)

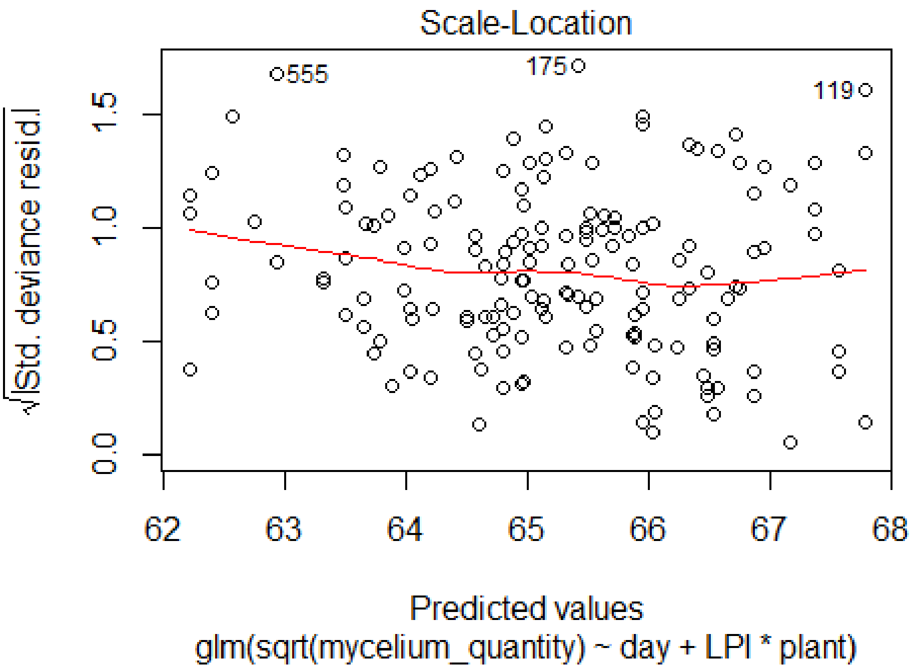

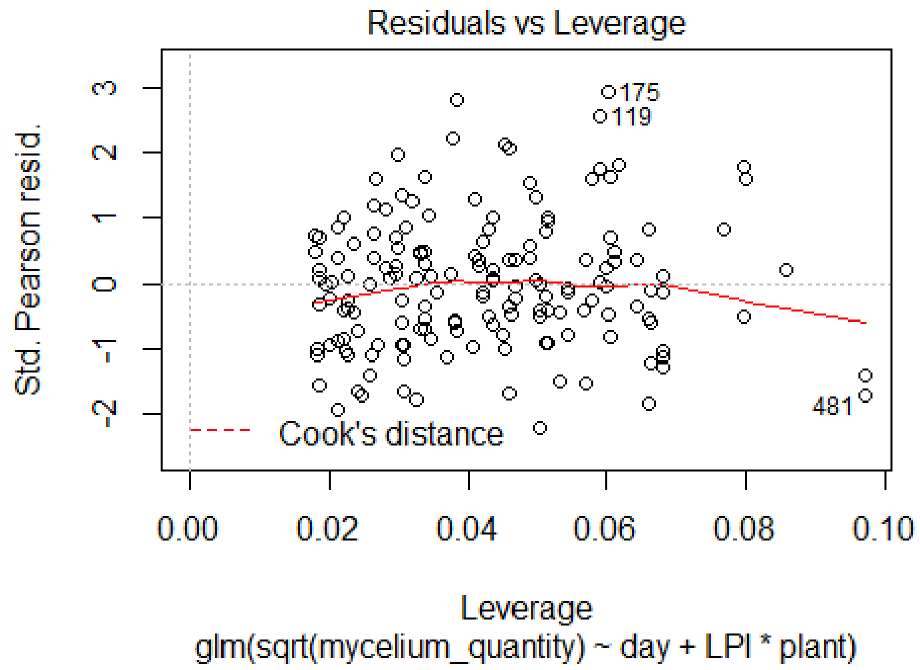


```
summary(glmmyc) # Sign: day

##
## Call:
## glm(formula = sqrt(mycelium_quantity) ~ day + LPI * plant, family = gaus
sian,
##     data = results)
```

```
##
## Deviance Residuals:
##     Min       1Q   Median       3Q      Max
## -41.183  -13.137   -0.362   10.414   54.538
##
## Coefficients:
##               Estimate Std. Error t value Pr(>|t|)
## (Intercept)  68.09934    8.60147   7.917 4.03e-13 ***
## day          -0.91860    1.34491  -0.683    0.496
## LPI          -0.03875    0.55413  -0.070    0.944
## plantpl2     -0.39421   11.80883  -0.033    0.973
## plantpl3      1.21987   10.64487   0.115    0.909
## LPI:plantpl2 -0.05226    0.81174  -0.064    0.949
## LPI:plantpl3 -0.06455    0.76410  -0.084    0.933
## ---
## Signif. codes:  0 '***' 0.001 '**' 0.01 '*' 0.05 '.' 0.1 ' ' 1
##
## (Dispersion parameter for gaussian family taken to be 366.9218)
##
##     Null deviance: 58238  on 164  degrees of freedom
## Residual deviance: 57974  on 158  degrees of freedom
##   (411 observations deleted due to missingness)
## AIC: 1451.4
##
## Number of Fisher Scoring iterations: 2
```

```
aov.myc <- anova(glmmyc,test="Chisq")
kable(aov.myc) # Sign: none
```

	Df	Deviance	Resid. Df	Resid. Dev	Pr(>Chi)
NULL	NA	NA	164	58238.35	NA
day	1	169.736862	163	58068.61	0.4964128
LPI	1	30.506287	162	58038.11	0.7730850
plant	2	61.593745	160	57976.51	0.9194928
LPI:plant	2	2.868327	158	57973.65	0.9960990

No effect of environmental factors on in planta mycelium quantity.

Summary of anova coefficients

```
aov.table <- matrix(nrow = 7, ncol=4,'NA')
rownames(aov.table) <- c('Infection efficiency','Latent period','Uredinia s
ize','Spore volume','Sporulation rate','Sporulation capacity', 'Mycelium qu
antity')
colnames(aov.table) <- c('LPI','Plant','LPI:Plant','day')
```

```r
aov.table['Infection efficiency',1:4] <- round(aov.eff[c('LPI','plant', 'LPI:plant', 'day'),'Pr(>Chi)'], 3)
aov.table['Latent period',1:4] <- round(aov.lat[c('LPI','plant', 'LPI:plant', 'day'),'Pr(>Chi)'], 3)
aov.table['Uredinia size',1:4] <- round(aov.us[c('LPI','plant', 'LPI:plant', 'day'),'Pr(>Chi)'], 3)
aov.table['Spore volume',1:4] <- round(aov.vol[c('LPI','plant', 'LPI:plant', 'day'),'Pr(>Chi)'], 3)
aov.table['Sporulation rate',1:4] <- round(aov.rate[c('LPI','plant', 'LPI:plant', 'day'),'Pr(>Chi)'], 3)
aov.table['Sporulation capacity',1:4] <- round(aov.capa[c('LPI','plant', 'LPI:plant', 'day'),'Pr(>Chi)'], 3)
aov.table['Mycelium quantity',1:4] <- round(aov.myc[c('LPI','plant', 'LPI:plant', 'day'),'Pr(>Chi)'], 3)


kable(aov.table)
```

	LPI	Plant	LPI:Plant	day
Infection efficiency	0.246	0.416	0.136	0.05
Latent period	0.578	0.053	0.535	0.425
Uredinia size	0	0.023	0.229	0.136
Spore volume	0.945	0.155	0.617	0.555
Sporulation rate	0	0	0.284	0.016
Sporulation capacity	0	0.176	0.606	0.023
Mycelium quantity	0.773	0.919	0.996	0.496

```r
# FDR correction for multiple tests
aov.adj <- p.adjust(aov.table, method='fdr')
aov.adj.table <- matrix(data = aov.adj, nrow = 7, ncol = 4)

colnames(aov.adj.table) <- colnames(aov.table)
rownames(aov.adj.table) <- rownames(aov.table)
kable(round(aov.adj.table, 3))
```

	LPI	Plant	LPI:Plant	day
Infection efficiency	0.459	0.661	0.346	0.165
Latent period	0.720	0.165	0.720	0.661
Uredinia size	0.000	0.092	0.458	0.346
Spore volume	0.980	0.362	0.720	0.720
Sporulation rate	0.000	0.000	0.497	0.090
Sporulation capacity	0.000	0.379	0.720	0.092
Mycelium quantity	0.866	0.980	0.996	0.720

Leaf characteristics data exploration: PCA

Describe the raw leaf characteristics

```
# Dataset
leaf <- merge(sugar, phenol, by='leaf_id')
leaf <- merge(leaf, phenolics, by='leaf_id')
leaf <- merge(leaf, CN, by='leaf_id')
leaf$total_phenolics <- leaf$polyphenols
leaf$LPI <- leaf$LPI.x
leaf$plant <- leaf$plant.x

#PCA

# pca <- dudi.pca(leaf[,c('Chlorogenic_acid','PA_dimer','Salicine','Caffeoy
l_glucose_isomer1','Catechin','Coumaroyl_glucose_isomer1','Caffeoyl_glucose
_isomer2','Coumaroyl_glucose_isomer2','Caffeoyl_shikimate','Salicortin','Ru
tin','AcetylSalicortin','Quercitrin','Homaloside_D','Putative_populoside','
Tremulacin', 'sucrose', 'glucose','fructose','C','N')], center=T, scale=T,
nf=3,scannf=FALSE)
# OR
pca <- dudi.pca(leaf[,c('salicinoid', 'flavonol', 'hydroxycinnamic_acid_est
ers', 'flavan3ol','total_phenolics', 'sucrose','glucose','fructose','C','N'
)], center=T, scale=T, nf=3,scannf=FALSE)
sup <- supcol(pca,scalewt(leaf[c('LPI','plant')]))$cosup
p <- fviz_pca_var(pca,
            col.var = "contrib", # Color by contributions to the PC
            gradient.cols = c("#00AFBB","#FC4E07"),
            repel = TRUE)     # Avoid text overlapping
# Add experimental (LPI, plant and day) data as supplementary variables
fviz_add(p, sup, geom="arrow", linetype='solid', color ="black")
```

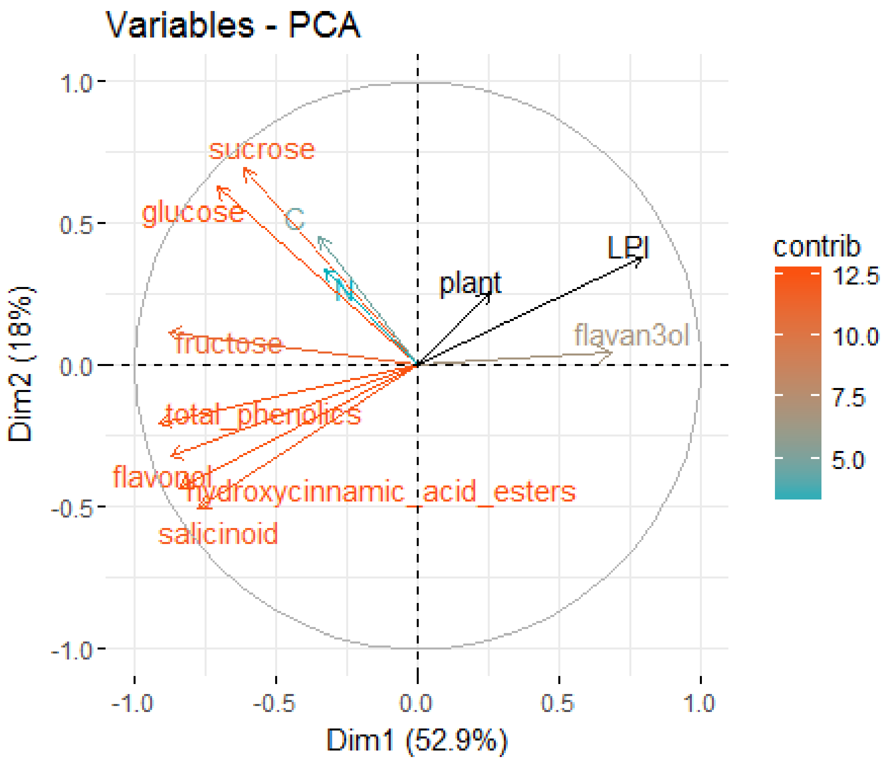


```
# color scale for LPI representation on 1st and 2nd axis
cc <- scales::seq_gradient_pal("blue", "red", "Lab")(seq(0,1,length.out=8))
s.class(dfxy = pca$li, fac = as.factor(leaf$LPI),col = cc, clabel = 0.9, gr
id= 0, xax = 1, yax = 2, add.plot= F, cellipse = F)
```

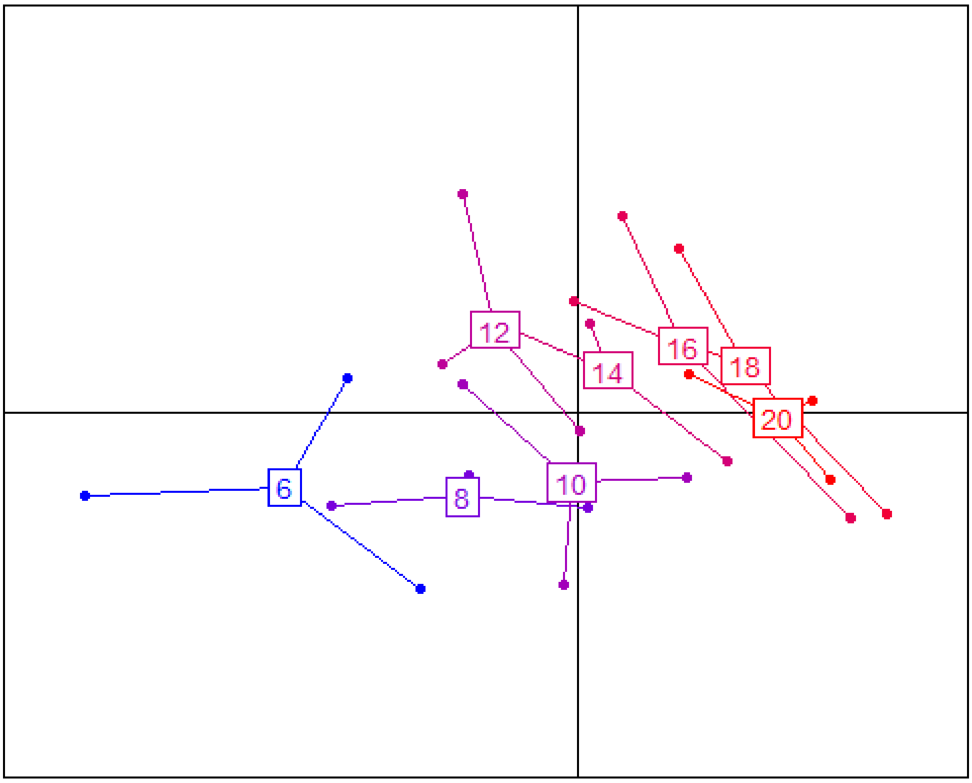

```r
# PCA representation of 1st and 3rd axis
fviz_pca_var(pca, axes = c(2, 3),
             col.var = "contrib", # Color by contributions to the PC
             gradient.cols = c("#00AFBB","#FC4E07"),
             repel = TRUE)      # Avoid text overlapping)
```

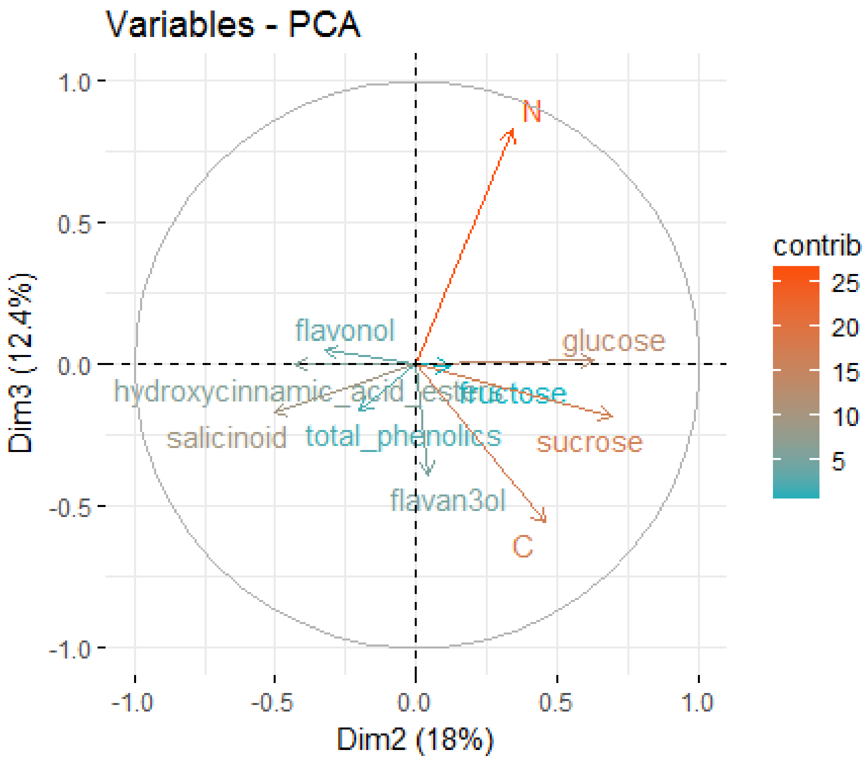


```r
# PCA variances explained on the 3 first axis
pca.var<-data.frame(pca$co)
kable(pca.var)
```

	Comp1	Comp2	Comp3
salicinoid	-0.7762155	-0.5066011	-0.1709359
flavonol	-0.8752044	-0.3217504	0.0513348
hydroxycinnamic_acid_esters	-0.8427070	-0.4295869	0.0006166
flavan3ol	0.6857073	0.0424990	-0.3891061
total_phenolics	-0.9172491	-0.2029745	-0.1639736
sucrose	-0.6179250	0.6931257	-0.1816724
glucose	-0.7108893	0.6289603	0.0154362
fructose	-0.8805084	0.1174024	-0.0073086

C	-0.3524496	0.4545417	-0.5561184
N	-0.3311749	0.3417146	0.8290247
PC1 is related to all phenolic	compounds an	d explains al	most 53% of the total inertia. Sugars variation is not depending on LPI.
LPI can be well discriminated	according to	their metabol	ite content (especially through total phenolics and 3 phenolic subclasses, flavonols, salicinoids and hydroxycinnamic acid esters).

Heatmap

```
leaf_hm <- as.matrix(leaf[,c('sucrose','glucose','fructose','total_phenolics','C','N')])
leaf_hm <- as.matrix(leaf[,c('sucrose','glucose','fructose','Chlorogenic_acid','PA_dimer','Salicine','Caffeoyl_glucose_isomer1','Catechin','Coumaroyl_glucose_isomer1','Caffeoyl_glucose_isomer2','Coumaroyl_glucose_isomer2','Caffeoyl_shikimate','Salicortin','Rutin','AcetylSalicortin','Quercitrin','Homaloside_D','Putative_populoside','Tremulacin','C','N')])
rownames(leaf_hm) <- leaf$leaf_id

hm <- heatmap(leaf_hm, xlab="Leaf characteristics", ylab = "LPI", scale='col',col=cc)
```

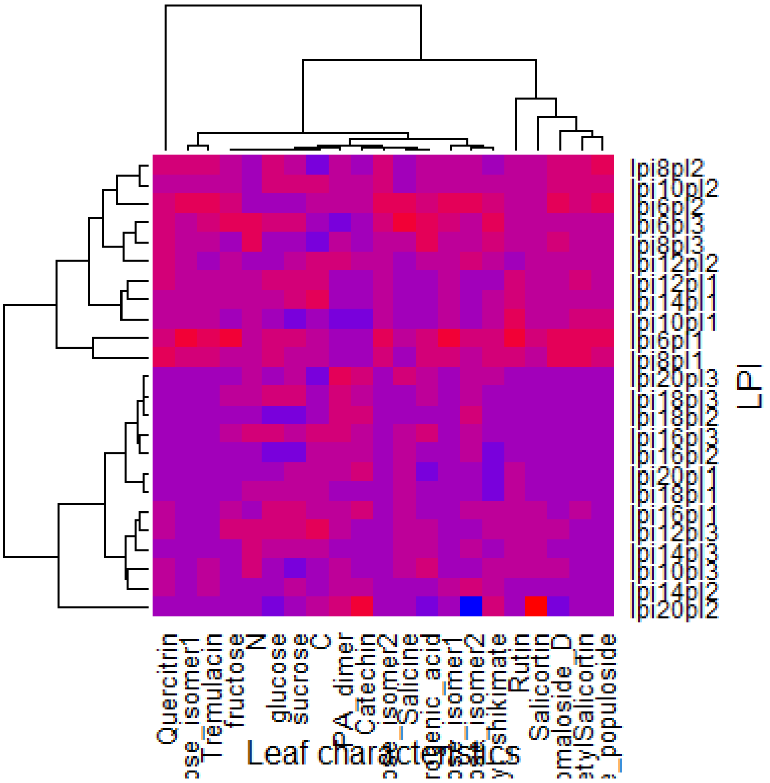

Test for leaf data: on 24 observations (3 plants x 8 LPI)

Test the correlation between leaf data and fungal data. On agregated fungal data per LPI and plant to fit to the leaf data's scale.

```
# Aggregate fungal data
resag <- aggregate(results[c('infection_efficiency','latent_period', 'uredi
nia_size', 'spore_volume', 'sporulation_rate', 'sporulation_capacity', 'myc
elium_quantity', 'sucrose', 'glucose', 'fructose', 'C', 'N', 'total_phenoli
cs', 'salicinoid', 'flavonol', 'hydroxycinnamic_acid_esters', 'flavan3ol')]
,results[c('leaf_id')], function(a) c(mean(a, na.rm=T)))

# Correlation matrix (on ordered variables)
#resagO <- resag[,c('leaf_id','C','N','sucrose','glucose','fructose','salic
in', 'total_phenolics', 'mycelium_quantity', 'uredinia_size','sporulation_r
ate', 'sporulation_capacity', 'spore_volume', 'infection_efficiency', 'late
nt_period')]
#mat <- rcorr(as.matrix(resagO[,2:ncol(resagO)]), type="spearman") # Spearm
an test because nonparametric variables
mat <- rcorr(as.matrix(resag[,2:ncol(resag)]), type="spearman") # Spearman
test because nonparametric variables
corrplot(mat$r, p.mat=mat$P, sig.level =1.5e-03, insig = "label_sig", pch.c
ol = 'black', type='upper', order='original', tl.col='black', tl.srt=45, di
ag=F, title='Plot of Spearman correlation coefficients') # order = 'hclust'
ie for the hierarchical clustering order OR 'original' ie as in the table
```

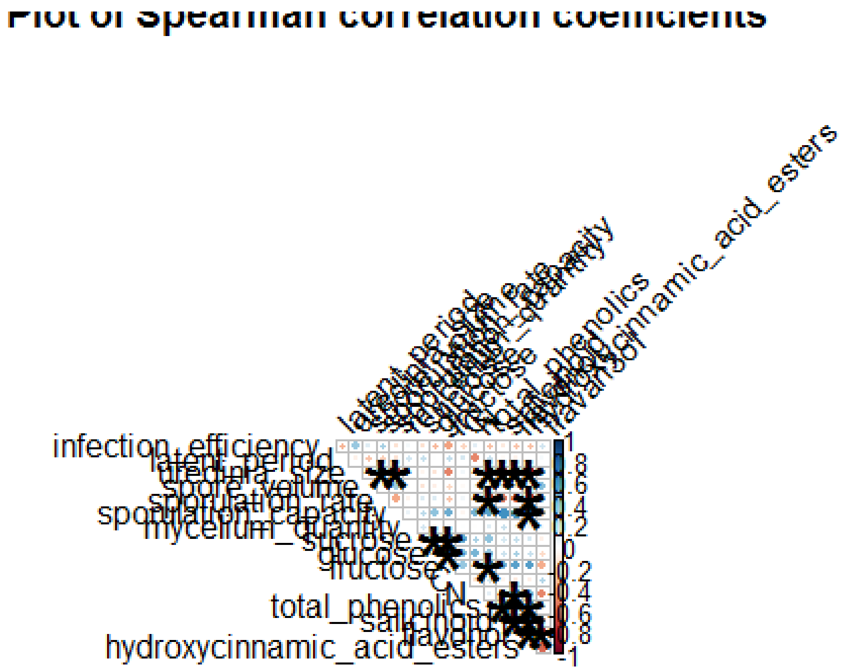

blue = positive correlations
red = negative correlations
dot size = absolute value

```
# Correction of Pvalues for multiple tests : False Discovery Rate: to fix s
ig.level in corrplot
# flatten matrices of correlation coefficients and Pvalues
flattenCorrMatrix <- function(cormat, pmat) {
  ut <- upper.tri(cormat)
  data.frame(
    row = rownames(cormat)[row(cormat)[ut]],
    column = rownames(cormat)[col(cormat)[ut]],
    cor  =(cormat)[ut],
    p = pmat[ut]
    )
}
flatmat <- flattenCorrMatrix(cormat=mat$r, pmat=mat$P)
print(flatmat[1:5,])
```

```
##                     row       column         cor          p
## 1 infection_efficiency latent_period -0.34411128 0.09965466
## 2 infection_efficiency uredinia_size  0.37673367 0.06958357
## 3        latent_period uredinia_size -0.07091583 0.74194120
## 4 infection_efficiency  spore_volume  0.13943168 0.51582531
## 5        latent_period  spore_volume -0.28061785 0.18410347
```

```
# Calculate the adjusted Pvalues
flatmat$adjusted <- p.adjust(flatmat$p, method='fdr', n=length(flatmat$p))
# Display significant correlations
flatmat[which(flatmat$adjusted < 0.01),]
```

```
##                      row                     column        cor
## 9          uredinia_size          sporulation_rate  0.8686957
## 13         uredinia_size      sporulation_capacity -0.7330435
## 36               sucrose                   glucose  0.8469565
## 44               sucrose                  fructose  0.7765217
## 45               glucose                  fructose  0.8200000
## 69         uredinia_size           total_phenolics -0.7666884
## 71      sporulation_rate           total_phenolics -0.6140465
## 76              fructose           total_phenolics  0.6997173
## 81         uredinia_size                salicinoid -0.7252174
## 91       total_phenolics                salicinoid  0.7184171
## 94         uredinia_size                  flavonol -0.6713043
## 103                    N                  flavonol  0.6504348
## 105           salicinoid                  flavonol  0.7626087
## 108        uredinia_size hydroxycinnamic_acid_esters -0.8260870
## 110     sporulation_rate hydroxycinnamic_acid_esters -0.6834783
## 111 sporulation_capacity hydroxycinnamic_acid_esters  0.6843478
```

```
## 118      total_phenolics hydroxycinnamic_acid_esters  0.6966732
## 119           salicinoid hydroxycinnamic_acid_esters  0.8139130
## 120             flavonol hydroxycinnamic_acid_esters  0.8791304
## 135             flavonol                   flavan3ol -0.7834783
##                  p     adjusted
## 9   3.708598e-08 2.521847e-06
## 13  4.612599e-05 5.702849e-04
## 36  1.799033e-07 8.155616e-06
## 44  8.151910e-06 1.385825e-04
## 45  9.382600e-07 2.552067e-05
## 69  1.245372e-05 1.881896e-04
## 71  1.413677e-03 9.613006e-03
## 76  1.414401e-04 1.373990e-03
## 81  6.086815e-05 6.898390e-04
## 91  7.688471e-05 8.043324e-04
## 94  3.290656e-04 2.486273e-03
## 103 5.793123e-04 4.146656e-03
## 105 1.476022e-05 2.007390e-04
## 108 6.623409e-07 2.251959e-05
## 110 2.317540e-04 1.854032e-03
## 111 2.258819e-04 1.854032e-03
## 118 1.555306e-04 1.410144e-03
## 119 1.312388e-06 2.974747e-05
## 120 1.568836e-08 2.133617e-06
## 135 5.962930e-06 1.158512e-04
```

Adjusted P-values on Spearman correlation coefficients highlight significant correlation between :
- all sugars,
- salicin and total_phenolics (precursor of and defense components respectively),
- fructose and total_phenolics,
- total_phenolics and uredinai size,
- total_phenolics and sporulation rate,
- total_phenolics and sporulation capacity,
- uredinia size and sporulation rate,
- uredinia size and sporulation capacity.

Biplots on correlated data

```
# Aggregate fungal data
results$LPI <- as.numeric(as.character(results$LPI))
resag2 <- aggregate(results[c('LPI','infection_efficiency','latent_period',
'uredinia_size', 'spore_volume', 'sporulation_rate', 'mycelium_quantity', '
sucrose', 'salicin', 'glucose', 'fructose', 'C', 'N', 'salicinoid', 'flavon
ol', 'hydroxycinnamic_acid_esters', 'flavan3ol', 'total_phenolics')],result
s[c('leaf_id')], function(a) c(mean(a, na.rm=T)))
```

```
# add traits standard error to data
resag2$us_se <- NA
resag2$sp_rate_se <- NA
for (i in 1:nrow(resag2)){ # se for each ranplan from results
  resag2$us_se[i] <- se(results[which(results$leaf_id == resag2$leaf_id[i])
,]$uredinia_size, na.rm=T)
}

for (i in 1:nrow(resag2)){ # se for each ranplan from results
  resag2$sp_rate_se[i] <- se(results[which(results$leaf_id == resag2$leaf_i
d[i]),]$sporulation_rate, na.rm=T)
}

## Biplot uredinia size
# with phenol
mod <- lm(uredinia_size~total_phenolics,data=resag2)
ggplot(data=resag2,aes(x=total_phenolics,y=uredinia_size,color=LPI)) +
  geom_point() +
  scale_colour_gradient(low="blue",high = "red") +
  geom_errorbar(aes(ymin=resag2$uredinia_size - resag2$us_se,
                    ymax=resag2$uredinia_size + resag2$us_se)) +
  ylab('uredinia_size (mm2)') +
  xlab('total_phenolics (mg.g DW-1)') +
  geom_text(label = paste('R2 = ',round(summary(mod)$adj.r.squared,2)), x=5
0, y=0.8, color='black') # x, y, label, alpha, angle, color, family, fontfa
ce, hjust,  lineheight, size, vjust
```

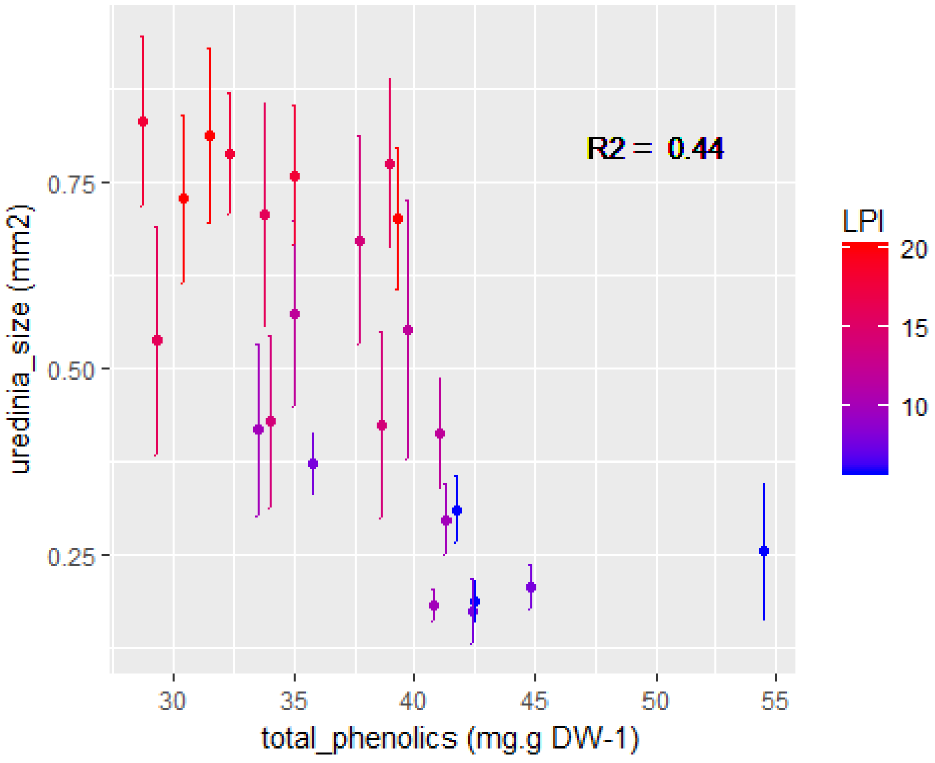

```
# without Plant1 LPI6 (outlier)
mod <- lm(uredinia_size~total_phenolics,data=resag2[which(resag2$leaf_id !=
'lpi6pl1'),])
ggplot(data=resag2[which(resag2$leaf_id != 'lpi6pl1'),], aes(x=total_phenol
ics,y=uredinia_size,color=LPI)) +
  geom_point() +
  scale_colour_gradient(low="blue",high = "red") +
  geom_errorbar(aes(ymin=resag2[which(resag2$leaf_id != 'lpi6pl1'),]$uredin
ia_size - resag2[which(resag2$leaf_id != 'lpi6pl1'),]$us_se,
                    ymax=resag2[which(resag2$leaf_id != 'lpi6pl1'),]$uredin
ia_size + resag2[which(resag2$leaf_id != 'lpi6pl1'),]$us_se)) +
  geom_text(label = paste('R2 = ',round(summary(mod)$adj.r.squared,2)), x=4
2.5, y=0.8, color='black')
```

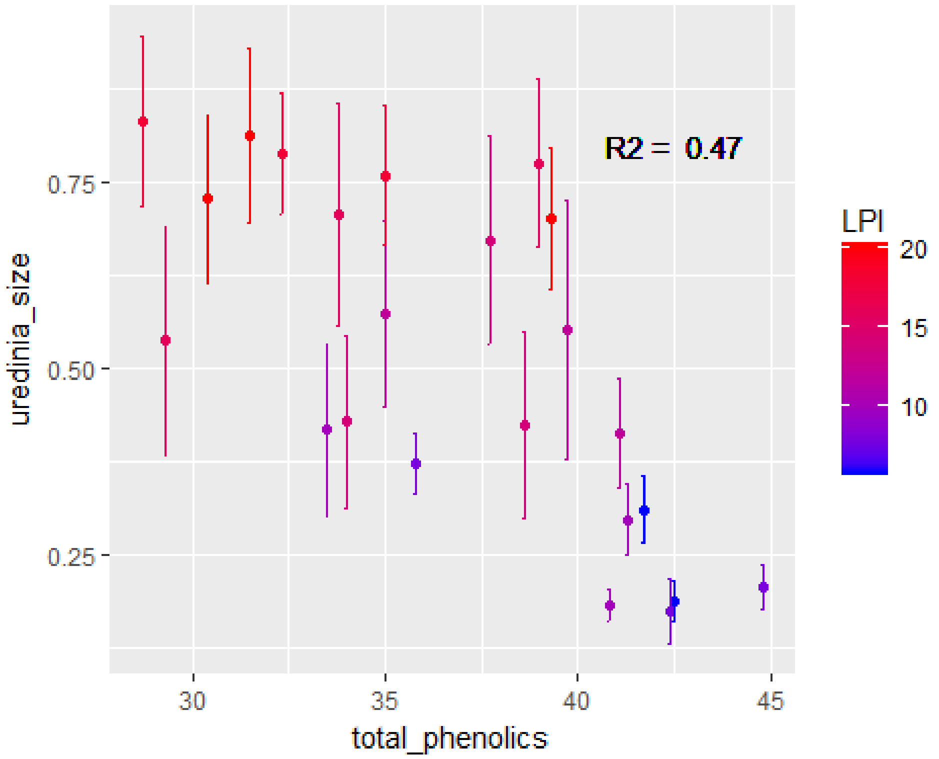


```
# with hydroxycinnamic_acid_esters
mod <- lm(uredinia_size~hydroxycinnamic_acid_esters,data=resag2)
ggplot(data=resag2,aes(x=hydroxycinnamic_acid_esters,y=uredinia_size,color=
LPI)) +
  geom_point() +
  scale_colour_gradient(low="blue",high = "red") +
  geom_errorbar(aes(ymin=resag2$uredinia_size - resag2$us_se,
                    ymax=resag2$uredinia_size + resag2$us_se)) +
  ylab('uredinia_size (mm2)') +
  xlab('hydroxycinnamic_acid_esters (mg.g DW-1)') +
  geom_text(label = paste('R2 = ',round(summary(mod)$adj.r.squared,2)), x=5
000, y=0.8, color='black') # x, y, label, alpha, angle, color, family, font
face, hjust,  lineheight, size, vjust
```

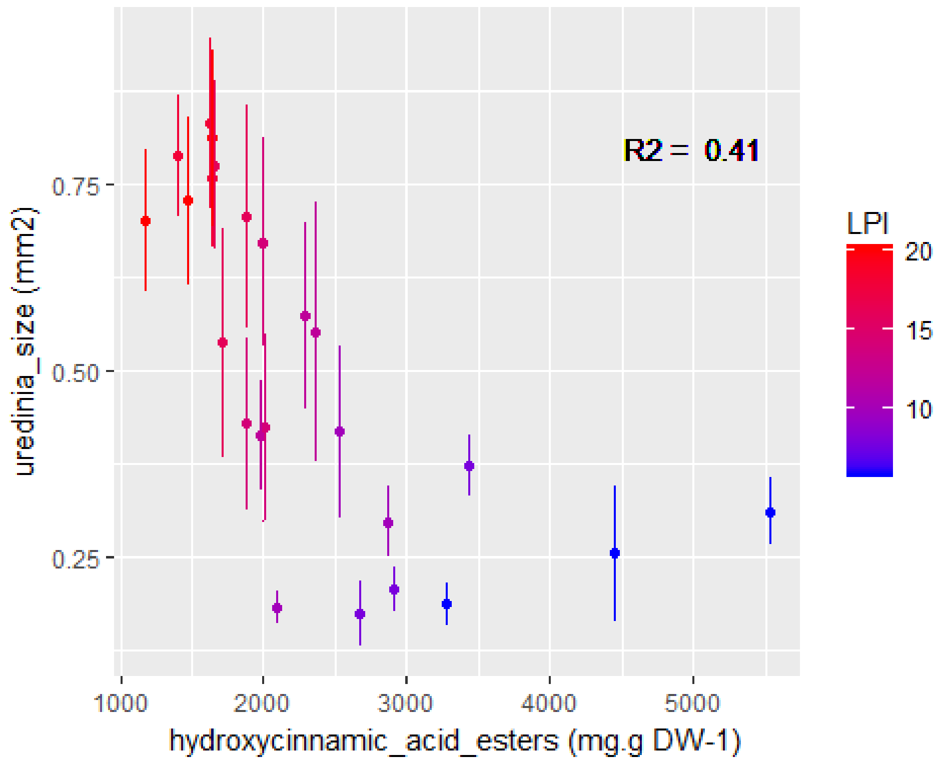


```
# without Plant1 LPI6 (outlier)
mod <- lm(uredinia_size~total_phenolics,data=resag2[which(resag2$leaf_id !=
'lpi6pl1'),])
ggplot(data=resag2[which(resag2$leaf_id != 'lpi6pl1'),], aes(x=total_phenol
ics,y=uredinia_size,color=LPI)) +
  geom_point() +
  scale_colour_gradient(low="blue",high = "red") +
  geom_errorbar(aes(ymin=resag2[which(resag2$leaf_id != 'lpi6pl1'),]$uredin
ia_size - resag2[which(resag2$leaf_id != 'lpi6pl1'),]$us_se,
                    ymax=resag2[which(resag2$leaf_id != 'lpi6pl1'),]$uredin
ia_size + resag2[which(resag2$leaf_id != 'lpi6pl1'),]$us_se)) +
  geom_text(label = paste('R2 = ',round(summary(mod)$adj.r.squared,2)), x=4
2.5, y=0.8, color='black')
```

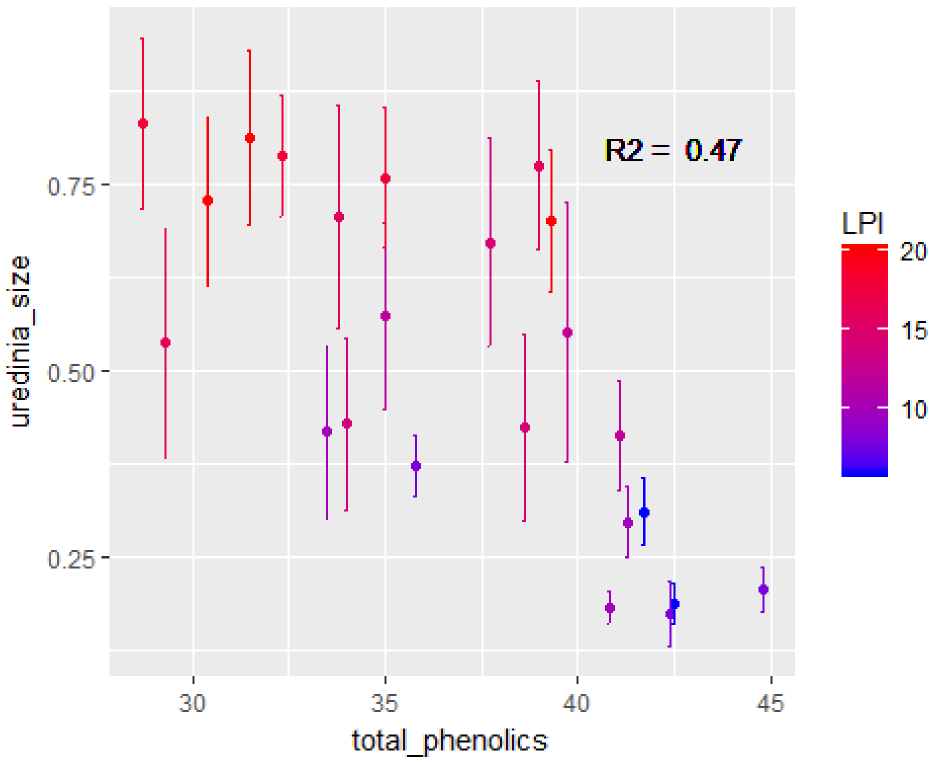


```
## Biplot sporulation rate
# with phenolics
mod <- lm(sporulation_rate~total_phenolics,data=resag2)
ggplot(data=resag2,aes(x=total_phenolics,y=sporulation_rate,color=LPI)) +
  geom_point() +
  scale_colour_gradient(low="blue",high = "red") +
  geom_errorbar(aes(ymin=resag2$sporulation_rate - resag2$sp_rate_se,
                    ymax=resag2$sporulation_rate + resag2$sp_rate_se)) +
  ylab('sporulation rate (urediniospore/day)') +
  xlab('total_phenolics (mg.g DW-1)') +
  geom_text(label = paste('R2 = ',round(summary(mod)$adj.r.squared,2)), x=5
0, y=750, color='black')
```

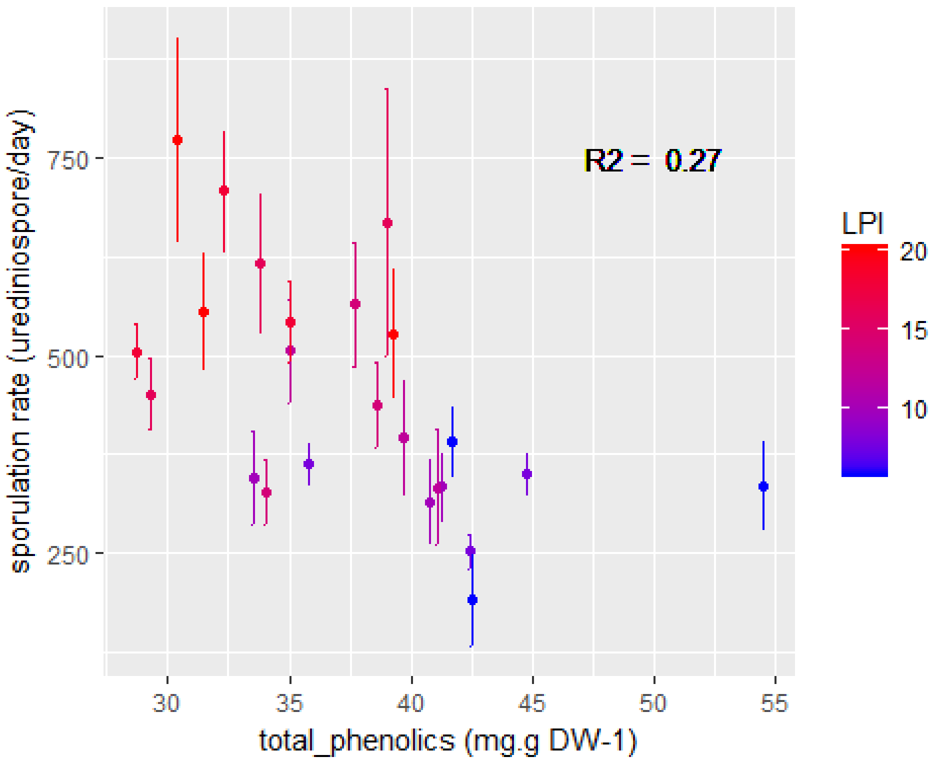


```
# without Plant1 LPI6 (outlier)
mod <- lm(sporulation_rate~total_phenolics,data=resag2[which(resag2$leaf_id
!= 'lpi6pl1'),])
ggplot(data=resag2[which(resag2$leaf_id != 'lpi6pl1'),],aes(x=total_phenoli
cs,y=sporulation_rate,color=LPI)) +
  geom_point() +
  scale_colour_gradient(low="blue",high = "red") +
  geom_errorbar(aes(ymin=resag2[which(resag2$leaf_id != 'lpi6pl1'),]$sporul
ation_rate - resag2[which(resag2$leaf_id != 'lpi6pl1'),]$sp_rate_se,
                    ymax=resag2[which(resag2$leaf_id != 'lpi6pl1'),]$sporul
ation_rate + resag2[which(resag2$leaf_id != 'lpi6pl1'),]$sp_rate_se)) +
  geom_text(label = paste('R2 = ',round(summary(mod)$adj.r.squared,2)), x=4
2.5, y=750, color='black')
```

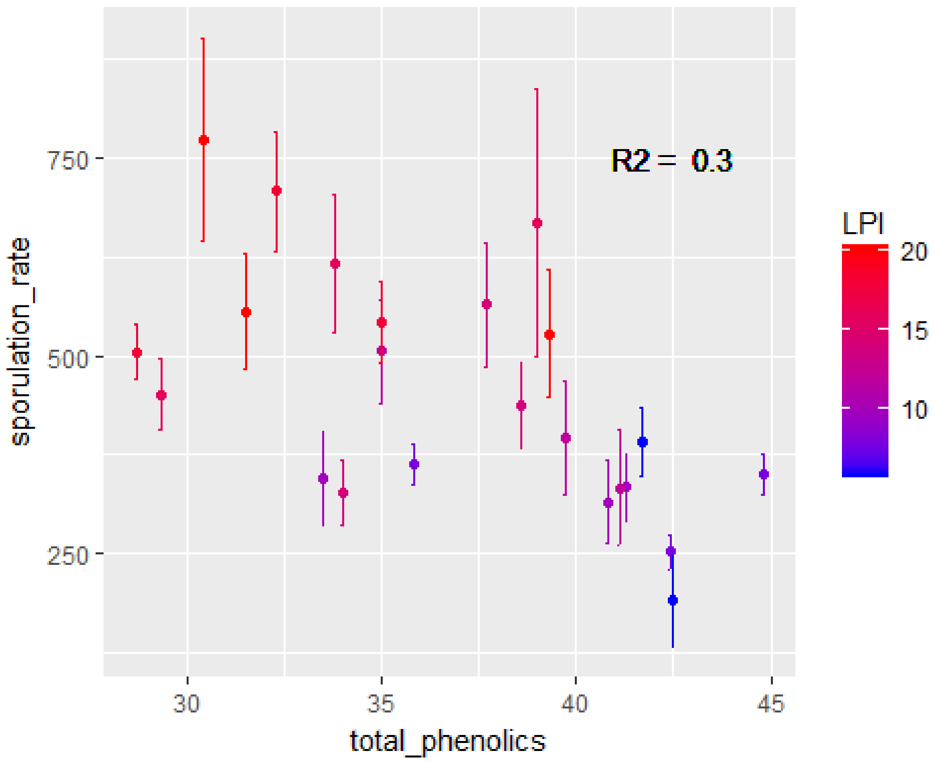


```
# with hydroxycinnamic_acid_esters
mod <- lm(sporulation_rate~hydroxycinnamic_acid_esters,data=resag2)
ggplot(data=resag2,aes(x=hydroxycinnamic_acid_esters,y=sporulation_rate,col
or=LPI)) +
  geom_point() +
  scale_colour_gradient(low="blue",high = "red") +
  geom_errorbar(aes(ymin=resag2$sporulation_rate - resag2$sp_rate_se,
                    ymax=resag2$sporulation_rate + resag2$sp_rate_se)) +
  ylab('sporulation_rate (urediniospore/day)') +
  xlab('hydroxycinnamic_acid_esters (mg.g DW-1)') +
  geom_text(label = paste('R2 = ',round(summary(mod)$adj.r.squared,2)), x=5
000, y=750, color='black')
```

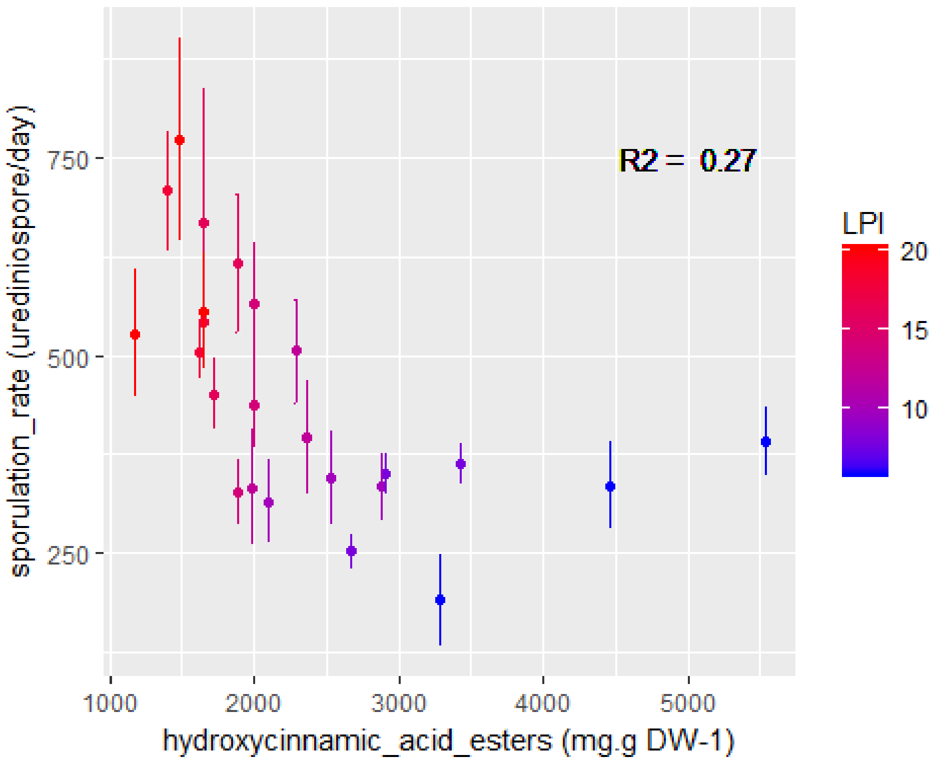


```
# without Plant1 LPI6 (outlier)
mod <- lm(sporulation_rate~total_phenolics,data=resag2[which(resag2$leaf_id
!= 'lpi6pl1'),])
ggplot(data=resag2[which(resag2$leaf_id != 'lpi6pl1'),],aes(x=total_phenoli
cs,y=sporulation_rate,color=LPI)) +
  geom_point() +
  scale_colour_gradient(low="blue",high = "red") +
  geom_errorbar(aes(ymin=resag2[which(resag2$leaf_id != 'lpi6pl1'),]$sporul
ation_rate - resag2[which(resag2$leaf_id != 'lpi6pl1'),]$sp_rate_se,
                    ymax=resag2[which(resag2$leaf_id != 'lpi6pl1'),]$sporul
ation_rate + resag2[which(resag2$leaf_id != 'lpi6pl1'),]$sp_rate_se)) +
  geom_text(label = paste('R2 = ',round(summary(mod)$adj.r.squared,2)), x=4
2.5, y=750, color='black')
```

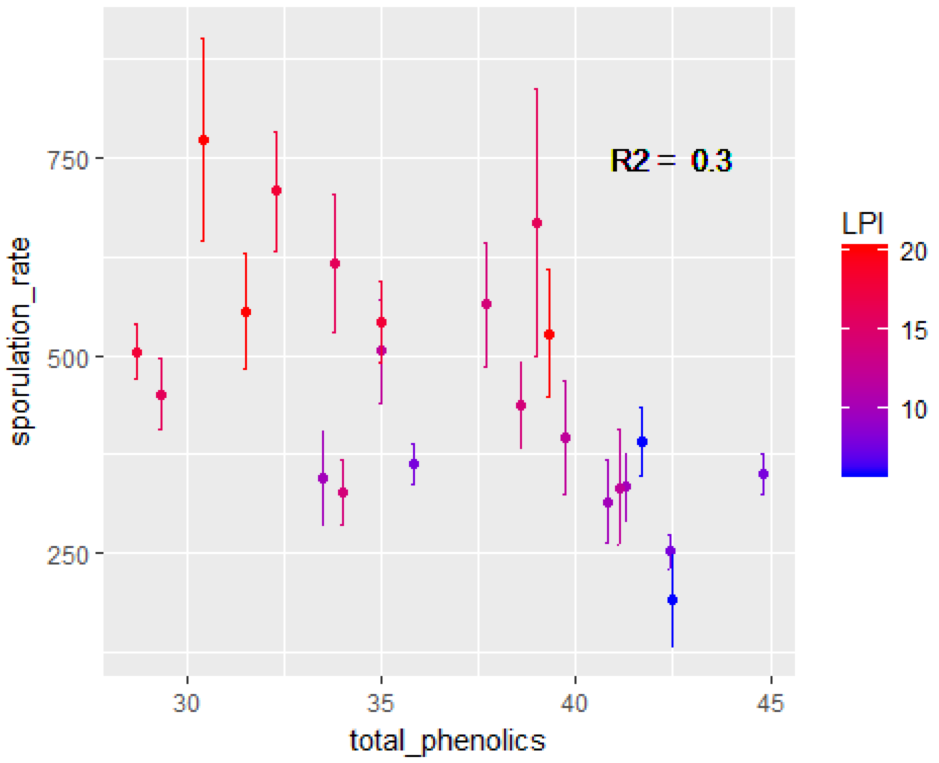

R2 = 0.3
LPI
20
15
10
750
500
250
30
35
40
45
sporulation_rate
total_phenolics
